# Microwave-enhanced additive-free C–H amination of benzoxazoles catalysed by supported copper

**DOI:** 10.3762/bjoc.21.108

**Published:** 2025-07-15

**Authors:** Andrei Paraschiv, Valentina Maruzzo, Filippo Pettazzi, Stefano Magliocco, Paolo Inaudi, Daria Brambilla, Gloria Berlier, Giancarlo Cravotto, Katia Martina

**Affiliations:** 1 Department of Drug Science and Technology, University of Turin, Via Pietro Giuria 9, 10125 Turin, Italy; 2 Present address: R&D Department, Farmabios S.P.A., Via Pavia 1, Gropello Cairoli, Italy; 3 Department of Chemistry, NIS Interdepartmental Centre and INSTM Reference Centre, University of Turin, Via Pietro Giuria 7, 10125 Turin, Italy; 4 Cube Labs Spa V. Giulia Caccini 1, 00198 Roma, Italy

**Keywords:** aerobic oxidation, copper, grafted silica, heterogeneous catalysis, microwave

## Abstract

The C2-amination of benzoxazole offers wide-ranging potential for substrate expansion and the functionalisation of bioactive compounds. This study presents a green and efficient C–H amination, catalysed by CuCl and CuCl_2_, in acetonitrile without acidic, basic or oxidant additives that is accelerated by microwave (MW) irradiation and is completed in 1.5–2 h. A solid Cu(I) catalyst supported on aminated silica made the process cost-effective and heterogeneous, thus simplifying work-up and minimising free copper in solution. The catalyst was found to be regeneratable and reusable for up to eight cycles. The optimised method facilitated the synthesis of various benzoxazole derivatives, demonstrating its versatility and practical applicability.

## Introduction

2-Aminoazoles are nitrogenous heterocyclic compounds of high relevance due to their biological and pharmaceutical activity and their importance within the materials sciences [1–2]. 2-Aminobenzoxazoles, in particular, are important building blocks in the development of new bioactive compounds that can be useful as therapeutic agents with antibacterial [3], antiviral, antifungal [4], anticancer [5–7] and anti-inflammatory activity [8]. Moreover, they can be applied in disorders of the central nervous system, such as insomnia and Alzheimer’s disease [9–10].

The preparation of 2-aminobenzoxazoles classically proceeds via cyclocondensation reactions from pre-functionalised precursors, or via the C2-amination of benzoxazoles by transition-metal-catalysed reactions that traditionally involve aryl halide scaffolds [11–14]. However, these procedures entail disadvantages that need to be overcome if green chemistry criteria are to be met; high temperatures, long reaction times, the need for ligands, and the huge overall economic impact of these processes.

Inspired by the logic behind cross-dehydrogenative C–C-coupling methods [15], the direct C–H amination has been developed as a more straightforward, economical and environmentally friendly reaction, compared to its counterparts (such as the classical Buchwald–Hartwig amination reaction [16] or the Ullman reaction [17–18]) which require pre-functionalisation steps and harsh conditions [19].

Many protocols for C–H aminations have been applied to heteroaromatic compounds, mainly 5-membered heteroarenes, and a great deal of attention has been devoted to benzoxazoles. When applied to benzoxazole, the reaction is catalysed by transition metals such as Ag(I) [20], Mn(II) [21], Fe(III) [22–23], Co [24], Ni(II) [25] and Fe(III) [26]. However, the use of Cu(II) has increased thanks to its high tolerance towards several functional groups, high environmental abundance, low cost and low overall toxicity. A wide range of aminating reagents have been utilised, including nitrogen electrophiles and amines in the presence of external or internal oxidants [27], in many types of copper-catalysed synthetic protocols. The direct copper-catalysed C–H amination of azoles was pioneered by Mori [28], Schreiber [29], and Zhao [30]. However, high reaction temperatures and a large amount of base or acid dramatically decrease atom economy (the percentage of reactant atoms incorporated into the desired product) and functional-group tolerance, while the use of an oxidant considerably impacts upon the sustainability of the process because it involves toxic reagents, generates hazardous by-products, increases the overall reaction cost and increases waste production [30–32]. In subsequent years, other base-catalysed protocols have been developed for the reaction of azolic substrates, but here the amines and the ligands are still used in excess and auxiliary oxidants are occasionally employed [33–35]. Although electrophilic amines, such as chloroamines [36–37], hydroxylamine [38–40], acylated hydroxylamine (with a wider reaction scope) [41–44] and sulfamoyl chlorides [32] can perform the coupling under basic conditions, the need for the activation of the amine as an electrophilic agent generates additional waste. This reduces atom economy and indicates lower reaction efficiency.

Acid-catalysed protocols have also been specifically developed for the amination of oxazoles, with many of them utilizing aerobic oxidation to improve the sustainability of the process. Indeed, in 2011, Guo et al. [45] developed a protocol for the direct C–H amination of benzoxazoles and oxadiazoles, under an O_2_ atmosphere using 20 mol % of a Cu(II) catalyst, that still required high temperatures, but the excess of amine (2 equiv) was lowered and only a catalytic amount of acid was utilised. Similar acidic protocols were subsequently developed by Li et al. [46], in which benzoxazoles were reacted with secondary amines and amides, with higher temperature being applied when reacting amides to achieve their decarbonylation. In 2014, Cao et al. [47] reported the amination of benzoxazole with a secondary amine either in air or an O_2_ atmosphere, lowering the catalyst amount and the reaction temperature.

In 2020, a study by De Vos and co-workers [48] focused on developing a new additive-free protocol, catalysed by a copper catalyst supported on acidic zeolites that efficiently catalysed C2-benzoxazole amination in the presence of a perfluorinated solvent (hexafluoroisopropanol) as a source of mobile protons. Despite the significant interest in this area, to the best of our knowledge, this study represents one of the few examples of a heterogeneous catalysed copper-mediated C–H amination of benzoxazole.

**Scheme 1:**
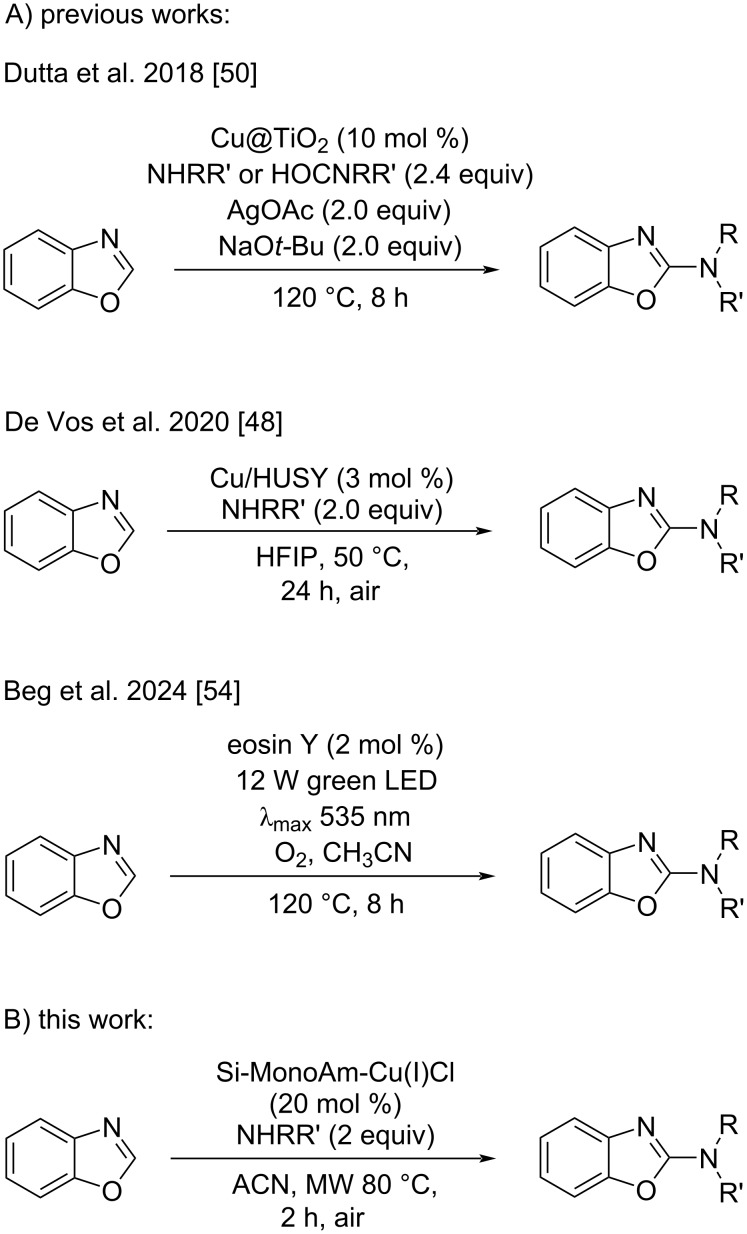
Representative synthetic routes for the C–H amination of benzoxazole using supported copper catalysts.

The pursuit for greener methodologies in organic synthesis and transitioning from traditional homogeneous catalysis to the use of heterogeneous catalysts for direct C–H amination processes could be a significant breakthrough in optimising these reactions. Despite recent progress in site-selective C–H functionalisation [49], most reactions have remained reliant on homogeneous catalysis due to its molecularly defined nature. By contrast, the development of heterogeneous catalysis has faced challenges, due to uncertainties around catalytic sites, aggregation, and the leaching of active species during reactions. The use of a heterogeneous catalyst can greatly simplify the work-up process, as the catalyst can be easily removed via simple filtration. Additionally, using supported metals instead of metal salts can prevent the formation of chelates, which might otherwise impact the efficiency of the procedure. Moreover, this approach can provide bifunctional catalysis as the support itself contributes to the catalyst's reactivity, thus enhancing its overall efficiency. In the context of the C2 amination of azoles, to the best of our knowledge, only a few studies [48,50] have explored this approach, suggesting significant opportunities for further development and improvement. Building on our previous experience in the preparation and characterisation of supported copper(II) catalysts on covalently modified silica [51–52], we have set out to develop a new heterogeneous catalyst with atomically distributed active sites for the mild and efficient C–H amination of benzoxazole. This approach was chosen because the silica derivatisation and copper deposition methods are simple, inexpensive and scalable, making the catalyst reliable and suitable for large scale production. This study aims to optimise an efficient, user-friendly and heterogeneously catalysed procedure that enables the rapid synthesis of 2-aminobenzoxazole derivatives. A key focus is leveraging microwave irradiation to enhance the protocol’s efficacy. The non-thermal effects and unique ability of MW irradiation to promote reactions catalysed by solid-supported metals have already been demonstrated [53]. As shown in Scheme 1, our research is in line with earlier studies that highlight the advantages of heterogeneous catalysis, but compared to other approaches, we aimed at eliminating the presence of bimetallic catalysts, the addition of a base [50], and the use of costly perfluorinated protic solvents [48]. In addition, this approach offers improved performance compared to photochemical C–H amination reactions of benzoxazole, which, although metal-free, are still limited in versatility and require photocatalysts such as eosin Y and a tightly controlled O_2_ atmosphere [54]. The objective of the present study aims to provide a rapid, additive-free and convenient alternative to existing approaches in this field, which have attracted considerable interest over the years.

## Results and Discussion

Inspired by the extensive literature on C2-benzoxazole amination, we initially began our optimisation by studying a homogeneous procedure in the presence of piperidine, which was used as amine component to identify mild conditions for the Cu-catalysed aerobic C–H amination in absence of acid, base or co-catalyst additives. As described in Table 1, we explored the effects of different solvents and copper species, and preliminary reactions were conducted with Cu(II) salts, with reference to reaction conditions already reported in the literature for aerobic protocols. As shown in Table 1, entries 1–3, we compared xylene, toluene, and acetonitrile as solvents. According to Bhanage and Wagh [13], Cu(II) catalyses the C–H amination in xylene at 140 °C for 14 hours under oxygen. We tested the reaction in refluxing toluene with 20 mol % of CuCl_2_ and observed a significantly decreased product yield (Table 1, entry 2). As Cao et al. [47] already have reported, acetonitrile improved the reaction efficiency, resulting in an increase in product yield to 78% when the reaction was performed at 80 °C. Interestingly, reflux conditions did not improve reaction conversion, as shown in entries 3 and 4 (Table 1) and we consistently observed a decrease in reaction yield. This effect is likely due to the lower solubility of O_2_ in the refluxing solvent, which may negatively impact the reaction outcome. As previously demonstrated, adding acetic acid enabled the reaction to proceed with even lower catalyst loading [47]. To avoid the use of acid and to minimise the amount of the amine, we repeated the reaction with 1.5 equivalents of piperidine, and compared different Cu(II) and Cu(I) salts. Both Cu(I) and Cu(II) chloride demonstrated good product conversion and selectivity without the need to perform the reaction under oxygen, while Cu(OAc)_2_ was slightly less efficient. When the catalyst loading was reduced to 15 mol %, the reaction yield decreased to approximately 75% for both copper salts. However, reducing the reaction time to 6 hours, with a catalyst loading of 20 mol %, resulted in complete conversion and high selectivity, comparable to the results obtained from an overnight reaction. Notably, decreasing the reaction temperature to 60 °C still yielded excellent results with both catalysts after an overnight reaction and after 6 hours, although conversion was still proceeding after 4 hours of reaction. Both catalysts performed comparably, with Cu(OAc)_2_ consistently showing slightly lower efficiency (Table 1, entries 14–20).

**Table 1:** C2-Amination of benzoxazole with piperidine via homogeneous catalysis.

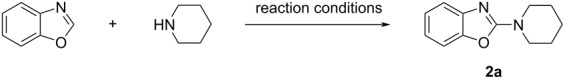					

Entry	Catalyst (mol %)	Amine (equiv)	Conditions^a^	Conversion (%)^b^	Yield (%)^b^

1	Cu(acac)_2_ (20)	2	xylene, 140 °C, 14 h, O_2_	>99	88, ref [13]
2	CuCl_2_ (20)	2	toluene, reflux, o.n.	82	28
3	CuCl_2_ (20)	2	acetonitrile, 80 °C, o.n,	96	78
4	CuCl_2_ (20)	2	acetonitrile, reflux, o.n.	83	49
5	CuBr_2_ (10)	1.2	2 equiv CH_3_COOH, acetonitrile, 50 °C, o.n.	>99	95, ref [47]
6	CuCl_2_ (20)	1.5	acetonitrile, 80 °C, o.n.	92	81
7	Cu(OAc)_2_ (20)	1.5	acetonitrile, 80 °C, o.n.	96	74
8	CuCl (20)	1.5	acetonitrile, 80 °C, o.n.	>99	83
9	CuCl_2_ (15)	1.5	acetonitrile, 80 °C, o.n.	89	75
10	CuCl (15)	1,5	acetonitrile, 80 °C, o.n.	96	74
11	CuCl_2_ (20)	1.5	acetonitrile, 80 °C, 6 h	90	83
12	CuCl (20)	1.5	acetonitrile, 80 °C, 6 h	96	87
13	CuCl (10)	1.5	acetonitrile, 80 °C, 6 h	83	36
14	CuCl_2_ (20)	1.5	acetonitrile, 60 °C, o.n.	96	91
15	Cu(OAc)_2_ (20)	1.5	acetonitrile, 60 °C, o.n.	99	77
16	CuCl (20)	1.5	acetonitrile, 60 °C, o.n.	95	88
17	CuCl_2_ (20)	1.5	acetonitrile, 60 °C, 6 h	97	90
18	CuCl (20)	1.5	acetonitrile, 60 °C, 6 h	93	86
19	CuCl_2_ (20)	1.5	acetonitrile, 60 °C, 4 h	78	65
20	CuCl (20)	1.5	acetonitrile, 60 °C, 4 h	77	57

^a^Reaction conditions: benzoxazole (0.1 mmol), piperidine (see table), Cu catalyst, solvent (1 mL). ^b^Conversion and yield were measured by ^1^H NMR spectroscopy.

We then focused on studying the efficiency of MW-irradiation-promoted reactions, which, as reported in numerous green protocols, offer significant advantages over the use of conventional heating. The goal was to utilise selective, volumetric dielectric heating to save time and energy, enable selective catalysis and generally achieve higher selectivity and yields [53,55–56]. Building on the improvements made using conventional methods, we tested the reaction under MW irradiation (see Table 2).

**Table 2:** Microwave-promoted C2-amination of benzoxazole with piperidine.

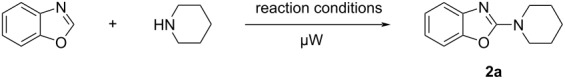				

Entry	Catalyst (mol %)	Conditions^a^	Conversion (%)^b^	Yield (%)^b^

1	CuCl_2_ (20)	60 °C, 2 h, open vessel, MW^c^	87	85
2	CuCl (20)	60 °C, 2 h, air, MW^c^	>99	98
3	CuCl (20)	60 °C, 2 h, N_2_ 5 bar, MW^d^	77	13
4	CuCl (20)	60 °C, 1 h, air, MW^c^	81	75
5	CuCl (15)	60 °C, 2 h, air, MW^c^	75	71
6	CuCl (10)	60 °C, 2 h, air, MW^c^	63	45
7	CuCl (20)	60 °C, 2 h, closed vial, MW^e^	79	58

^a^Reaction conditions: benzoxazole (0.1 mmol), piperidine (0.2 mmol), Cu catalyst, acetonitrile (1 mL); ^b^conversion and yield were measured by NMR spectroscopy; ^c^Microsynth MW oven; ^d^SynthWave MW reactor; ^e^Anton Paar Monowave.

Two different pieces of equipment were evaluated: a flexible MW oven (Microsynth by Milestone) for traditional glassware synthesis and a MW reactor (SynthWave by Milestone) capable of handling any reaction temperature and gas pressure (up to 300 °C and 200 bar). The SynthWave reactor also allows multiple gases to be loaded, including both inert and reactive gases. As shown in Table 2, MW irradiation significantly reduced reaction times from 6 hours to 2 hours under atmospheric air conditions. At 60 °C with 20 mol % of CuCl_2_, an 87% product yield was obtained, while the use of CuCl enhanced reactivity, providing a nearly quantitative yield of 98% (Table 2, entries 1 and 2).

When the reaction was conducted in the SynthWave reactor, pressurised with 5 bar of nitrogen, the yield of desired product dropped to 13%, highlighting the importance of oxygen/air in the oxidative aromatisation of the benzoxazole ring. Reducing the reaction time to 1 hour or lowering the catalyst loading to 15 mol % resulted in decreased yields of 75% and 71%, respectively.

The reaction was also carried out in Anton Paar equipment with the vessel pressurised under nitrogen (Table 2, entry 7). Under these conditions, the yield was 58%, demonstrating the superiority of an open vessel in a multimode cavity for this reaction.

**Figure 1:**
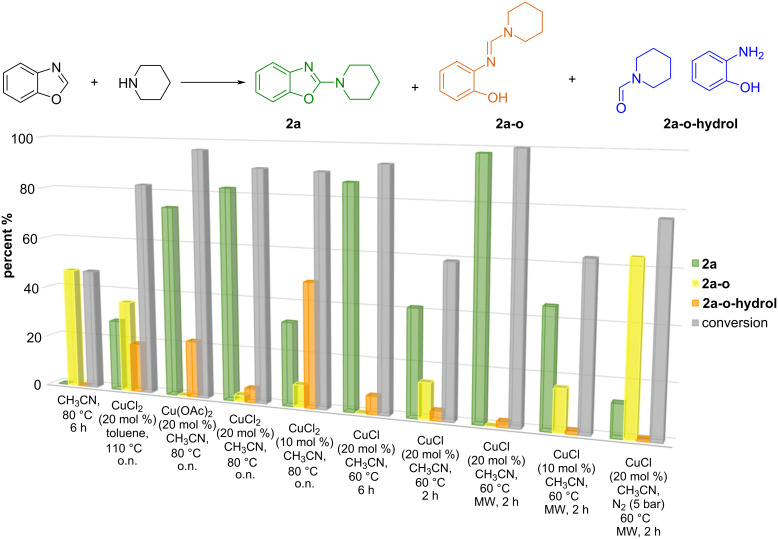
Reaction of benzimidazole with piperidine. a) Reaction scheme including intermaidates and b) conversion and selectivity plot of the C2-amination of benzoxazole with piperidine. Reaction conditions: benzoxazole (0.1 mmol), piperidine (0.15 mmol), copper catalyst, solvent (1.0 mL). Conversion was measured by NMR spectroscopy (see Figure S1 in Supporting Information File 1 for an example).

Since Cu salts facilitate both the nucleophilic attack of piperidine on benzoxazole to form the intermediate open derivative, and the subsequent ring oxidation upon closure (as shown in the mechanism in Scheme S1 (Supporting Information File 1), the reaction mixture may contain the desired 2-substituted benzoxazole **2a** together with its open-form precursor **2a-o**. The latter can be hydrolysed to give *N*-formylpiperidine and aminophenol (**2a-o-hydrol**) (see Figure 1a). To better understand the influence of the reaction conditions on product distribution, we plotted conversion versus selectivity in Figure 1b. Crudes were analysed by ^1^H NMR spectroscopy, as shown in Figure S1 (Supporting Information File 1), to determine the percentage composition of starting material and products **2a**, **2a-o** and **2a-o-hydrol**. The reaction was perfectly reproducible and no other derivatives were observed.

As shown in Figure 1b, when the reaction was conducted without a catalyst, only ring opening was observed, accounting for 47%. By contrast, the presence of Cu facilitated the conversion of the intermediate to the desired aromatic benzoxazole **2a**, demonstrating copper’s dual role as a Lewis acid in enhancing nucleophilic attack, and as an efficient catalyst for ring-closing oxidative rearomatisation.

Of the conditions tested, the use of CuCl_2_ in toluene showed limited effectiveness, achieving 82% conversion but only a 28% yield of the final product. The remaining resulting mixture included 35% of the intermediate **2a-o** and 19% of the hydrolysed product **2a-o-hydrol**. However, the reaction performance in acetonitrile improved significantly, with CuCl_2_ yielding 82% of the desired product, alongside 2.4% of **2a-o** and 5.4% of **2a-o-hydrol**. Reducing the catalyst amount from 20 mol % to 10 mol % did not affect conversion, but resulted in a lower yield of **2a** and increased production of **2a-o-hydrol** to 48%, indicating that insufficient catalyst at 80 °C overnight favours side reactions, compromising selectivity.

When using CuCl (20 mol %), an excellent selectivity was observed at 60 °C. Although conversion was only 59% after 2 hours, full reaction completion was observed in 6 hours. MW irradiation enhanced both the reaction rate and selectivity, with the reaction being completed after 2 hours at 60 °C affording a 98% yield of the desired product. We observed only trace amounts of **2a-o-hydrol** (1.2%) even at a reduced catalyst amount of 10 mol % CuCl. We hypothesise that, under MW, the hydrolysis side reaction is mitigated due to the mild reaction conditions both in terms of temperature and time, as observed using a lower catalyst amount. This assumption was confirmed when the reaction was performed in a MW reactor under 5 bar of N_2_; in this environment, the copper catalyst cannot regenerate because of the absence of oxygen (see mechanism Scheme S1 in Supporting Information File 1). The ^1^H NMR spectrum of the crude showed that the starting material was almost completely converted (by 77.4%), that the intermediate **2a-o** was recovered in the mixture at 64% and that the side product was detected at only 0.7%.

**Figure 2:**
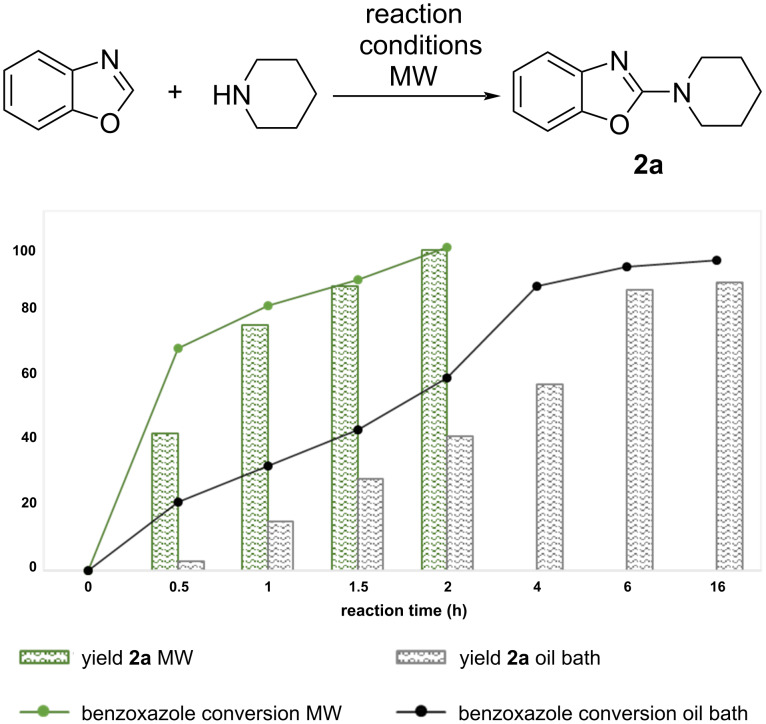
Reaction rate comparison between conventional (oil bath) and MW heating. Reaction conditions: benzoxazole (0.1 mol), piperidine (0.15 mmol), copper catalyst 20 mol %, solvent (1.0 mL). Conversion was measured by ^1^H NMR spectroscopy.

To more deeply understand the influence of MW irradiation in the enhancement of the reaction rate for the CuCl-catalysed reaction, we carried out the model reaction in parallel, using both an oil bath and MW irradiation at 60 °C. Figure 2 clearly shows that MW irradiation drives the reaction to completion in 2 hours. By contrast, the conventional method results in a slower rate for the conversion of the starting benzoxazole to the open intermediate **2a-o**, and the subsequent ring closing aromatisation to product **2a**. As can be seen in Figure 2, a complete conversion is achieved in 6 hours under conventional conditions. Our observations indicate that MW irradiation enhances the catalytic activity of Cu toward the oxidative aromatisation of **2a-o**, leading to the fast and complete conversion of the starting material to the desired 2-(piperidine-1-yl)benzoxazole (**2a**). Since both reactions were conducted at the same temperature, this experiment confirms that MW irradiation activates the catalyst independently of bulk temperature, as reported in previous studies [57–58]. We can hypothesise that MW heating is likely associated with the generation of hot spots on metal sites, resulting in an accelerated reaction rate and improved selectivity for the desired product.

Despite the advantages of the developed protocol, the homogeneously catalysed procedure negatively impacted the purification and recovery of the products because of the presence of copper salts and complexes. We observed that multiple aqueous washes of the reaction crude are required to remove copper salts, and aqueous ammonia has been used to improve the efficacy of liquid–liquid extraction (see Supporting Information File 1 and experimental procedure). This issue made liquid–liquid extraction time-consuming and wasteful, while chromatographic column purification is also required to obtain a pure final product.

**Scheme 2:**
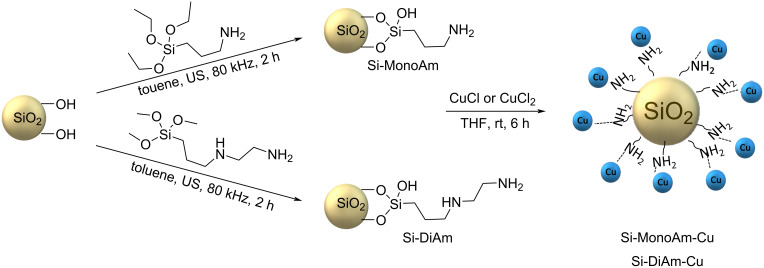
Graphical representation of Si-MonoAm-Cu(I) and Si-DiAm-Cu(I) preparation.

To overcome this problem, a heterogeneous catalytic approach has been developed in which copper is anchored on an aminated silica support. Given that ammonia, amino derivatives and other nitrogen-containing compounds form strong coordination complexes with transition metals like copper [59–60], we decided to graft an amino derivative onto the surface of silica, based on our previous experience [51–52]. This covalently modified support was then used to stably bind Cu(I) and Cu(II) species (see Scheme 2).

**Figure 3:**
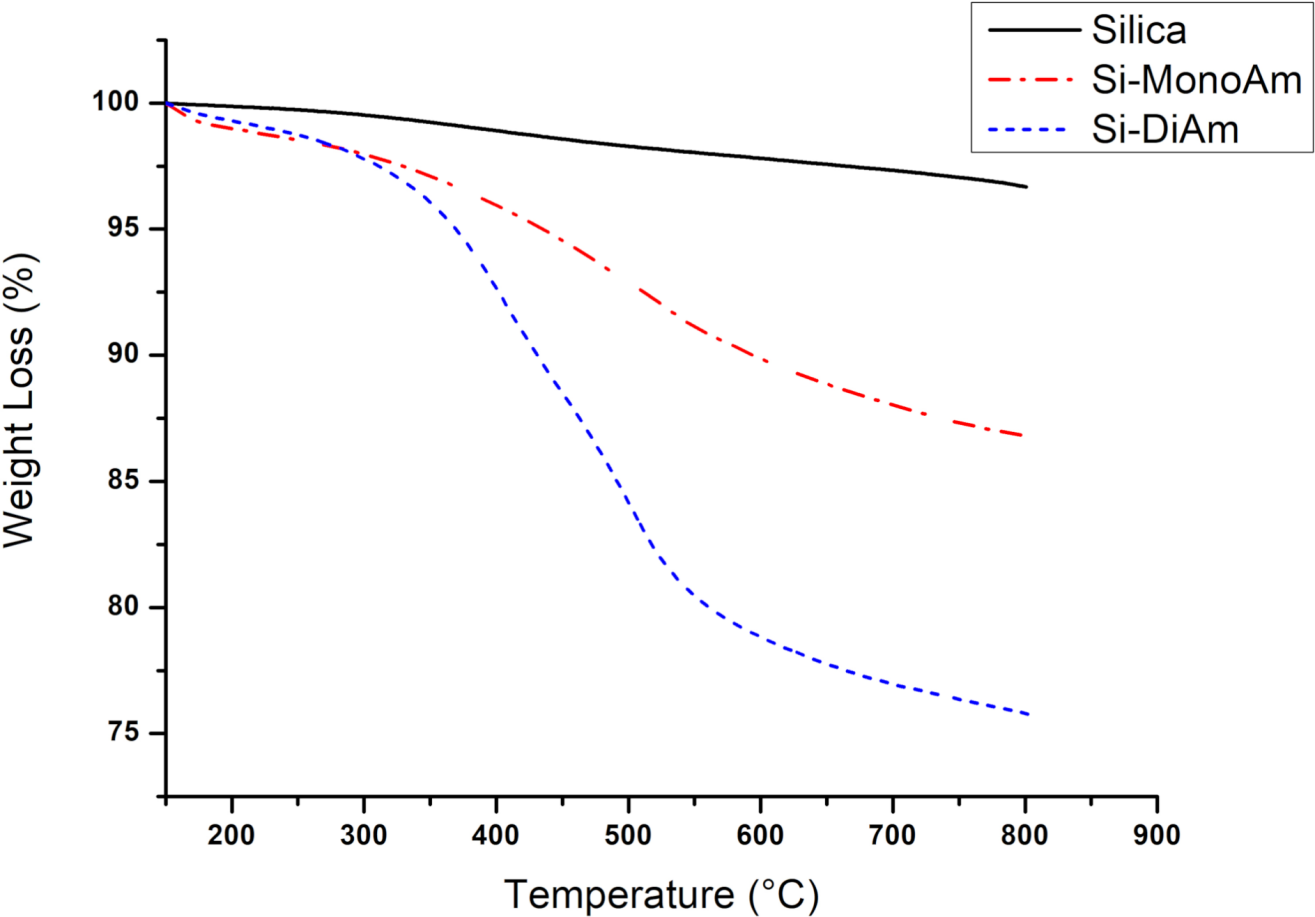
TGA profiles of SIPERNAT silica and Si-MonoAm and Si-DiAm.

Silica SIPERNAT 320 (Evonik) was selected because of its moderate absorption capacity and specific surface area (SSA), measured at 164 m^2^/g using the BET surface area analysis, and because it can be efficiently derivatised with a trialkoxysilane amino derivative as recently reported [61]. A previously optimised ultrasound-promoted synthetic protocol was employed to efficiently graft either 3-aminopropyltriethoxysilane (MonoAm) or 3-(2-aminoethylamino)propyltrimethoxysilane (DiAm) (Scheme 2) [51], and the efficiency of derivatisation was measured by thermogravimetric analysis (TGA) as showed in Figure 3. TGA curves were all normalised to 150 °C to circumvent any possible solvent influence on yield calculations and both Si-MonoAm and Si-DiAm showed a high degree of derivatisation; 9.9 and 21 wt %, respectively, which correspond to 738 and 1090 μmol/g. The first derivative peak temperatures that indicate the point of the greatest rate of change in the weight-loss curve were consistent in both samples and detected to be 446 °C in Si-MonoAm and 448 °C in Si-DiAm.

Both Si-MonoAm and Si-Diam were loaded with either CuCl or CuCl_2_ by exposing the derivatised silica to a solution of the corresponding Cu salt in THF, following a procedure previously reported in the literature [62–63]. The suspension was stirred magnetically for 6 hours at room temperature, resulting in the formation of a blue-coloured catalyst. We aimed to vary the amount of copper loaded onto the silica surface to achieve different catalyst loadings (Cu wt %) and compared their catalytic activities. The Cu salts reacted with the amino groups grafted onto the silica surfaces of Si-MonoAm and Si-DiAm, resulting in a theoretical copper content of 3, 5 and 9 wt % on the surface at the end of the reaction (see Table S1 in Supporting Information File 1).

The supported catalysts were subsequently tested and we performed preliminary reactions with Cu(I or II) 5 wt % supported on Si-MonoAm and Si-DiAm. We observed that the solid-supported catalysts consistently achieved complete conversion at 80 °C with Si-MonoAm and produced a good-to-quantitative yields of the desired product (see Table 3, entries 3 and 4). The Si-MonoAm support demonstrated superior efficiency over Si-DiAm, and supported Cu(I) performed better than Cu(II) (see Table 3, entries 3–6). At 60 °C, the reaction mixture contained the **2a-o** intermediate, and the yield was lower (see Table 3, entries 1 and 2). As expected, higher temperatures were generally required for the heterogeneous catalytic reactions due to them having higher activation barriers than the homogeneous procedures. As reported in Table 3 (entry 3), excellent results were observed with the silica-supported catalyst when the reaction was performed at 80 °C for 6 hours in the presence of Si-MonoAm-Cu(I) 5 wt %. Under these conditions, the yield was quantitative, and NMR analysis confirmed the exclusive formation of the desired product. No improvements were observed when the catalyst was loaded at 3 or 9 wt % (Table 3, entries 7–10). For comparison, the reaction was also tested in the absence of both the catalyst and the support, as well as in the presence of bare silica (Table 3, entries 11 and 12). These conditions resulted in 47% to 89% conversion to **2a-o**, respectively. These results highlight the influence of silica in promoting the conversion to the open form **2a-o** and underscore the critical role of copper in catalysing the synthetic process (Table 3, entries 11 and 12). We believe that the exceptional performance of the solid-supported copper catalyst, which was even superior to homogeneous CuCl in terms of product purity and conversion, can be attributed to the heterogeneous nature of the catalyst. Metal–support interactions likely play a critical role in tuning the catalytic behaviour of the supported copper species, further enhancing their efficiency. An additional experiment was conducted using a catalyst prepared by adsorbing CuCl onto silica without the aminopropyl ligand. Under these conditions, only 48% of the product was obtained (Table 3, entry 13). This result demonstrates that the activity of the Cu catalyst is significantly influenced by the surrounding ligands. The amino ligand not only stabilises the catalyst, by ensuring stronger binding to the metal, but also positively influences its reactivity.

**Table 3:** Efficacy of silica-supported Cu(I) and Cu(II) in promoting the C2-amination of benzoxazole with piperidine.

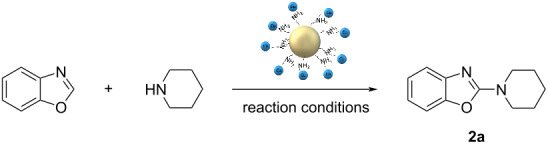				

Entry	Catalyst (20 mol %)^a^	Reaction conditions^b^	Yield^c^	Conversion^c^

1	Si-MonoAm-Cu (I) 5 wt %	60 °C, 6 h	75	>99
2	Si-MonoAm-Cu(II) 5 wt %	60 °C, 6 h	63	89
3	Si-MonoAm-Cu(I) 5 wt %	80 °C, 6 h	98	>99
4	Si-MonoAm-Cu(II) 5 wt %	80 °C, 6 h	81	88
5	Si-DiAm-Cu(I) 5 wt %	80 °C, 6 h	22	89
6	Si-DiAm-Cu(II) 5 wt %	80 °C, 6 h	53	88
7	Si-MonoAm-Cu(I) 3 wt %	80 °C, 6 h	83	98
8	Si-DiAm-Cu(I) 9 wt %	80 °C, 6 h	86	>99
9	Si-DiAm-Cu(II) 3 wt %	80 °C, 6 h	69.2	86
10	Si-DiAm-Cu(II) 9 wt %	80 °C, 6 h	72	92.8
11	silica	80 °C, 6 h	n.d.	89
12	–	80 °C, 6 h	n.d.	47
13	Si CuCl	80 °C, 6 h	48	90
14	Si-MonoAm-Cu(I) 5 wt %	60 °C, 2 h, air 5 bar, MW	67	77
15	Si-MonoAm-Cu(I) 5 wt %	70 °C, 2 h, air 5 bar, MW	71	81
16	Si-MonoAm-Cu(I) 5 wt %	80 °C, 2 h, air 5 bar, MW	99	>99
17	Si-MonoAm-Cu(I) 5 wt %	80 °C, 1,5 h, air 5 bar, MW	99	>99
18	Si-MonoAm-Cu(I) 5 wt %	80 °C, 1 h, air 5 bar, MW	87	95
19	Si-MonoAm-Cu(II) 5 wt %	80 °C, 2 h, air 5 bar, MW	77	90

^a^The loading is theoretical considering a complete reaction of copper salt; ^b^reaction conditions: benzoxazole (0.4 mmol), piperidine (0.8 mmol), copper catalyst theoretical 20 mol %, CH_3_CN (4 mL); ^c^yield and conversion measured by ^1^H NMR.

The influence of MW irradiation on reducing the reaction time was investigated using the SynthWave MW reactor, and reactions were performed under 5 bar of air to avoid the evaporation of the solvent at 80 °C, with this also potentially being beneficial to increasing the amount of dissolved oxygen. As shown in Table 3, MW irradiation effectively accelerated the reaction rate. Complete conversion was achieved at 80 °C within 2 hours using Si-MonoAm-Cu(I). These excellent results, obtained conventionally within 6 hours, were replicated under MW irradiation in less than a third of the time, with the product obtained in pure form without the need for chromatographic purification.

A further reduction in reaction time to 1.5 hours was possible at 80 °C, while incomplete conversion was observed after 1 hour (Table 3, entries 17 and 18). When using Si-MonoAm-Cu(II), the reaction reached 90% conversion and 77% yield after 2 hours of MW irradiation, with the **2a-o** intermediate still present (Table 3, entry 19). This confirms the lower reactivity of the solid-supported Cu(II) salt compared to both the homogeneous catalyst and the supported Cu(I) derivative.

**Scheme 3:**
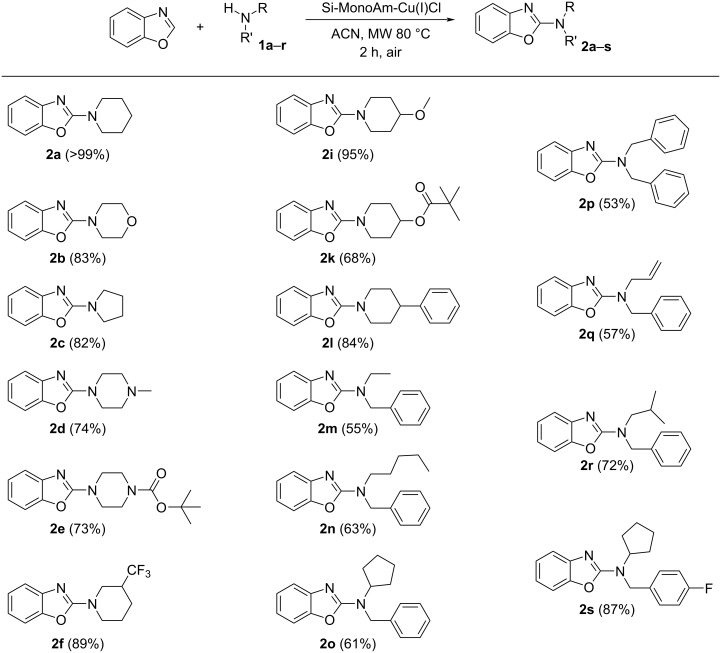
Scope of the MW-promoted C2-amination of benzoxazole catalysed by Si-MonoAm-Cu(I). Reaction conditions: benzoxazole (1.0 mmol), amine (2 mmol), Cu(I) catalyst (0.2 mmol), acetonitrile (1 mL), MW 80 °C, 2 h, 5 bar air; yields refer to isolated compounds.

Next, the scope of the optimised heterogeneous MW-assisted protocol was tested with a set of 16 different secondary amines (Scheme 3). This set included linear, branched aliphatic, and cyclic amines, both commercially available and ad-hoc synthesised derivatives (see Supporting Information File 1 for the syntheses of derivatives **1k**, **1m**–**s**).

Encouragingly, all 16 amines were successfully converted to the corresponding 2-aminobenzoxazole derivatives with yields greater than 50%. Beyond piperidine, other cyclic amines, such as morpholine, piperazine, and pyrrolidine, also reacted efficiently, affording good-to-excellent yields. The Boc-protecting group proved to be stable under the reaction conditions, underlining the importance of avoiding the addition of acidic additives. Steric hindrance exerted a moderate influence on the reaction, and branched aliphatic amines yielded the products **2o**, **2r** and **2s** in the range of 61–87%. The protocol demonstrated compatibility with more lipophilic amines, such as **1n**, and also tolerated amine **1q** containing an allyl substituent.

**Scheme 4:**
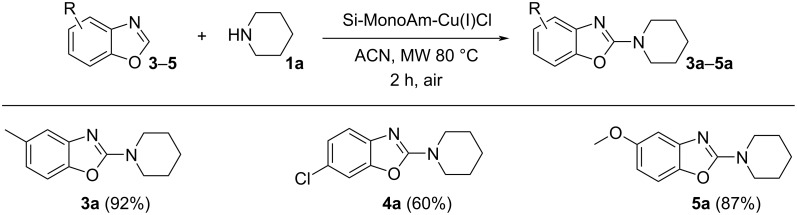
C2-Amination of substituted benzoxazoles. Reaction conditions: benzoxazole (1.0 mmol), piperidine (2 mmol), Cu(I) catalyst (0.2 mmol), acetonitrile (1 mL), MW 80 °C, 2 h, 5 bar air; yields refer to solated compounds.

When the reaction was carried out using substituted benzoxazoles (see Scheme 4), their reactivity with piperidine was confirmed, as observed with the unsubstituted starting material. However, a slight decrease in the yield of 6-chlorobenzoxazole was noted.

**Figure 4:**
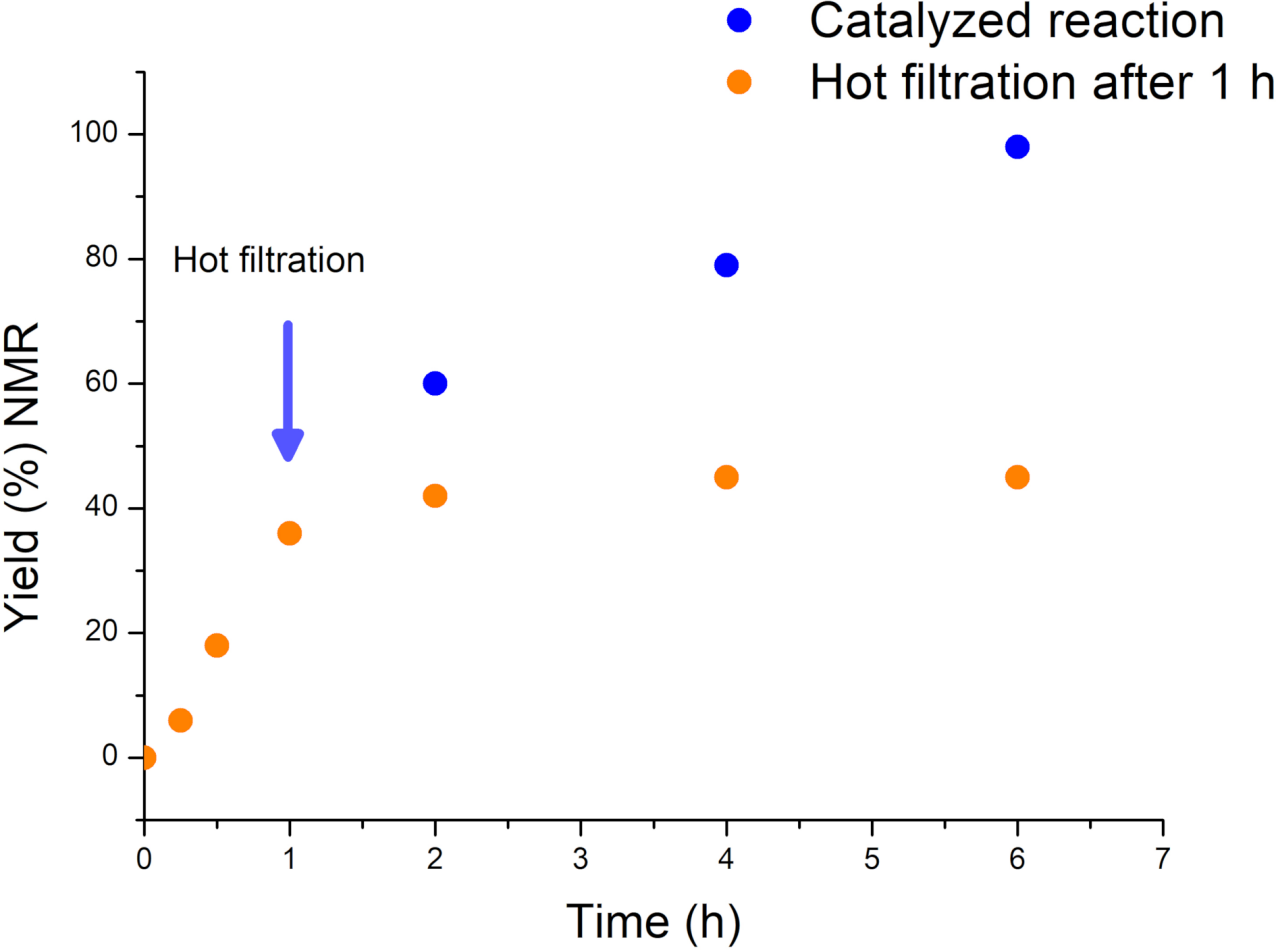
Hot filtration test for the Si-MonoAm-Cu(I)-catalysed C2-amination of benzoxazole with piperidine in acetonitrile at 80 °C. Si-MonoAm-Cu(I) was filtered off after 60 min using a hot filter.

To investigate the heterogeneity of the Si-MonoAm-Cu(I) catalyst, a hot leaching test was carried out. As shown in Figure 4, it was observed that the removal of the solid catalyst after 1 h stopped the reaction. Conversely, when the reaction was allowed to continue in the presence of the catalyst, aerobic oxidation proceeded to completion. This result supports the conclusion that the process operates via true heterogeneous catalysis.

Given the optimal performance of the silica-supported Si-MonoAm-Cu(I) 5 wt % catalyst, we proceeded to measure the amount of copper present using ICP analysis. The catalyst was prepared by reacting silica with CuCl in quantities that, if fully bound to the amino groups, would result in a Cu(I) loading of 5 wt %. The analysis showed that 4.42 ± 0.07 wt % Cu(I) was anchored to the Si-MonoAm support, corresponding to 6.88% CuCl. Similarly, when Si-MonoAm-Cu(II) was analysed, 4.22 ± 0.22 wt % Cu(II) was supported, corresponding to 8.92% CuCl_2_. These results indicate that the lower activity observed with the supported Cu(II) catalyst is not due to a lower amount of copper on the solid surface, but rather to the intrinsic reactivity of Cu(II) itself. ICP analysis was also carried out on all the prepared catalysts to compare their activity. As shown in Table 4, the loading is consistently about 80% of the amount of copper used in the preparation (theoretical loading). This suggests that optimum catalyst distribution and activity is achieved with 5 wt % supported CuCl.

**Table 4:** Copper-supported catalysts.

Entry	Product	Theoretical loading (wt %)^a^	Loading by ICP (wt %)^b^

1	Si-MonoAm-Cu(I)	3	2.47 ± 0.09
2	Si-MonoAm-Cu(I)	5	4.42 ± 0.07
3	Si-DiAm-Cu(I)	3	2.42 ± 0.08
4	Si-DiAm-Cu(I)	5	4.21 ± 0.06
5	Si-DiAm-Cu(I)	9	7.63 ± 0.09
6	Si-MonoAm-Cu(II)	5	4.22 ± 0.22
7	Si-DiAm-Cu(II)	5	4.09 ± 0.04
8	Si-DiAm-Cu(II)	9	7.01 ± 0.07

^a^The theoretical loading considers a complete reaction of Cu(I or II) chloride with amino silica derivatives and it refers to wt % Cu (see Table S1 in Supporting Information File 1 for reaction conditions); ^b^the loading is measured by ICP analysis and it refers to wt % of Cu.

The two catalysts were also characterised by FTIR and DR UV–vis spectroscopy, to obtain information about the grafted aminopropyl ligand and inserted copper functionality, respectively.

**Figure 5:**
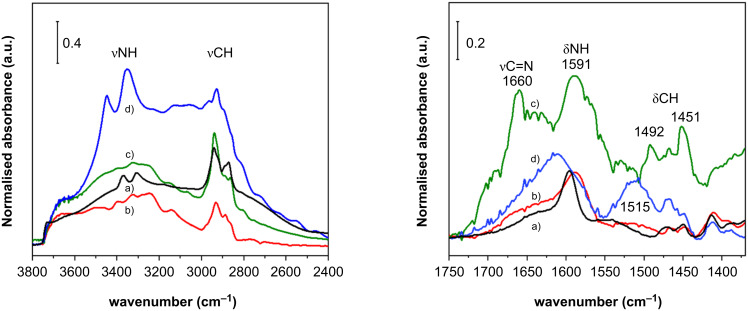
FTIR spectra of samples on the left 3800–2400 cm^−1^ wavenumber on the right 1750–1350 cm^−1^ wavenumber; a) Si-MomoAm, b) 5% Si-MonoAm-Cu(I), c) Si-MonoAm-Cu-used and d) 5% Si-MomoAm-Cu(II).

Figure 5 shows the FTIR spectra measured on the silica support functionalised with the aminopropyl group, and the subsequent insertion of copper. The successful grafting of the amino ligand is testified by the characteristic stretching and bending modes of the NH_2_ group (νNH_2_ and δNH_2_, at around 3370, 3300 and 1596 cm^−1^, respectively) and by the νCH_2_ and δCH_2_ vibrations (curve a) [64–65]. The broad absorption in the high frequency region is characteristic of hydrogen bonding between Si–OH and NH_2_ groups in functionalised silica materials. After insertion of Cu(I), small changes in the position and shape of the νNH_2_ and δNH_2_ bands are observed, which are an indirect indication of the interaction of the amino group with the metal (curve b) [51]. On the other hand, major changes are observed in the spectrum of 5 wt % Si –MonoAm-Cu(II) (curve d). These are particularly informative in the low frequency range, where two new broad bands are observed at 1610 and 1515 cm^−1^, which are compatible with the presence of –NH_3_^+^ groups (antisymmetric and symmetric bending modes, respectively) [66–67].

The study also aimed to characterise the catalyst Si-MonoAm-Cu(I) after usage in optimised conditions (80 °C, MW, 2 h) with both techniques (curves c in Figure 5). The fingerprints of the aminopropyl groups are still observable in the FTIR spectrum, but new bands are formed at 1660 cm^−1^ and below 1500 cm^−1^, with a minor component around 1700 cm^−1^, which could be related to the adsorption of an imino derivative, and, more specifically, νC=N (1660 with shoulder around 1700 cm^−1^) and δN–H (1590 cm^−1^, overlapping with the δNH_2_ of the aminopropyl groups) in –C=N–H (Figure 5). According to the literature, copper species may contribute to the partial oxidation of amino groups to imino groupds [68], while still retaining the ability of a Schiff base to efficiently bind transition metals, including copper [69].

An advantage of using a heterogeneous catalyst, beyond the easy filtration from the reaction mixture, lies in its possible reuse, multiple times, directly or after performing regeneration. Thus the exhausted Si-MonoAm-Cu(I) catalyst was therefore reused, but, unfortunately, reduced efficiency and selectivity were detected (85% conversion and 60% yield).

For this reason, catalyst regeneration was performed by suspending the catalyst in a solution containing CuCl in THF. Based on optimisation, we observed that the amount of CuCl required for regeneration was lower than in the preparation step; the addition of only 50% of the CuCl amount used for the preparation of the fresh catalyst was required to achieve comparable performance.

**Figure 6:**
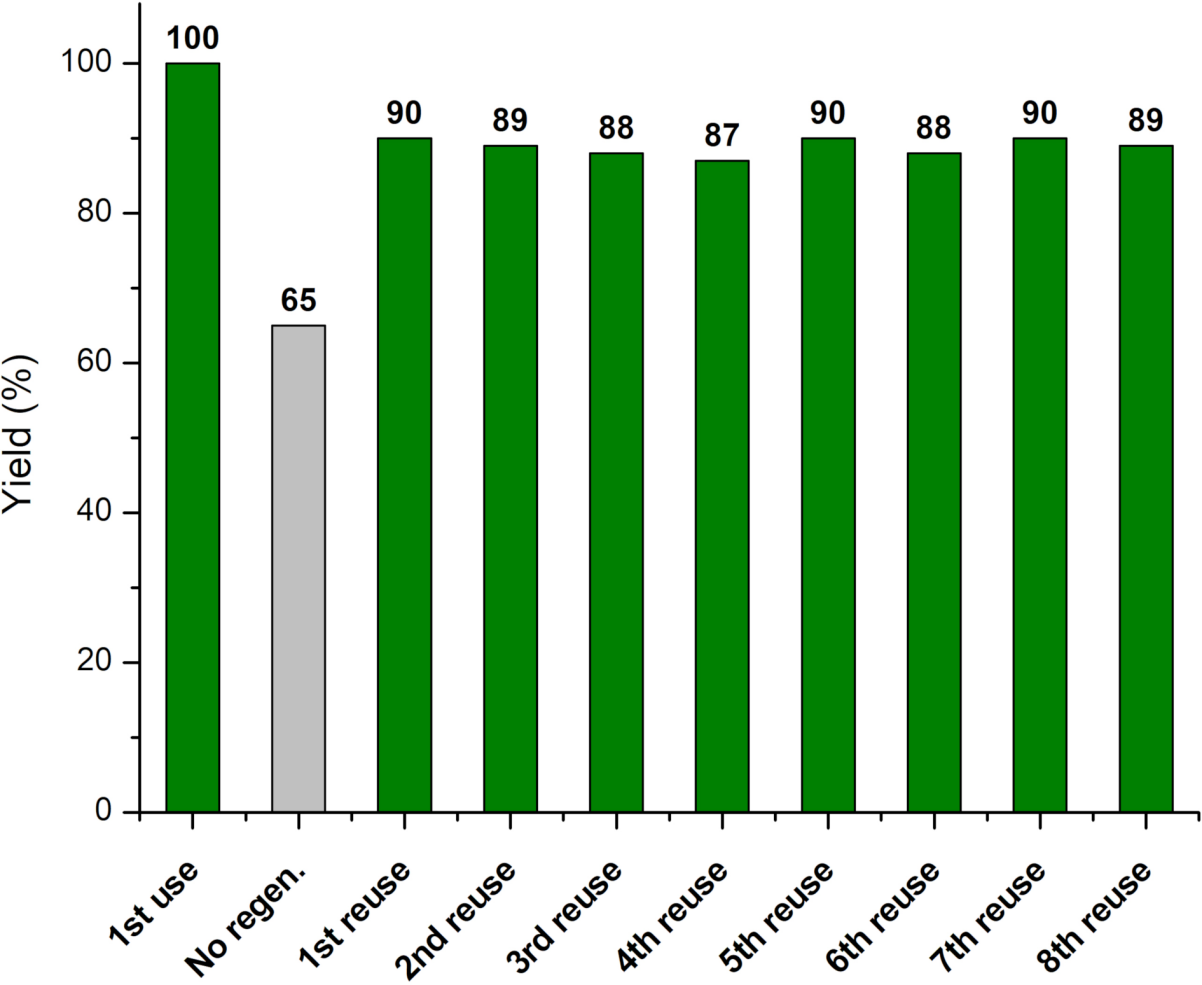
Si-MonoAm-Cu(I) catalyst reuse.

The regenerated catalyst was then used in the optimised microwave conditions, which confirmed that its selectivity to the desired product had been restored, as reported in Figure 6. Reuse was performed eight times without observing consistent catalytic deactivation.

FESEM images of Si-MonoAm-Cu(I) before and after usage were also acquired to demonstrate the dispersion of Cu and possible alterations of morphology.

**Figure 7:**
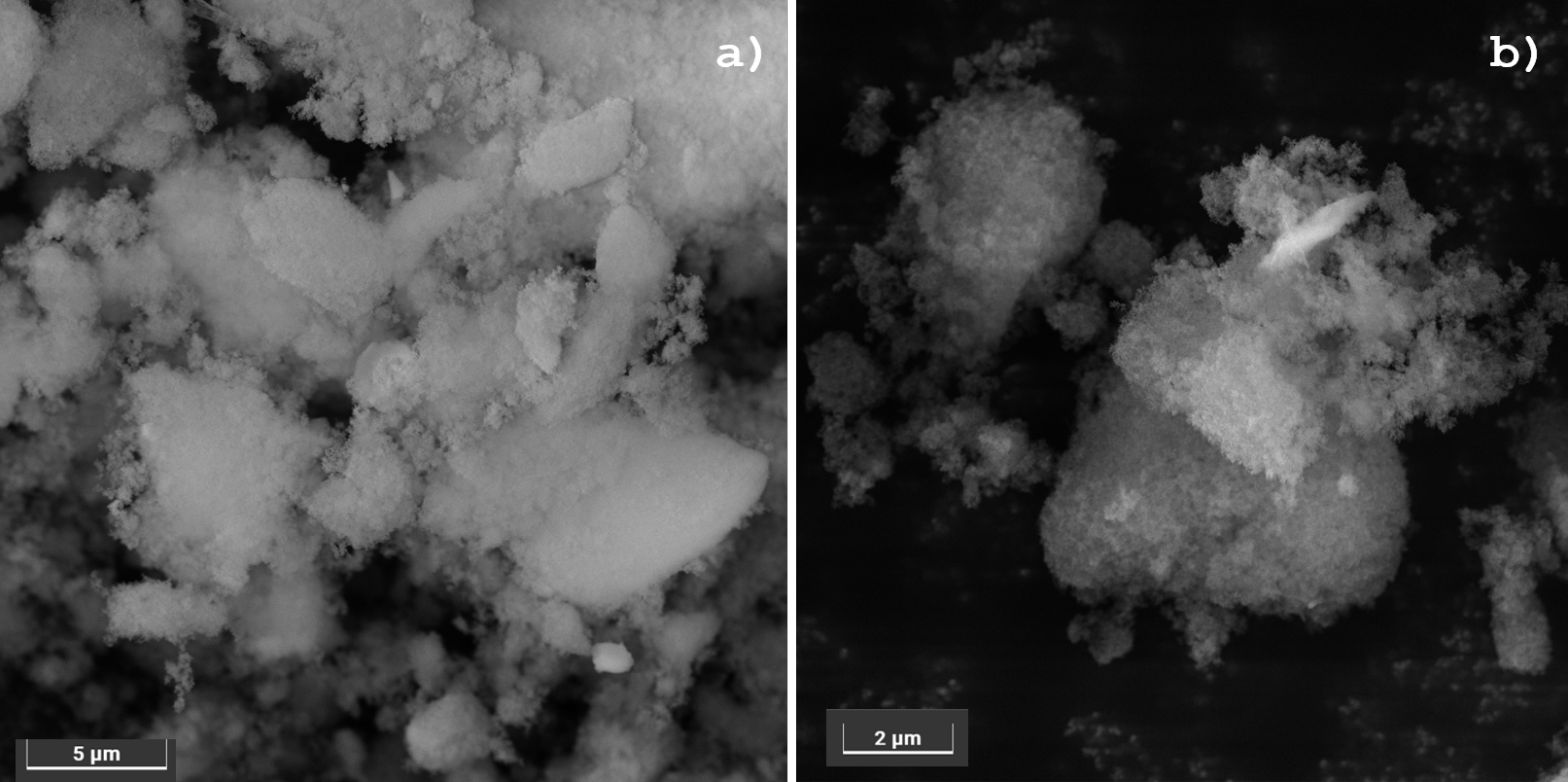
FESEM images of sample a) Si-MonoAm-Cu(I) 5 wt % and c) Si-MonoAm-Cu(I) 5 wt % used.

**Figure 8:**
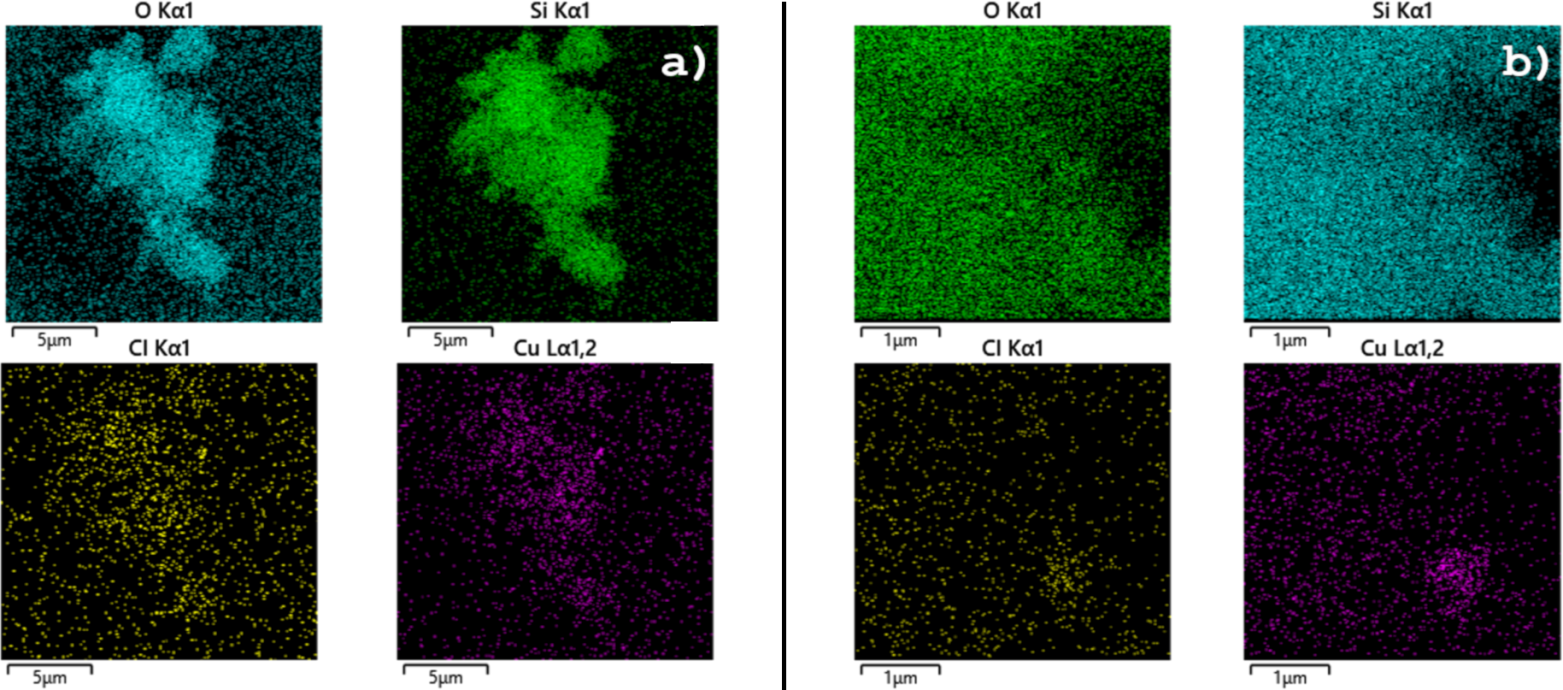
EDS maps of a) Si-MonoAm-Cu(I) and b) Si-MonoAm-Cu(I) used.

As reported in Figure 7, the silica support is characterised by irregular micrometre-sized agglomerates of nanometre-sized particles. This morphology is not modified after the use of the Si-MonoAm-Cu(I) catalyst in the reaction, as can be seen by comparing the corresponding FESEM images of the fresh and used catalyst (see Figure 7a and b). EDS maps show a homogeneous dispersion of copper on the catalyst with this dispersion being maintained after the catalysts is used (panels a and b of Figure 8).

## Conclusion

The study demonstrates that the efficient and green direct C–H amination of benzoxazoles can be catalysed by copper chlorine salts in acetonitrile in the absence of any acidic, basic, or oxidising additives. Both CuCl and CuCl_2_ have been found to be highly efficient in promoting the reaction, on the base of their activity as Lewis acids and weak oxidants. MW irradiation has led to a significant enhancement in reaction rate with the reaction running to completion in only 1.5–2 h.

The derivatisation of silica with pendent primary amino groups has granted it the capability to stably support copper(I) and (II) in a cheap and efficient MW-promoted procedure that has been optimised with solid-supported Cu(I). The heterogeneous process dramatically simplified the work-up of the reaction, allowing the catalyst to be removed from the reaction mixture via simple filtration. Moreover, it led to an increase in the amount of recovered products and to a decrease in the amount of free copper in solution. Catalyst reuse has been explored, and it has been confirmed that reuse up to eight times is possible after proper regeneration. A wide library of derivatives has been easily synthesised with the optimised procedure by reacting benzoxazole and its 5- or 6-substituted derivatives with a variety of cyclic, alkyl and branched secondary amines, probing the protocol’s versatility and confirming its potential usefulness. This study provides the basis for the application of the protocol in continuous flow systems, such as a continuous heterogeneous reactor known as a packed bed reactor, enabling the production of 2-aminobenzoxazoles at scales ranging from large laboratory batches to pilot scale operations.

## Experimental

### Materials and methods

All chemicals were purchased from Sigma-Aldrich (Milan, Italy) and used without further purification. SIPERNAT 320 amorphous silica was supplied by Evonik. Reactions were monitored by TLC on Merck 60 F254 (0.25 mm) plates (Milan, Italy), which were visualised by UV inspection and/or by heating after spraying with 0.5% ninhydrin in ethanol or phosphomolybdic acid. Homogeneously catalysed reactions were performed in a professional MW oven (MicroSynth MLS GmbH, Milestone S.r.l.), while heterogeneously catalysed reactions were carried out in a professional MW reactor SynthWave (MLS GmbH, Milestone S.r.l.). The SynthWave MW cavity was filled with 5 bar of air.

The syntheses of amino compounds **1k** and **1m**–**s** and of substituted benzoxazoles **3**–**5** and characterisation of products and solid-supported copper catalysts are reported in Supporting Information File 1.

#### General procedure for the preparation and regeneration of Si-MonoAm-Cu(I) 5 wt %

Silica SIPERNAT 320 (1 g) was added to a solution of 3-aminopropyltriethoxysilane (2 mmol) in toluene (10 mL). The suspension was sonicated for 2 h in an US bath (Power 200 W, Frequency 80 kHz). The resulting silica was then filtered, washed with toluene and chloroform, and dried under vacuum at room temperature for 12 hours. The derivatised silica was characterised by means of TGA and FTIR.

Si-MonoAm (200 mg) and 16 mg of Cu(I)Cl were dispersed in 4 mL of THF. The mixture was stirred at room temperature for 4 hours, filtered under reduced pressure, and the powder was washed with THF and CHCl_3_. The resulting product was then kept in a desiccator overnight and fully characterised.

When undergoing regeneration, 200 mg of the exhausted Si-MonoAm-Cu(I) and 8 mg of CuCl were dispersed in 4 mL of THF. The reaction mixture was treated as described for the preparation of the fresh catalyst.

#### General procedure for synthesis of derivative **2a** by means of homogeneous catalysis

Benzoxazole (0.4 mmol), piperidine (0.63–0.8 mmol), and Cu catalyst (CuCl or CuCl_2_·2H_2_O 0.08 mmol, 20 mol %) were dissolved in CH_3_CN (4 mL). The reaction mixture was heated to 60–80 °C for 6 h, after which the solvent was evaporated under reduced pressure. The resulting residue was dissolved in 10 mL of CHCl_3_, and the organic phase was extracted with 3.5 M aqueous ammonia solution (1 × 10 mL) and distilled water (2 × 10 mL). The organic phase was washed with brine, dried over sodium sulphate, and filtered. The solvent was removed under reduced pressure to obtain a pure product. Where required, the residue was purified by flash chromatography over basic alumina, using a PE/EtOAc 7:3 mixture as the eluent.

When the reaction was performed in a MW oven, the MicroSynth instrument (Milestone) was used. The reaction was performed in an open round-bottomed flask that was heated to 60 °C for 2 h (see Supporting Information File 1 for details on MW procedure setup).

#### Synthesis of derivatives **2a–s**, **3a**, **4a**, **5a** with Si-MonoAm-Cu(I)

Benzoxazole (0.4 mmol), amine (0.8 mmol) and Si-MonoAm-Cu(I) 5 wt % (100 mg, 0.08 mmol, 20 mol %) were dispersed in CH_3_CN (4 mL). The reaction mixture was heated in an oil bath to 80 °C for 6 h, and the catalyst was then removed by filtration and recovered where required. The solvent was evaporated under reduced pressure. The resulting residue was dissolved in 10 mL of CHCl_3_, and the organic phase was extracted with distilled water (2 × 10 mL). The organic phase was washed with brine, dried over sodium sulphate and filtered. The solvent was removed under reduced pressure. When required, the residue was purified by flash chromatography over basic alumina, using a PE/EtOAc 7:3 mixture as the eluent.

When performed under MW irradiation, the reaction was heated at 80 °C for 2 h. Before the reaction started, the reactor was pressurized with 5 bar of air (for experimental MW setup see Supporting Information File 1).

## Supporting Information

File 1 Experimental procedures, compound characterization data, and copies of NMR spectra.

## Data Availability

All data that supports the findings of this study is available in the published article and/or the supporting information of this article.
