# Supplementary material for: Microwave-enhanced additive-free C–H amination of benzoxazoles catalysed by supported copper
Source: Beilstein J Org Chem. 2025 Jul 15;21:1462–76. doi: 10.3762/bjoc.21.108 (PMC12278112; doi:10.3762/bjoc.21.108)
Supplement: File 1 — Experimental procedures, compound characterization data, and copies of NMR spectra. [file Beilstein_J_Org_Chem-21-1462-s001.pdf]

BEILSTEIN JOURNAL OF ORGANIC CHEMISTRY

Supporting Information

for

Microwave-enhanced additive-free C–H amination of benzoxazoles catalysed by supported copper

Andrei Paraschiv, Valentina Maruzzo, Filippo Pettazzi, Stefano Magliocco, Paolo Inaudi, Daria Brambilla, Gloria Berlier, Giancarlo Cravotto and Katia Martina

Beilstein J. Org. Chem. **2025,** *21,* 1462–1476. doi:10.3762/bjoc.21.108

Experimental procedures, compound characterization data, and copies of NMR spectra

License and Terms: This is a supporting information file under the terms of the Creative Commons Attribution License (https://creativecommons.org/licenses/by/4.0). Please note that the reuse, redistribution and reproduction in particular requires that the author(s) and source are credited and that individual graphics may be subject to special legal provisions.
The license is subject to the *Beilstein Journal of Organic Chemistry* terms and conditions: (https://www.beilstein-journals.org/bjoc/terms)

Table of contents

General information S1

Synthetic procedures S2

Synthetic scheme for catalyst preparation S2

General procedure for the synthesis of derivative **2a** by means of homogeneous catalysis S4

MW-promoted synthesis of derivatives **2a–s** and **3–5a** with Si-MonoAm-Cu(I) S4

Synthesis of 2,2-dimethylpropionic acid piperidin-4-yl ester (**1k**) S7

General procedure for the synthesis of *N*-substituted benzylamines **1m–s** S9

Synthesis of substituted benzoxazoles **3–5** S11

Characterization of derivatives **2a** open form **2a**-o, **2a–s** and **3a**, **4a**, **5a** S12

NMR spectra S18

MS spectra S48

References S60

General information

All chemicals were purchased from Sigma-Aldrich (Milan, Italy) and used without further purification. Reactions were monitored by TLC on Merck 60 F254 (0.25 mm) plates (Milan, Italy), which were visualized by UV inspection and/or by heating after a spraying with 0.5% ninhydrin in ethanol or phosphomolybdic acid. Homogeneously catalyzed reactions were performed in a professional MW oven MicroSynth (MLS GmbH, Milestone S.r.l.), while heterogeneously catalyzed reaction were carried out in a professional MW reactor SynthWave (MLS GmbH, Milestone S.r.l.). US irradiation at 80 kHz was performed in highly efficient bath reactors supplied by Weber Ultrasonics GmbH (Karlsbad-Ittersbach, Germany).

^{1}H and ^{13}C NMR spectra were recorded on a JEOL ECZR600 instrument. Chemical shifts were calibrated to the residual proton and carbon resonances of the solvent $CDCl_3$ (δH = 7.26, δC = 77.16). Chemical shifts (δ) are given in ppm and coupling constants (*J*) in Hz.

GC–MS analyses were performed using a GC Agilent 6890 (Agilent Technologies, Santa Clara, CA, USA), fitted with a mass detector Agilent Network 5973, and a 30 m capillary column, i.d. of 0.25 mm and film thickness 0.25 μm (Agilent 19091S-433E) HRMS was determined using a Zeno TOF 7600 System.

Thermogravimetric analyses were performed using a thermogravimetric analyzer TGA 4000 (PerkinElmer) at 10 °C min^{-1} operating with alumina crucibles that contained 10–20 mg of sample. The analyses were performed under a nitrogen atmosphere at a starting temperature of 50 °C and an end temperature of 800 °C.

Infrared spectra were recorded on an Equinox 55 Bruker FTIR spectrophotometer with a resolution of 2 cm^{-1}, using an MCT detector. Measurements were carried out using a home-made cell allowing in situ thermal treatment and room temperature measurement. Thin self-supporting pellets for transmission measurements (around 10 mg/cm^{2}) were prepared with a hydraulic press. Before the measurements, the samples were outgassed at 80 °C for 1 h in the same cell used for the measurements.

The images of the materials were acquired using an FEG-SEM S9000 by Tescan equipped with EDS for microanalysis. The measurements were carried out with a Schottky emitter, working at 15 keV with a probe current set at 100 pA. The analyses were carried out with an in-beam secondary electron detector.

The cations were determined with a Perkin Elmer Optima 7000 (Perkin Elmer, Norwalk, Connecticut, USA) inductively coupled plasma-optical emission spectrometer (ICP-OES).

Synthetic procedures

Synthetic scheme for catalyst preparation

Preparation of Si-MonoAm

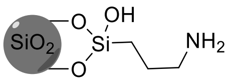

The procedure was performed in a manner analogous to [1].

3-Aminopropyltriethoxysilane (0.424 mL) was dispersed in toluene (10 mL), and silica SIPERNAT 320 (1 g) was added to the mixture. The suspension was sonicated 2 h in a US bath reactor (power 200 W, frequency 80 kHz). Silica was filtered, washed with toluene and chloroform, and dried under a vacuum at room temperature for 12 h.

Preparation of Si-DiAm

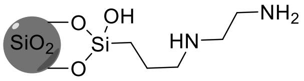

The procedure was performed in a manner analogous to [1].

3-(2-Aminoethylamino)propyltrimethoxysilane (0.392 mL) was dispersed in toluene (10 mL), and silica SIPERNAT 320 (1 g) was added to the mixture. The suspension was sonicated 2 h in US bath (power 200 W, frequency 80 kHz). Silica was filtered, washed with toluene and chloroform, and dried under a vacuum at room temperature for 12 h.

Preparation of silica-supported copper Si-MonoAm-Cu(I or II) and Si-DiAm-Cu(I or II)

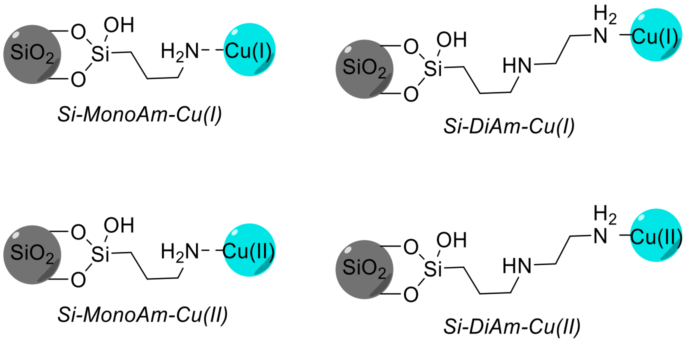

The procedure was performed in a manner similar to [2].

In a round-bottomed flask, Si-MonoAm and Si-DiAm (200 mg) and CuCl or $CuCl_2{\cdot}2H_2O$ (0.09 mmol or 0.159 mmol or 0.285 mmol, respectively to obtain 3 or 5 or 9 wt %) were dispersed in 4 mL of THF. The mixture was stirred at room temperature for 6 hours, followed by filtration under reduced pressure. The resulting solid was thoroughly washed with THF and $CHCl_3$, then stored in a desiccator for 12 h to ensure complete drying (see Table S1 for details on the prepared supported catalyst).

Regeneration of Si-MonoAm-Cu(I)

In a round-bottomed flask, exhausted Si-MonoAm-Cu 5% (200 mg) and CuCl (8 mg, 0.079 mmol) were dispersed in 4 mL of THF. The mixture was stirred at room temperature for 6 hours, followed by filtration under reduced pressure. The resulting solid was thoroughly washed with THF and $CHCl_3$, then stored in a desiccator for 12 h to ensure complete drying.

Table S1: Copper-supported catalysts.

Entry	Product	Linker	Loading[a] [w/w%]	Loading [umol/g]	Loading ICP[e] [w/w%]
1	Si-MonoAm-Cu(I)	$-(CH_2)_3NH_2$	3[b]	555	2.47 ± 0.09
2	Si-MonoAm-Cu(I)	$-(CH_2)_3NH_2$	5[c]	793	4.42 ± 0.07
3	Si-DiAm-Cu(I)	$-(CH_2)_3NH(CH_2)_2NH_{2-}$	3[b]	555	2.42 ± 0.08
4	Si-DiAm-Cu(I)	$-(CH_2)_3NH(CH_2)_2NH_{2-}$	5[c]	793	4.21 ± 0.06
5	Si-DiAm-Cu(I)	$-(CH_2)_3NH(CH_2)_2NH_{2-}$	9[d]	1428	7.63 ± 0.09
6	Si-MonoAm-Cu(II)	$-(CH_2)_3NH_2$	5[c]	793	4.22 ± 0.22
7	Si-DiAm-Cu(II)	$-(CH_2)_3NH(CH_2)_2NH_{2-}$	5[c]	793	4.09 ± 0.04
8	Si-DiAm-Cu(II)	$-(CH_2)_3NH(CH_2)_2NH_2$	9[d]	1428	7.01 ± 0.07

Reaction conditions: a) the loading is theoretical considering a complete reaction of copper salt and it refers to weight Cu %; b) CuCl (9.4 mg) Si-MonoAm or Si-DiAm (200 mg), THF 4 mL, rt, 6 h c) CuCl or $CuCl_2·2H_2O$ (16 mg or 27 mg, respectively) Si-MonoAm or Si-DiAm (200 mg) THF 4 mL, rt, 6 h, d) CuCl or $CuCl_2·2H_2O$ (28 mg or 48 mg, respectively) Si-MonoAm or Si-DiAm (200 mg mg) THF 4 mL, rt, 6 h e) the loading is measured by ICP analysis and it refers to wt % Cu.

General procedure for the synthesis of derivative 2a by means of homogeneous catalysis

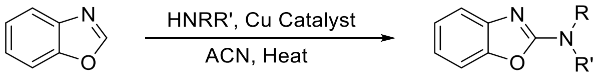

Benzoxazole (0.4 mmol), piperidine (0.63–0.8 mmol), and Cu catalyst (0.08 mmol, 20 mol %) were dissolved in CH_3CN (4 mL). The reaction mixture was heated to 80–60 °C for 6 hours, after which the solvent was evaporated under reduced pressure. The resulting residue was dissolved in 10 mL of $CHCl_3$, and the organic phase was extracted with 3.5 M aqueous ammonia solution (1 × 10 mL) and distilled water (2 × 10 mL). The organic phase was washed with brine, dried over sodium sulfate, and filtered. The solvent was removed under reduced pressure to obtain a pure product. If required the residue was purified by flash chromatography on basic alumina, using a PE/EtOAc 7:3 as the eluent.

When the reaction was performed in a MW oven the MicroSynth instrument (Milestone) was used. The reaction was performed in an opened round-bottomed flask and it was heated to 60 °C for 2 h.

The instrumental procedure was setup as in the following:

1- Heat to 60 °C in 1 min (max power 800 W)
2- Maintain the temperature at 60 °C for 2 hours (max power 200 W)
3- Allow the reaction mixture to cool to room temperature

See profile in Figure S2

MW-promoted synthesis of derivatives 2a–s and 3–5a with Si-MonoAm-Cu(I)

Benzoxazole (0.4 mmol), amine (0.8 mmol), and Si-MonoAm-Cu(I) 5 wt % (100 mg, 0.08 mmol, 20 mol %) were dissolved in CH_3CN (4 mL). The reaction mixture was heated under MW irradiation in the MW Synthwave reactor to 80 °C for 2 h, before the reaction started the reactor was loaded with 5 bar of air. The catalyst was removed by filtration and if required the catalyst was recovered. The solvent was therefore evaporated under reduced pressure. The resulting residue was dissolved in 10 mL of $CHCl_3$, and the organic phase was extracted with distilled water (2 × 10 mL). The organic phases were washed with brine, dried over sodium sulfate, and filtered. The solvent was removed under reduced pressure. When requested the residue was purified by flash chromatography on basic alumina, using a Pe/EtOAc mixture (7:3) as the eluent.

Synthwave setup: The reactor was pressurized with 5 bar of air the instrumental procedure employed was as outlined below:

1- Heat to 80 °C in 3.5 min (Max Power 1500 W)
2- Maintain the temperature to 80 °C for 2 hours (Max Power 1500 W)
3- Allow the reaction mixture to cool to room temperature and release the pressure

See profile in Figure S3

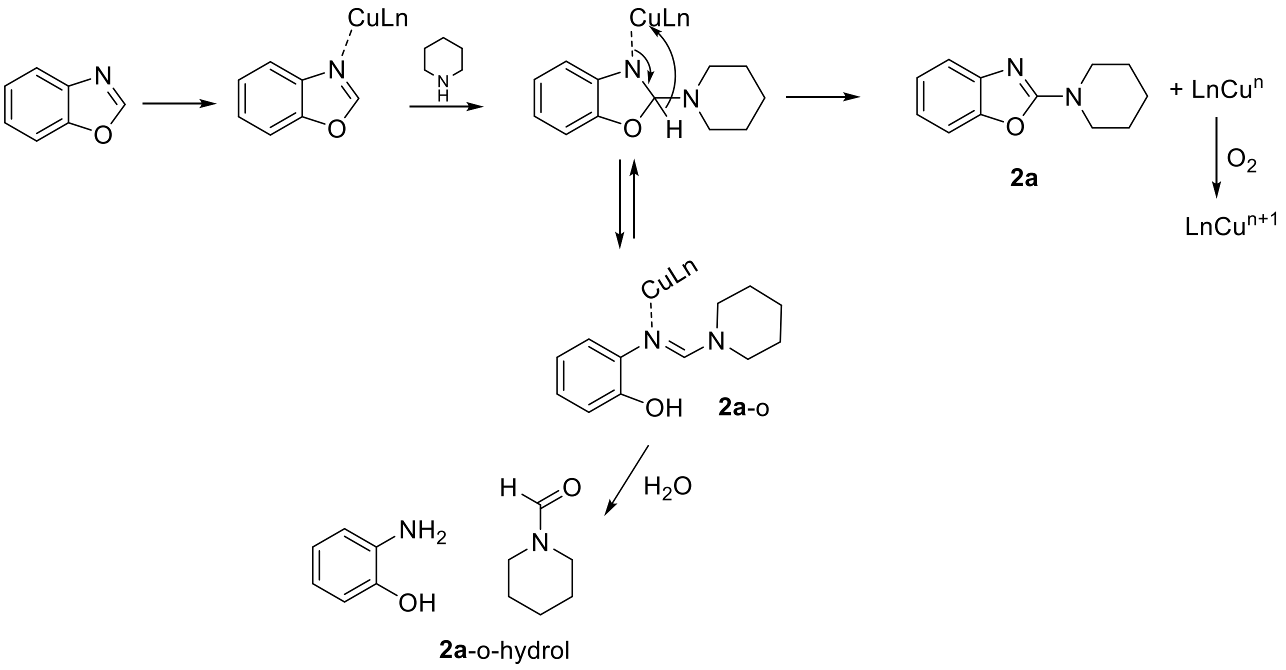

Scheme S1 Possible mechanism for copper-catalyzed direct C–H amination of benzoxazole with piperidine.

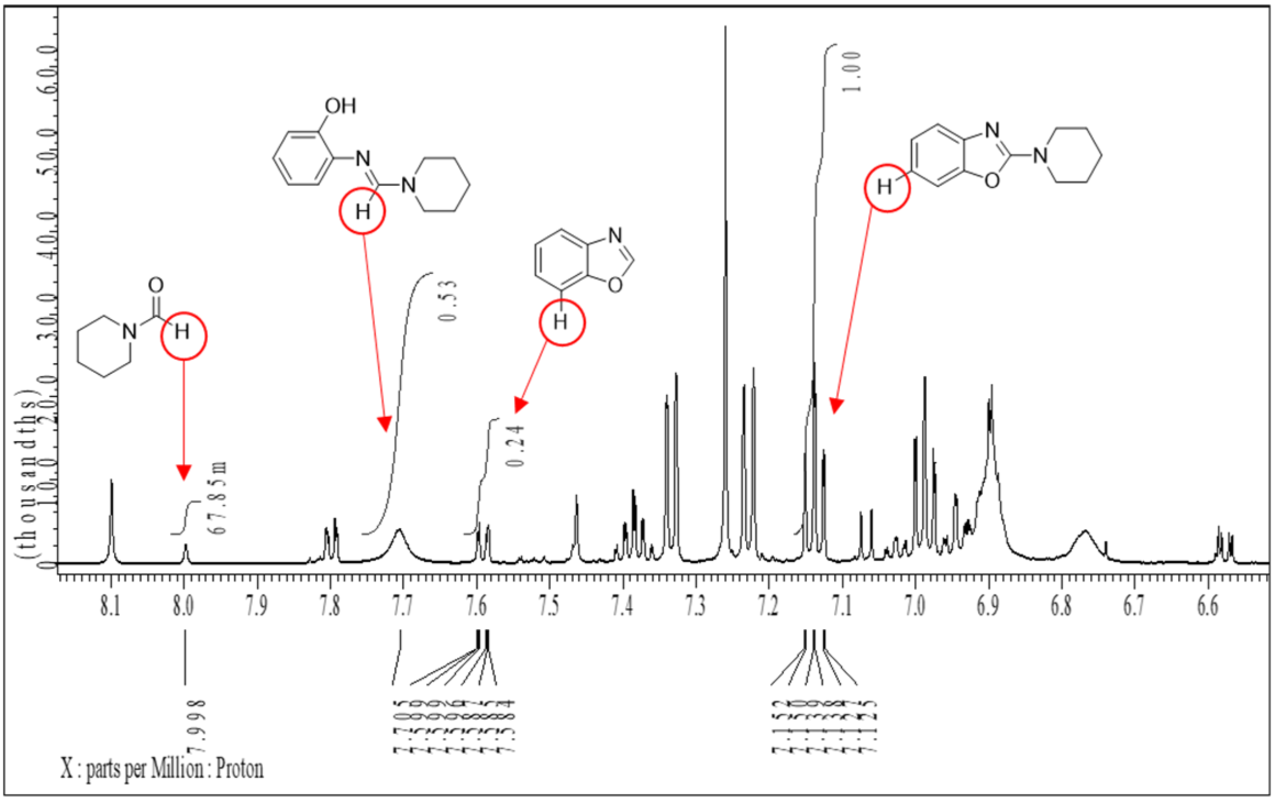

Figure S1 Explanatory ^{1}H NMR spectrum of the crude reaction mixture in deuterated chloroform of the CH amination of benzoxazole. The reported experiment corresponds to the crude reaction Table 3, entry 6.

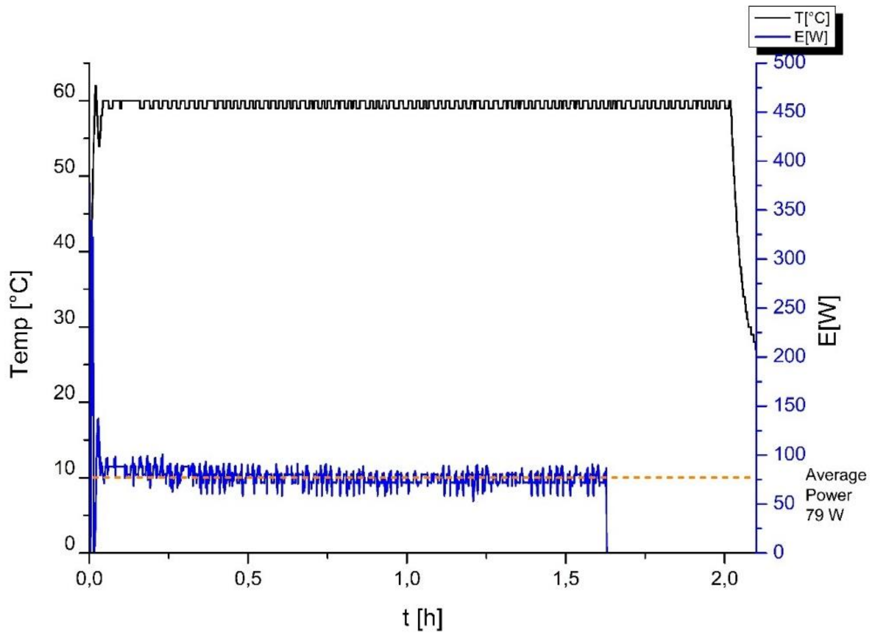

Figure S2 MW-promoted protocol using a Microsynth Microwave Reactor. Temperature and power profile curves registered Program: P_{max} = 800 W, 1 min to reach 60 °C, then *T* = 60 °C for 2 h.

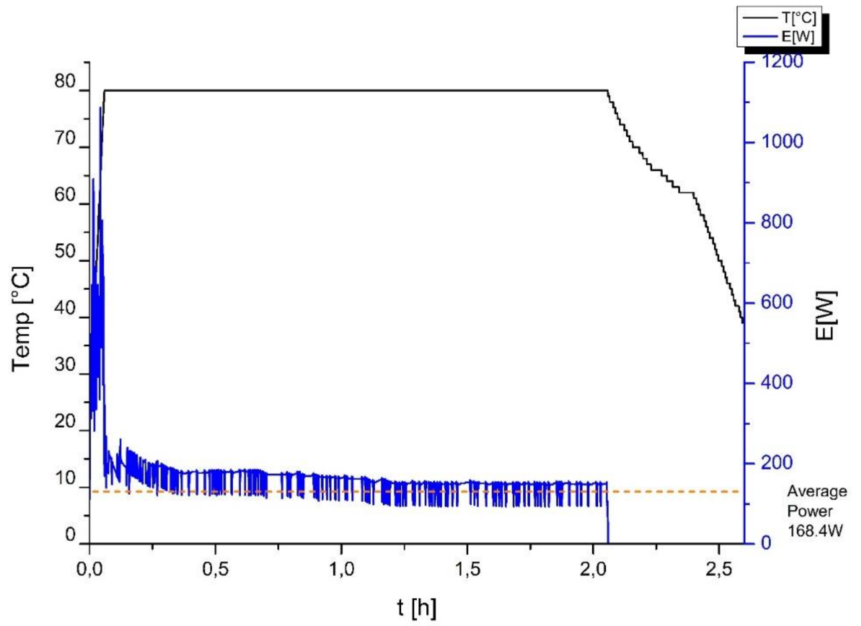

Figure S3 MW-promoted protocol using a Synthwave Microwave Reactor. Temperature and power profile curves registered Program: P_{max} = 1500 W, 3.5 min to reach 80 °C, then *T* = 80 °C for 2 h.

Synthesis of 2,2-dimethylpropionic acid piperidin-4-yl ester (1k)

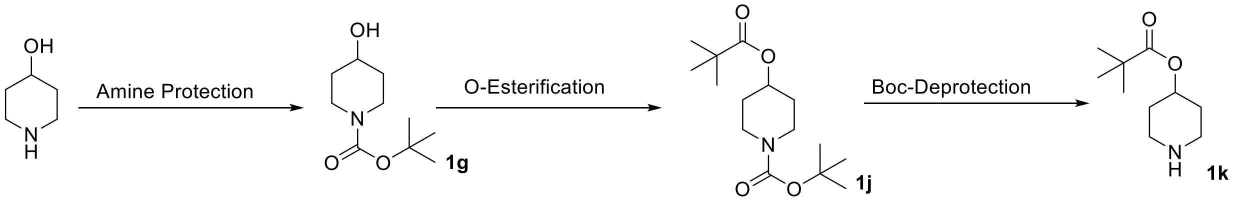

*Synthesis of tert-butyl 4-hydroxy piperidine-1-carboxylate (**1g**)*

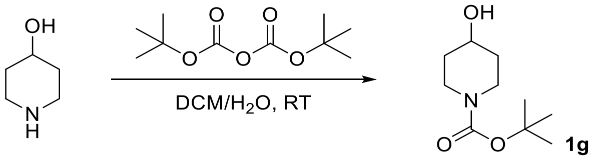

In a round-bottomed flask, 4-hydroxypiperidine (14.8 mmol) was dissolved in CH_2Cl_2 (41 mL). Di-*tert*-butyl dicarbonate (8.2 mmol) and a saturated aqueous solution of sodium carbonate (60 mmol, 50 mL) were then added. The biphasic mixture was stirred at room temperature and monitored by thin-layer chromatography (TLC, PE:EtOAc 5:5) until the reaction reached completion. Subsequently, the organic layer was separated, and the aqueous phase was extracted three times with CH_2Cl_2 (3 × 20 mL). The combined organic layers were washed with brine and dried over sodium sulfate. The final product, a white solid, was obtained by filtering off the sodium sulfate and removing the solvent under reduced pressure, yield 100% (14.8 mmol, 2.98 g).

^{1}H NMR (600 MHz, $CDCl_3$) δ 3.80-3.76 (m, 3H), 2.98 (t, J = 10.5 Hz, 2H), 2.34 (s, 1H), 1.81 (d, J = 12.4 Hz, 2H), 1.45-1.39 (m, 11H)

The characterization data are in agreement with those reported in the literature. [3]

*Synthesis of tert-butyl 4-(2,2-dimethylpropanoyloxy)piperidine-1-carboxylate (**1j**)*

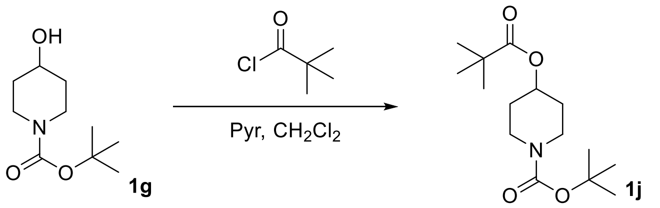

tert-Butyl 4-hydroxypiperidine-1-carboxylate (5 mmol) was dissolved in 50 mL of CH_2Cl_2 in a round-bottomed flask. Pyridine (14 mmol) was then added, followed by the dropwise addition of 2,2-dimethylpropanoyl chloride. The reaction mixture was stirred at room temperature and monitored by thin-layer chromatography (TLC, PE:EtOAc 5:5) until complete conversion was achieved. Upon completion, the solvent was removed under reduced pressure, and the crude product was dissolved in 20 mL of water. The aqueous phase was extracted with EtOAc (3 × 10 mL). The pure product was subsequently isolated by column chromatography (PE:EtOAc as the eluent), yielding a yellow crystalline solid with a quantitative yield of 100% (5 mmol, 1.42 g).

^{1}H NMR (600 MHz, $CDCl_3$) δ 4.93-4.90 (m, 1H), 3.58 (t, J = 8.6 Hz, 2H), 3.37-3.33 (m, 2H), 1.80 (td, J = 8.4, 3.9 Hz, 2H), 1.61 (d, J = 6.2 Hz, 2H), 1.46 (s, 9H), 1.19 (s, 9H)

^{13}C NMR (151 MHz, $CDCl_3$) δ 177.8, 154.7, 79.6, 68.9, 38.8, 30.3, 28.4, 27.1, 27.0

HR-ESI-MS m/z calcd. for $C_{15}H_{28}NO_4$ $[M+H]^+$: 286,2013, found: 286,2012; 230,1385 $[M-C_4H_9+2H]^+$, 186,1487 $[M-C_5H_9O_2+2H]^+$

*Synthesis of 2,2-dimethylpropionic acid piperidin-4-yl ester (**1k**)*

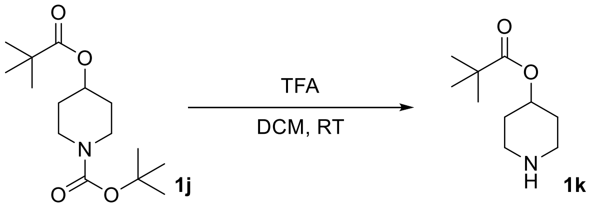

tert-Butyl 4-(2,2-dimethylpropanoyloxy)piperidine-1-carboxylate (5 mmol) was dissolved in 65 mL of CH_2Cl_2 in a round-bottomed flask. Trifluoroacetic acid (10 mmol) was then added dropwise, and the reaction mixture was stirred at room temperature, with progress monitored by thin-layer chromatography (TLC, DCM:MeOH 7:3). After the reaction was completed, the solvent was removed under reduced pressure, and the crude product was dissolved in 30 mL of 3% ammonia solution. The aqueous phase was extracted with $CHCl_3$ (5 × 20 mL). The combined organic phases were washed with brine, dried over sodium sulfate, and filtered. The solvent was removed under reduced pressure, and the resulting product was obtained as a yellow solid with a 60% yield (3.5 mmol, 650 mg).

^{1}H NMR (600 MHz, $CDCl_3$) δ 4.81-4.77 (m, 1H), 3.01-2.98 (m, 2H), 2.71-2.67 (m, 2H), 1.81 (tt, J = 9.5, 3.3 Hz, 3H), 1.51 (tt, J = 12.7, 4.4 Hz, 2H), 1.14 (s, 9H)

^{13}C NMR (151 MHz, $CDCl_3$) δ 177.7, 69.8, 43.6, 38.6, 31.8, 27.0

HR-ESI-MS m/z calcd. for $C_{10}H_{20}NO_2$ $[M+H]^+$: 186,1489, found: 186,1489

General procedure for the synthesis of *N*-substituted benzylamines 1m–s

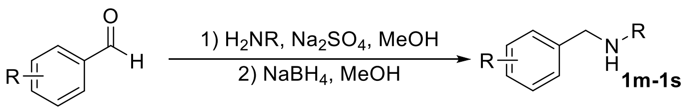

Benzaldehyde (1 equiv), methanol (0.1 M), and primary amine (1.2 equiv) were combined with sodium sulfate (5 equiv) in a round-bottomed flask. The mixture was stirred at room temperature and monitored by thin-layer chromatography (TLC) until complete conversion to the imine was achieved. The sodium sulfate was then filtered off, and a solution of sodium borohydride (2 equiv) in methanol was added dropwise at 0 °C. The reaction mixture was stirred at room temperature and monitored by TLC (PE:EtOAc 4:6) until the reduction of the imine was complete. Excess sodium borohydride was quenched by the addition of water at 0 °C. Methanol was subsequently removed by vacuum concentration. The resulting aqueous phase was basified and extracted with CH_2Cl_2. The organic phases were washed with brine, dried over sodium sulfate, and filtered. Finally, the solvent was removed under reduced pressure to yield the desired product.

*N-Benzyl-N-pentylamine (**1n**)*

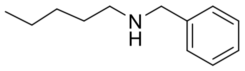
1n

Yellow oil, 55% yield (785 mg)

^{1}H NMR (600 MHz, CDCl3) δ 7.29-7.17 (m, 5H), 3.73 (t, J = 15.0 Hz, 2H), 2.57 (t, J = 7.3 Hz, 2H), 1.48-1.44 (m, 2H), 1.29-1.20 (m, 4H), 0.86-0.80 (m, 3H)

The characterization data are in agreement with those reported in the literature. [4]

*N-Benzylcyclopentanamine (**1o**)*

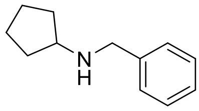
1o

Yellow oil, 78% yield (1.18 g)

^{1}H NMR (600 MHz, $CDCl_3$) δ 7.34-7.26 (m, 5H), 3.78 (d, J = 14.5 Hz, 2H), 3.14-3.11 (m, 1H), 1.64 (m, 8H)

The characterization data are in agreement with those reported in the literature. [5]

*Dibenzylamine (**1p**)*

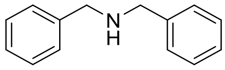
1p

Colorless liquid, 98% yield (1.45 g)

^{1}H NMR (600 MHz, $CDCl_3$) δ 7.30-7.14 (m, 10H partially overlapped with solvent), 3.73 (s, 4H), 1.92 (s, 1H)

The characterization data are in agreement with those reported in the literature. [6]

*Allylbenzylamine (**1q**)*

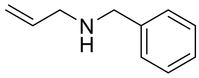

1q

Yellow oil, 87% yield (1,28 g)

^{1}H NMR (600 MHz, $CDCl_3$) δ 7.28-7.17 (m, 5H partially overlapped with solvent), 5.91-5.84 (m, 1H), 5.14 (dd, J = 17.2, 1.7 Hz, 1H), 5.06 (dd, J = 10.3, 1.0 Hz, 1H), 3.73 (s, 2H), 3.22 (d, J = 5.9 Hz, 2H), 1.54 (s, 1H)

The characterization data are in agreement with those reported in the literature. [7]

*Benzyl(2-methylpropyl)amine (**1r**)*

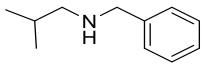

1r

Colorless liquid, 75% yield (1,11 g)

^{1}H NMR (600 MHz, $CDCl_3$) δ 7.28-7.16 (m, 5H partially overlapped with solvent), 3.72 (s, 2H), 2.37 (d, J = 6.9 Hz, 2H), 1.75-1.68 (m, 1H), 0.84 (d, J = 6.5 Hz, 6H)

The characterization data are in agreement with those reported in the literature. [8]

*N-(4-fluorobenzyl)cyclopentanamine (**1s**)*

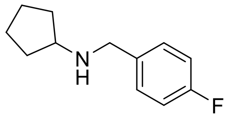

1s

Yellow liquid, 75% yield (400 mg)

^{1}H NMR (600 MHz, $CDCl_3$) δ 7.29-7.26 (m, 2H), 7.01-6.97 (m, 2H), 3.72 (s, 2H), 3.11-3.07 (m, 1H), 1.87-1.82 (m, 2H), 1.73-1.66 (m, 2H), 1.56-1.49 (m, 2H), 1.38-1.32 (m, 2H)

^{13}C NMR (151 MHz, $CDCl_3$) δ 161.8 (d, J = 244.2 Hz, 1C), 136.4 (d, J = 2.89 Hz, 1C), 129.6 (d, J = 7.95 Hz, 1C), 115.05 (d, J = 21.67 Hz, 1C), 59.1, 52.0, 33.1, 24.0

The characterization data are in agreement with those reported in the literature. [9]

Synthesis of substituted benzoxazoles 3–5

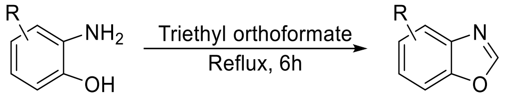

The synthesis was performed in a manner analogous to [10]. Substituted aminophenol (4 mmol) and triethyl orthoformate (5 mL) were combined in a round-bottomed flask, and the mixture was refluxed for 6 hours. The progress of the reaction was monitored by thin-layer chromatography (TLC, PE:EtOAc 7:3). After 6 hours, the reaction mixture was cooled to room temperature, and the triethyl orthoformate was removed under reduced pressure. The resulting crude product was dissolved in 40 mL of distilled water and extracted with $CHCl_3$ (3 × 20 mL). The organic phases were washed with brine, dried over sodium sulfate, and filtered. The solvent was then removed under reduced pressure, yielding the pure product without the need for further purification.

*5-Methylbenzoxazole (**3**)*

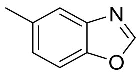

3

Beige crystal solid, 79% yield (425 mg)

^{1}H NMR (600 MHz, $CDCl_3$) δ 8.05 (s, 1H), 7.58-7.57 (m, 1H), 7.45 (d, J = 8.4 Hz, 1H), 7.20 (dtd, J = 8.3, 1.1, 0.5 Hz, 1H), 2.48 (s, 3H)

^{13}C NMR (151 MHz, $CDCl_3$) δ 152.6, 148.2, 140.2, 134.4, 126.7, 120.4, 110.3, 21.4

EI-MS m/z calcd. for C_8H_7NO $[M]^{+\bullet}$: 133.15, found: 133.0

The characterization data are in agreement with those reported in the literature. [10]

*6-Chlorobenzoxazole (**4**)*

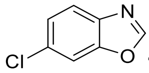

4

Light brown crystal solid, 68% yield (398,6 mg)

^{1}H NMR (600 MHz, $CDCl_3$) δ 8.09 (s, 1H), 7.71 (d, J = 8.5 Hz, 1H), 7.61 (dd, J = 1.9, 0.5 Hz, 1H), 7.36 (dd, J = 8.5, 1.9 Hz, 1H)

^{13}C NMR (151 MHz, $CDCl_3$) δ 153.0, 150.2, 138.8, 131.4, 125.4, 121.1, 111.6

EI-MS m/z calcd. for C_7H_4ClNO $[M]^{+\bullet}$: 153.57, found: 153.0

The characterization data are in agreement with those reported in the literature. [11]

*5-Methoxybenzoxazole (**5**)*

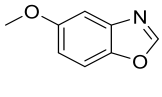

5

Salmon crystal solid, 91% yield (242,3 mg)

^{1}H NMR (600 MHz, $CDCl_3$) δ 8.06 (s, 1H), 7.46 (dd, J = 8.9, 0.3 Hz, 1H), 7.26 (d, J = 3.0 Hz, 1H), 6.99 (ddd, J = 8.9, 2.6, 0.4 Hz, 1H), 3.86 (s, 3H)

^{13}C NMR (151 MHz, $CDCl_3$) δ 157.4, 153.2, 144.6, 140.9, 114.5, 111.1, 103.1, 55.9

EI-MS m/z calcd. for $C_8H_7NO_2$ $[M]^{+\bullet}$: 149.15, found: 149.0

The characterization data are in agreement with those reported in the literature. [10]

Characterization of derivatives 2a open form 2a-o, 2a–s and 3a, 4a, 5a

2-(Piperidin-1-ylmethylideneamino)phenol ***2a****-o*

The product was isolated after conducting the reaction without copper catalyst at 80 °C for 6 hours in the presence of silica, following the optimized procedure for the conventional synthesis of **2a**.

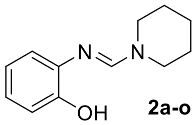

2a-o

^{1}H NMR (600 MHz CDCl$_3$) δ 7.72 (s, 1H), 6.95-6.86 (m, 3H), 6.79-6.77 (m, 1H), 3.67-3.32 (m, 4H), 1.71-1.63 (m, 6H)

The characterization data are in agreement with those reported in the literature. [11]

*2-(Piperidin-1-yl)benzoxazole (****2a****)*

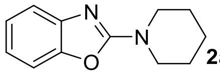

2a

Yellow/brown crystal solid, 99% yield (80 mg)

^{1}H NMR (600 MHz, CDCl$_3$) δ 7.33 (d, J = 7.9 Hz, 1H), 7.22 (d, J = 7.9 Hz, 1H), 7.13 (t, J = 8.1 Hz, 1H), 6.98 (t, J = 7.7 Hz, 1H), 3.64 (m, 4H), 1.67 (m, 6H)

^{13}C NMR (151 MHz, CDCl$_3$) δ 162.5, 148.8, 143.4, 123.9, 120.4, 116.0, 108.6, 46.7, 25.3, 24.1

EI-MS m/z calcd. for $C_{12}H_{14}N_2O$ $[M]^{+\bullet}$: 202.26, found: 202.1

The characterization data are in agreement with those reported in the literature. [12]

*2-(4-Morpholinyl)benzoxazole (****2b****)*

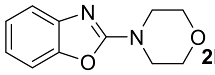

2b

Yellow/brown crystal solid, 83% yield (65 mg)

^{1}H NMR (600 MHz, CDCl$_3$) δ 7.36 (d, J = 7.9 Hz, 1H), 7.25 (d, J = 7.6 Hz, 1H), 7.17 (td, J = 7.7, 0.9 Hz, 1H), 7.03 (td, J = 7.7, 0.9 Hz, 1H), 3.81 (t, J = 4.8 Hz, 4H), 3.68 (t, J = 4.8 Hz, 4H)

^{13}C NMR (151 MHz CDCl$_3$) δ 162.2, 148.8, 142.8, 124.3, 121.1, 116.6, 109.0, 66.3, 45.9

EI-MS m/z calcd. for $C_{11}H_{12}N_2O_2$ $[M]^{+\bullet}$: 204.22, found: 204.1

The characterization data are in agreement with those reported in the literature. [12]

*2-(Pyrrolidin-1-yl)benzoxazole (****2c****)*

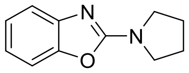

2c

White/yellow crystal solid, 82% yield (67 mg)

^{1}H NMR (600 MHz, CDCl$_3$) δ 7.37 (dq, J = 7.8, 0.5 Hz, 1H), 7.26 (dq, J = 7.9, 0.5 Hz, 1H), 7.17 (td, J = 7.7, 1.1 Hz, 1H), 7.04 (td, J = 7.8, 1.1 Hz, 1H), 3.83-3.81 (m, 4H), 3.70-3.68 (m, 4H)

EI-MS m/z calcd. For $C_{11}H_{12}N_2O$ $[M]^{+\bullet}$: 188.23, found: 188.1

The characterization data are in agreement with those reported in the literature. [12]

*2-(4-Methyl-1-piperazinyl)benzoxazole (**2d**)*

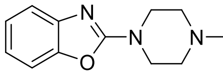
2d

Brown solid, 74% yield (64 mg)

^{1}H NMR (600 MHz, $CDCl_3$) δ 7.35 (d, J = 7.6 Hz, 1H), 7.24 (d, J = 7.9 Hz, 1H), 7.15 (t, J = 7.6 Hz, 1H), 7.01 (t, J = 7.7 Hz, 1H), 3.72 (t, J = 4.8 Hz, 4H), 2.51 (m, 4H), 2.34 (s, 3H)

^{13}C NMR (151 MHz, $CDCl_3$) δ 162.3, 148.8, 143.2, 124.1, 120.8, 116.4, 108.8, 54.3, 46.3, 45.6

EI-MS m/z calcd. For $C_{12}H_{15}N_3O$ $[M]^{+\bullet}$: 217.27, found: 217.1

The characterization data are in agreement with those reported in the literature. [12]

*tert-Butyl 4-(benzoxazol-2-yl)piperazine-1-carboxylate (**2e**)*

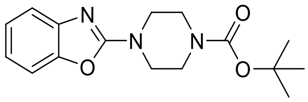
2e

White crystal solid, 73% yield (88 mg)

^{1}H NMR (600 MHz, $CDCl_3$) δ 7.37 (d, J = 7.6 Hz, 1H), 7.27 (d, J = 7.2 Hz, 1H partially overlapped with solvent), 7.18 (td, J = 7.7, 0.9 Hz, 1H), 7.04 (td, J = 7.7, 0.9 Hz, 1H), 3.68 (t, J = 4.6 Hz, 4H), 3.57 (t, J = 4.8 Hz, 4H), 1.49 (s, 9H)

^{13}C NMR (151 MHz, $CDCl_3$) δ 162.0, 154.6, 148.7, 142.9, 124.1, 120.9, 116.5, 108.8, 80.4, 45.4 (two signals merge), 28.4

EI-MS m/z calcd. for $C_{16}H_{21}N_3O_3$ $[M]^{+\bullet}$: 303.36, found: 303.1

The characterization data are in agreement with those reported in the literature. [13]

*2-(3-(Trifluoromethyl)piperidin-1-yl)benzoxazole (**2f**)*

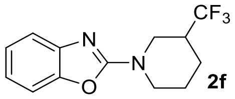
2f

White cream crystal, solid 89% yield (96 mg)

^{1}H NMR (600 MHz, $CDCl_3$) δ 7.37 (d, J = 8.3 Hz, 1H), 7.27 (d, J = 7.6 Hz, 1H partially overlapped with solvent), 7.17 (td, J = 7.7, 1.1 Hz, 1H), 7.04 (td, J = 7.7, 1.3 Hz, 1H), 4.49 (dt, J = 13.1, 2.1 Hz, 1H), 4.31-4.28 (m, 1H), 3.09-3.00 (m, 2H), 2.45-2.35 (m, 1H), 2.13-2.10 (m, 1H), 1.92-1.88 (m, 1H), 1.69-1.55 (m, 2H)

^{13}C NMR (151 MHz, $CDCl_3$) δ 161.8, 148.7, 142.9, 129.0-123.5 (q), 124.0, 120.9, 116.4, 108.8, 45.8, 44.7, 40.2-39.7 (q), 23.3, 23.1

HR-ESI-MS m/z calcd. for $C_{13}H_{14}F_3N_2O$ $[M+H]^{+}$: 271.1053, found: 271.1053

*2-(4-Methoxypiperidin-1-yl)benzoxazole (**2i**)*

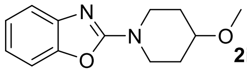
2i

Brown crystal solid, 95% yield (89 mg)

^{1}H NMR (600 MHz, $CDCl_3$) δ 7.34 (d, J = 7.9 Hz, 1H), 7.24 (d, J = 7.9 Hz, 1H), 7.15 (t, J = 7.6 Hz, 1H), 7.00 (t, J = 7.7 Hz, 1H), 3.97-3.93 (m, 2H), 3.52-3.46 (m, 3H), 3.39 (s, 3H), 1.96 (dt, J = 12.6, 3.8 Hz, 2H), 1.74-1.67 (m, 2H)

^{13}C NMR (151 MHz, $CDCl_3$) δ 162.3, 148.8, 143.3, 124.0, 120.6, 116.2, 108.7, 75.1, 55.9, 43.0, 30.0

HR-ESI-MS m/z calcd. for $C_{13}H_{17}N_2O_2$ $[M+H]^+$: 233.1285, found: 233.1280

*1-(Benzo[d]oxazol-2-yl)2,2-dimethyl-propionic acid piperidin-4-yl ester (**2k**)*

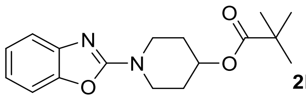

2k

White crystal solid, 68% yield (82 mg)

^{1}H NMR (600 MHz, $CDCl_3$) δ 7.35 (d, J = 8.3 Hz, 1H), 7.24 (d, J = 7.9 Hz, 1H), 7.16 (td, J = 7.7, 1.1 Hz, 1H), 7.02 (td, J = 7.7, 1.0 Hz, 1H), 5.04-5.01 (m, 1H), 3.86-3.82 (m, 2H), 3.69 (qd, J = 6.9, 4.1 Hz, 2H), 1.98 (dq, J = 17.0, 4.1 Hz, 2H), 1.83-1.77 (m, 2H), 1.21 (s, 9H)

^{13}C NMR (151 MHz, $CDCl_3$) δ 177.7, 162.1, 148.7, 143.1, 124.0, 120.6, 116.2, 108.7, 68.1, 42.7, 38.8, 29.8, 27.1

HR-ESI-MS m/z calcd. for $C_{17}H_{23}N_2O_3$ $[M+H]^+$: 303.1703, found: 303.1700

*2-(4-Phenylpiperidin-1-yl)benzoxazole (**2l**)*

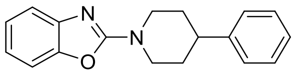

2l

Cream crystal solid, 84% yield (93 mg)

^{1}H NMR (600 MHz, $CDCl_3$) δ 7.37 (dd, J = 7.8, 0.6 Hz, 1H), 7.34-7.31 (m, 2H), 7.27-7.22 (m, 4H), 7.17 (td, J = 7.7, 1.0 Hz, 1H), 7.03 (td, J = 7.8, 1.1 Hz, 1H), 4.47 (dt, J = 13.1, 2.2 Hz, 2H), 3.20 (td, J = 13.0, 2.6 Hz, 2H), 2.78 (tt, J = 12.2, 3.5 Hz, 1H), 1.98 (dt, J = 13.5, 1.5 Hz, 2H), 1.87-1.80 (m, 2H)

^{13}C NMR (151 MHz, $CDCl_3$) δ 162.3, 148.7, 145.2, 143.3, 128.6, 126.7, 126.5, 123.9, 120.5, 116.1, 108.6, 46.4, 42.3, 32.6

HR-ESI-MS m/z calcd. for $C_{18}H_{19}N_2O$ $[M+H]^+$: 279.1492, found: 279.1486

*N-Benzyl-N-ethylbenzoxazol-2-amine (**2m**)*

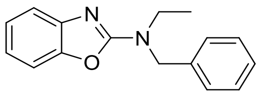

2m

White crystal solid, 55% yield (69 mg)

^{1}H NMR (600 MHz, $CDCl_3$) δ 7.39 (d, J = 7.9 Hz, 1H), 7.36-7.26 (m, 6H), 7.18 (t, J = 7.6 Hz, 1H), 7.02 (t, J = 7.7 Hz, 1H), 4.79 (s, 2H), 3.57 (q, J = 7.1 Hz, 2H), 1.23 (t, J = 7.1 Hz, 3H)

^{13}C NMR (151 MHz, $CDCl_3$) δ 162.7, 148.9, 143.4, 137.0, 128.8, 127.8, 127.8, 124.1, 120.5, 116.1, 108.9, 51.3, 42.8, 12.9

EI-MS m/z calcd. for $C_{16}H_{16}N_2O$ $[M]^{+\bullet}$: 252.31, found: 252.1

The characterization data are in agreement with those reported in the literature. [14]

*N-Benzyl-N-pentylbenzoxazol-2-amine (**2n**)*

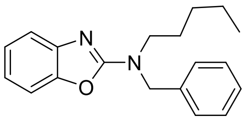

2n

Orange liquid, 63% yield (74 mg)

^{1}H NMR (600 MHz, $CDCl_3$) δ 7.38 (d, J = 7.2 Hz, 1H), 7.35-7.25 (m, 7H), 7.17 (td, J = 7.7, 1.1 Hz, 1H), 7.01 (td, J = 7.7, 1.0 Hz, 1H), 4.79 (s, 2H), 3.48 (t, J = 7.6 Hz, 2H), 1.68-1.63 (m, 2H), 1.34-1.29 (m, 4H), 0.88 (t, J = 7.1 Hz, 3H)

^{13}C NMR (151 MHz, $CDCl_3$) δ 163.1, 149.0, 143.8, 137.1, 128.8, 127.8, 124.0, 120.3, 116.2, 108.8, 51.6, 47.9, 29.0, 27.3, 22.6, 14.1

HR-ESI-MS m/z calcd. for $C_{19}H_{23}N_2O$ $[M+H]^+$: 295.1805, found: 295.1789

*N-Benzyl-N-cyclopentylbenzoxazol-2-amine (**2o**)*

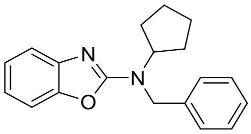

2o

Orange liquid, 61% yield (71 mg)

^{1}H NMR (600 MHz, $CDCl_3$) δ 7.38 (d, J = 7.9 Hz, 1H), 7.23-7.33 (5H), 7.21-7.23 (1H), 7.16 (t, J = 7.7 Hz, 1H), 7.00 (t, J = 7.2 Hz, 1H), 4.75 (s, 2H), 4.68-4.63 (m, 1H), 1.99-1.93 (m, 2H), 1.75-1.57 (m, 7H)

^{13}C NMR (151 MHz, $CDCl_3$) δ 128.7, 127.2, 126.5, 124.0, 116.1, 108.8, 60.0, 48.5, 29.4, 23.6

HR-ESI-MS m/z calcd. for $C_{19}H_{20}N_2O$ $[M+H]^+$: 293.1648, found: 293.1645

*N,N-Dibenzylbenzoxazol-2-amine (**2p**)*

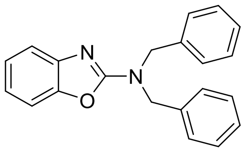

2p

White crystal solid, 53% yield (66 mg)

^{1}H NMR (600 MHz, $CDCl_3$) δ 7.41 (d, J = 7.9 Hz, 1H), 7.35-7.27 (m, 11H), 7.20 (t, J = 7.7 Hz, 1H), 7.04 (t, J = 7.7 Hz, 1H), 4.70 (s, 4H)

^{13}C NMR (151 MHz, $CDCl_3$) δ 163.2, 149.0, 136.4, 128.9, 128.1, 127.9, 124.2, 120.7, 116.4, 109.0, 50.5

EI-MS m/z calcd. for $C_{21}H_{18}N_2O$ $[M]^{+\bullet}$: 314.39, found: 314.1

The characterization data are in agreement with those reported in the literature. [15]

*N-Allyl-N-benzylbenzoxazol-2-amine (**2q**)*

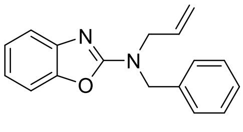

2q

Amber yellow liquid, 57% yield (60 mg)

^{1}H NMR (600 MHz, $CDCl_3$) δ 7.39 (d, J = 7.6 Hz, 1H), 7.35-7.26 (m, 6H), 7.18 (td, J = 7.7, 1.0 Hz, 1H), 7.03 (td, J = 7.7, 1.3 Hz, 1H), 5.90-5.84 (m, 1H), 5.25-5.20 (m, 2H), 4.77 (s, 2H), 4.12 (s, 2H)

^{13}C NMR (151 MHz, $CDCl_3$) δ 162.9, 149.0, 143.6, 136.7, 132.4, 128.8, 128.0, 127.8, 124.1, 120.6, 118.3, 116.4, 108.9, 50.8, 49.8

HR-ESI-MS m/z calcd. for $C_{17}H_{17}N_2O$ $[M+H]^+$: 265.1335, found: 265.1319

N-Benzyl-N-isobutylbenzoxazol-2-amine ***(2r)***

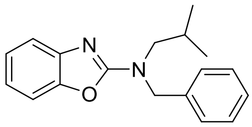

2r

Amber yellow oil, 72% yield (81 mg)

^1H NMR (600 MHz, $CDCl_3$) δ 7.38 (d, J = 7.9 Hz, 1H), 7.25-7.35 (6H), 7.17 (td, J = 7.7, 0.9 Hz, 1H), 7.01 (td, J = 7.7, 1.0 Hz, 1H), 4.82 (s, 2H), 3.32 (d, J = 7.6 Hz, 2H), 2.17-2.10 (m, 1H), 0.94 (d, J = 6.5 Hz, 6H)

^{13}C NMR (151 MHz, $CDCl_3$) δ 163.4, 148.9, 143.7, 136.9, 128.8, 127.7, 124.0, 120.4, 116.2, 108.8, 55.0, 52.0, 26.9, 20.1

HR-ESI-MS m/z calcd. for $C_{18}H_{21}N_2O$ $[M+H]^+$: 281.1648, found: 281.1644

N-Cyclopentyl-N-(4-fluorobenzyl)benzoxazol-2-amine ***(2s)***

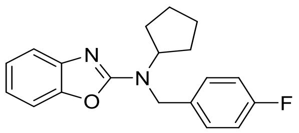

2s

Yellow resin, 87% yield (108 mg)

^1H NMR (600 MHz, $CDCl_3$) δ 7.37 (dq, J = 7.8, 0.6 Hz, 1H), 7.27-7.25 (m, 3H), 7.23 (dq, J = 8.0, 0.5 Hz, 1H), 7.16 (td, J = 7.7, 1.1 Hz, 1H), 7.02-6.98 (m, 3H), 4.71 (s, 2H), 4.65-4.59 (m, 1H), 1.97-1.92 (m, 2H), 1.74-1.69 (m, 2H), 1.61-1.59 (m, 2H), 1.25 (m, 2H)

^{13}C NMR (151 MHz, $CDCl_3$) δ 163.0, 161.9 (d, J = 244.9 Hz), 148.7, 143.2, 134.3 (d, J = 2.9 Hz), 128.0 (d, J = 7.9 Hz), 123.9, 120.3, 116.1, 115.4 (d, J = 21.7 Hz), 108.7, 59.8, 47.8, 29.3, 23.5

HR-ESI-MS m/z calcd. for $C_{19}H_{20}FN_2O$ $[M+H]^+$: 311.1554, found: 311.1531

5-Methyl-2-(piperidin-1-yl)benzoxazole ***(3a)***

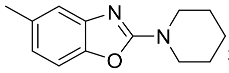

3a

Beige crystal solid, 92% yield (79 mg)

^1H NMR (600 MHz, $CDCl_3$) δ 7.13-7.13 (m, 1H), 7.09 (d, J = 8.1 Hz, 1H), 6.79 (ddd, J = 8.1, 1.7, 0.7 Hz, 1H), 3.64 (d, J = 5.3 Hz, 4H), 2.38 (s, 3H), 1.67 (m, 6H)

^{13}C NMR (151 MHz, $CDCl_3$) δ 162.6, 146.8, 143.4, 133.4, 120.9, 116.4, 107.9, 46.5, 25.2, 24.1, 21.5

EI-MS m/z calcd. for $C_{13}H_{16}N_2O$ $[M]^{+\bullet}$: 216.28, found: 216.1

The characterization data are in agreement with those reported in the literature. [15]

*6-Chloro-2-(piperidin-1-yl)benzoxazole (**4a**)*

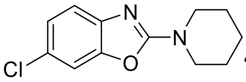

4a

Orange crystal, 60% yield (56 mg)

^{1}H NMR (600 MHz, $CDCl_3$) δ 7.23 (d, J = 1.9 Hz, 1H), 7.21 (d, J = 8.2 Hz, 1H), 7.11 (dd, J = 8.3, 2.0 Hz, 1H), 3.64 (m, 4H), 1.68 (m, 6H)

^{13}C NMR (151 MHz, $CDCl_3$) δ 162.8, 148.9, 142.4, 125.3, 124.2, 116.3, 109.5, 46.7, 25.3, 24.1

EI-MS m/z calcd. for $C_{12}H_{13}ClN_2O$ $[M]^{+\bullet}$: 236.70, found: 236.1

The characterization data are in agreement with those reported in the literature. [11]

*5-Methoxy-2-(piperidin-1-yl)benzoxazole (**5a**)*

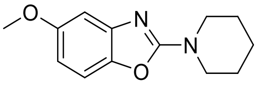

5a

Pink crystal, 87% yield (81 mg)

^{1}H NMR (600 MHz, $CDCl_3$) δ 7.09 (d, J = 8.7 Hz, 1H), 6.91 (d, J = 2.5 Hz, 1H), 6.54 (dd, J = 8.6, 2.5 Hz, 1H), 3.80 (m, 3H), 3.64 (s, 4H), 1.67 (m, 6H)

^{13}C NMR (151 MHz, $CDCl_3$) δ 163.2, 156.9, 144.3, 143.2, 108.3, 106.6, 101.1, 55.8, 46.5, 25.2, 24.0

HR-ESI-MS m/z calcd. for $C_{13}H_{17}N_2O_2$ $[M+H]^{+}$: 233.1285, found: 233.1280

NMR spectra

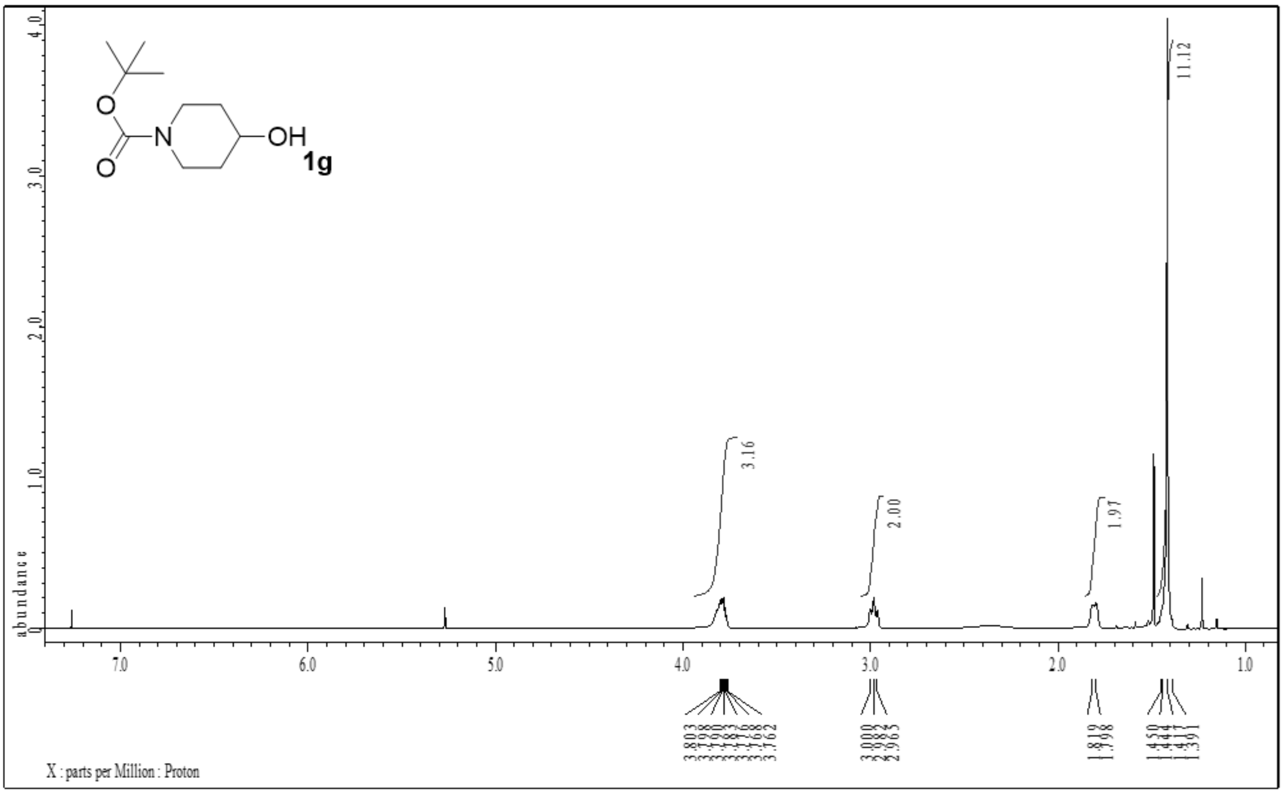

^{1}H NMR (600 MHz, $CDCl_3$) of *tert*-butyl 4-hydroxy piperidine-1-carboxylate (**1g**)

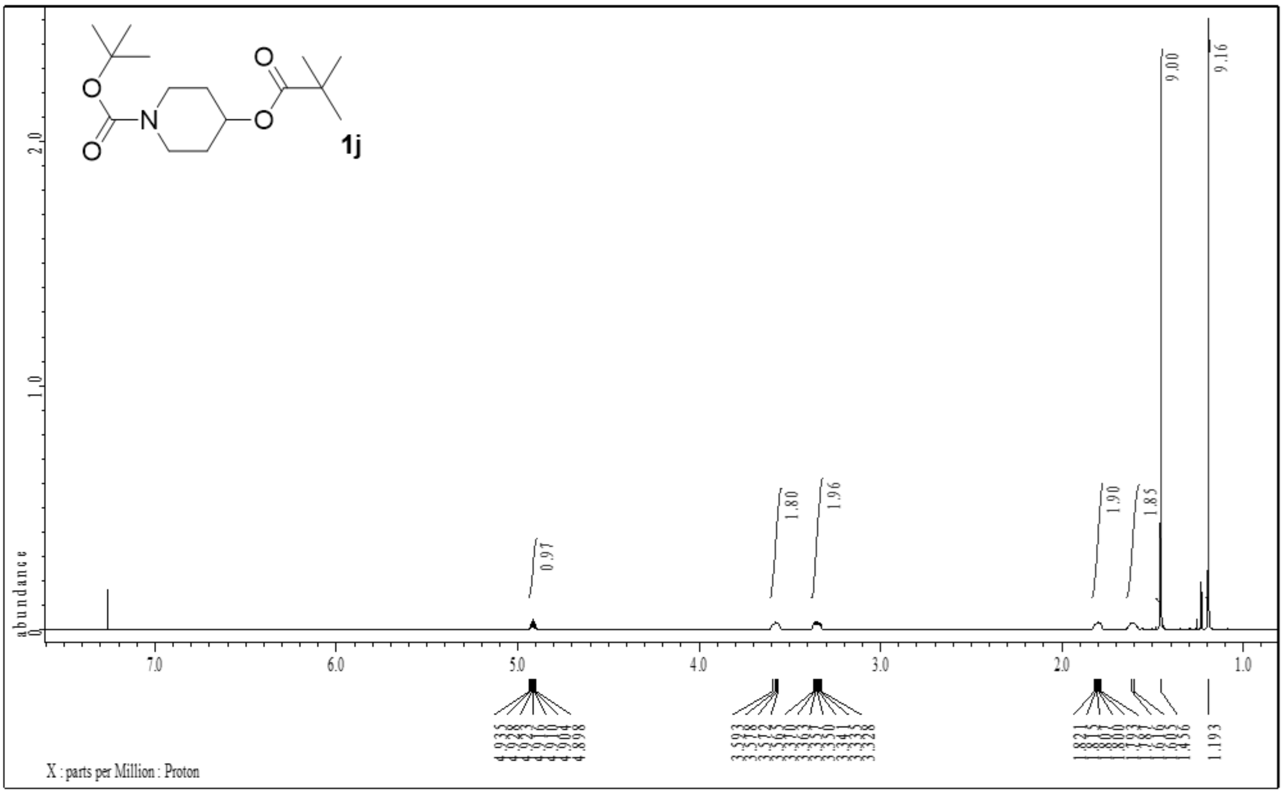

^{1}H NMR (600 MHz, $CDCl_3$) of *tert*-butyl 4-(2,2-dimethylpropanoyloxy)piperidine-1-carboxylate (**1j**)

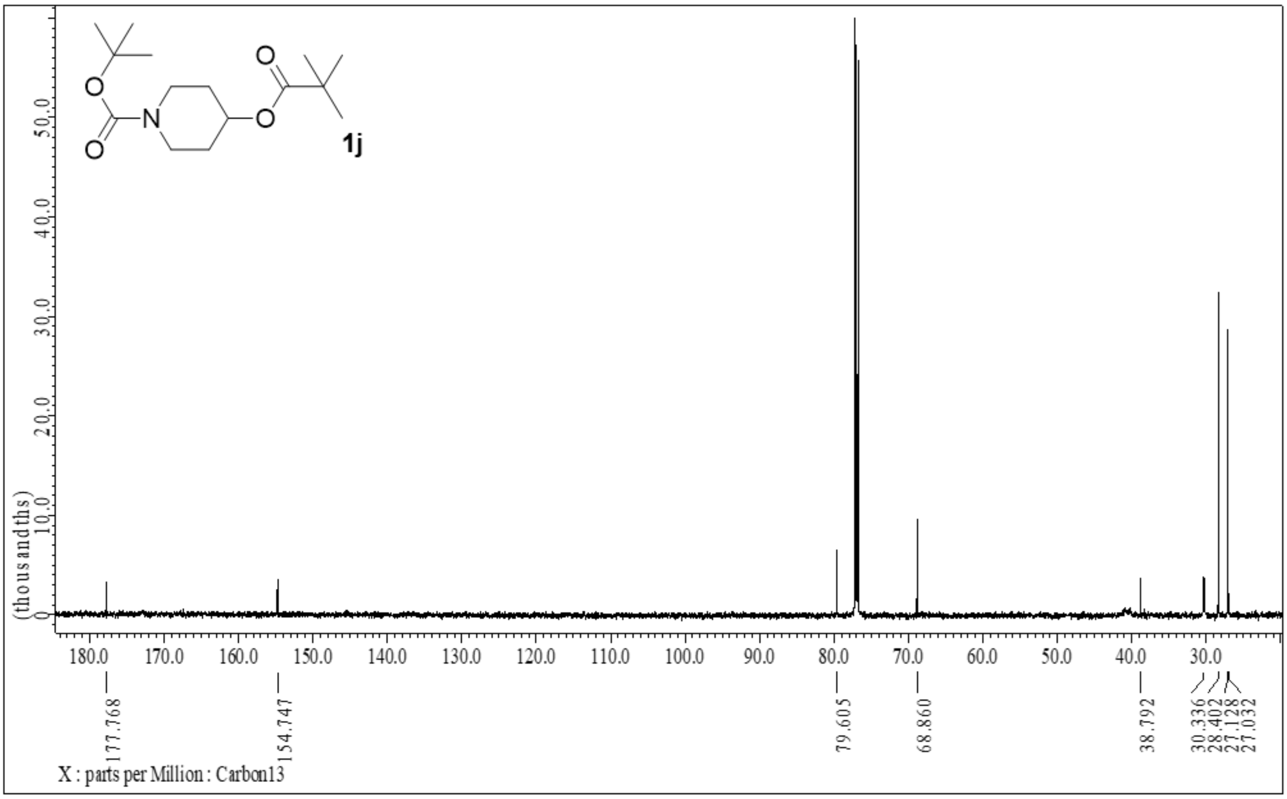

^{13}C NMR (151 MHz, $CDCl_3$) of *tert*-butyl 4-(2,2-dimethylpropanoyloxy)piperidine-1-carboxylate (**1j**)

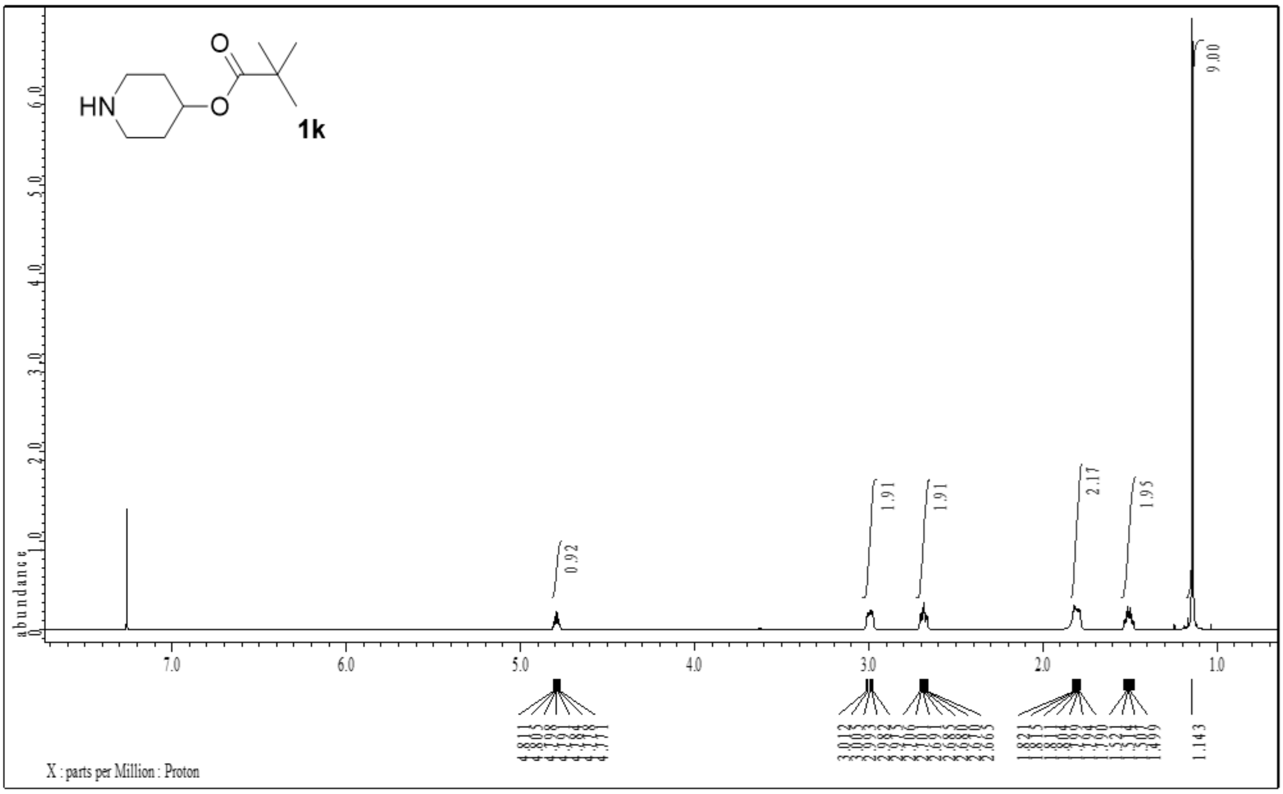

^{1}H NMR (600 MHz, $CDCl_3$) of 2,2-dimethyl-propionic acid piperidin-4-yl ester (**1k**)

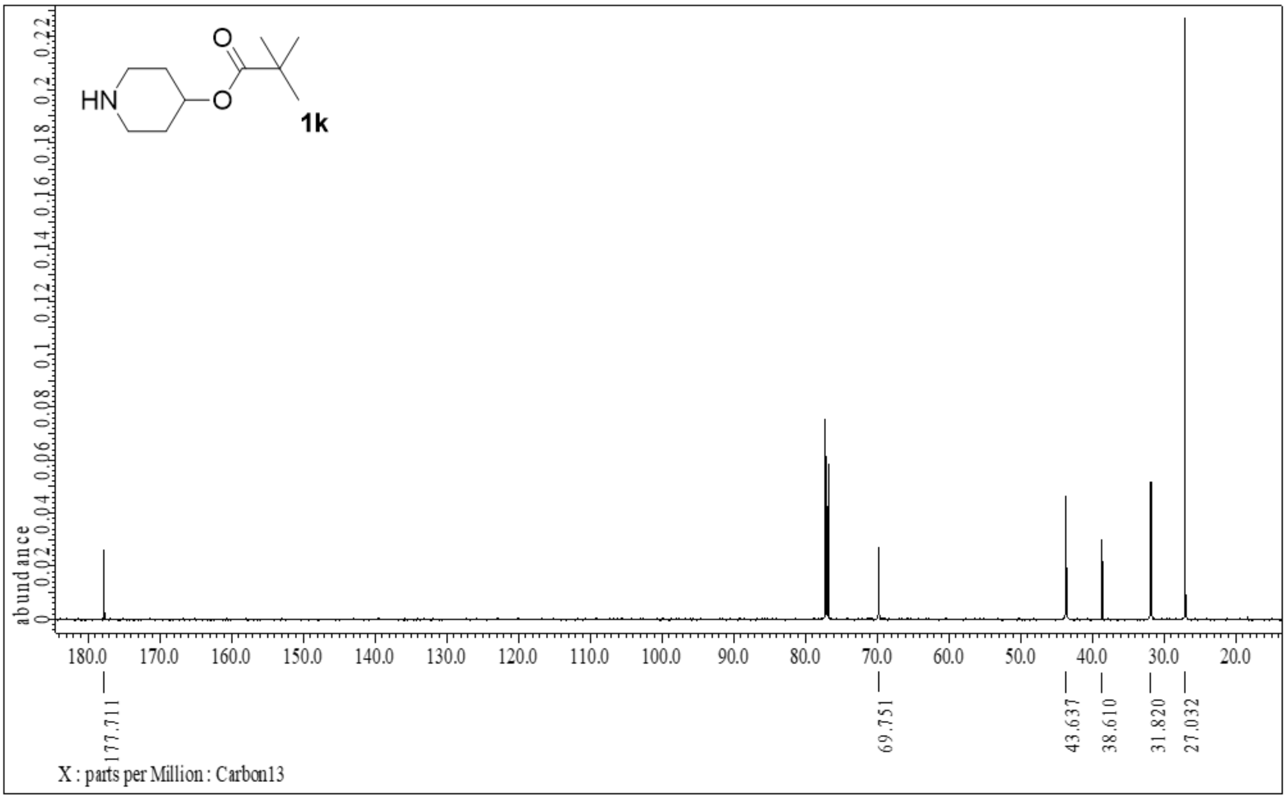

^{13}C NMR (151 MHz, $CDCl_3$) of 2,2-dimethyl-propionic acid piperidin-4-yl ester (**1k**)

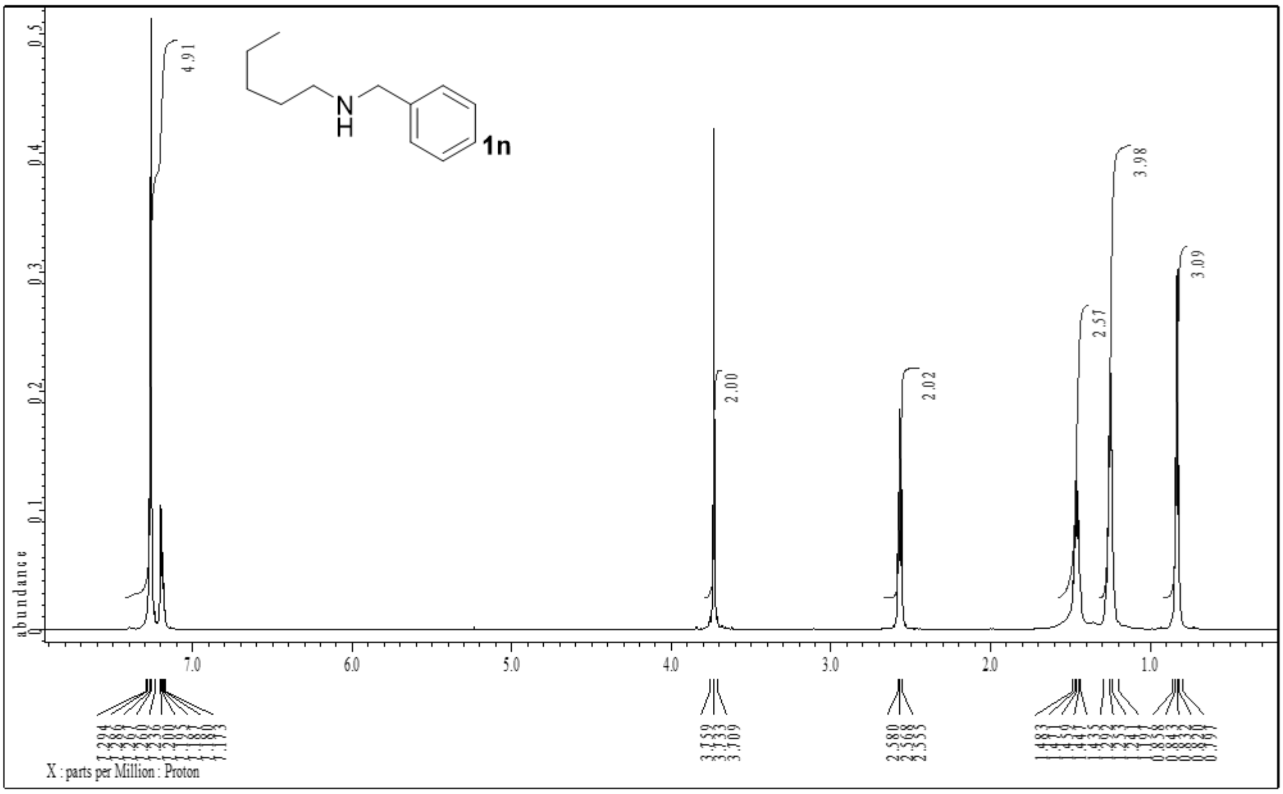

^{1}H NMR (600 MHz, $CDCl_3$) of *N*-benzyl-*N*-pentylamine (**1n**)

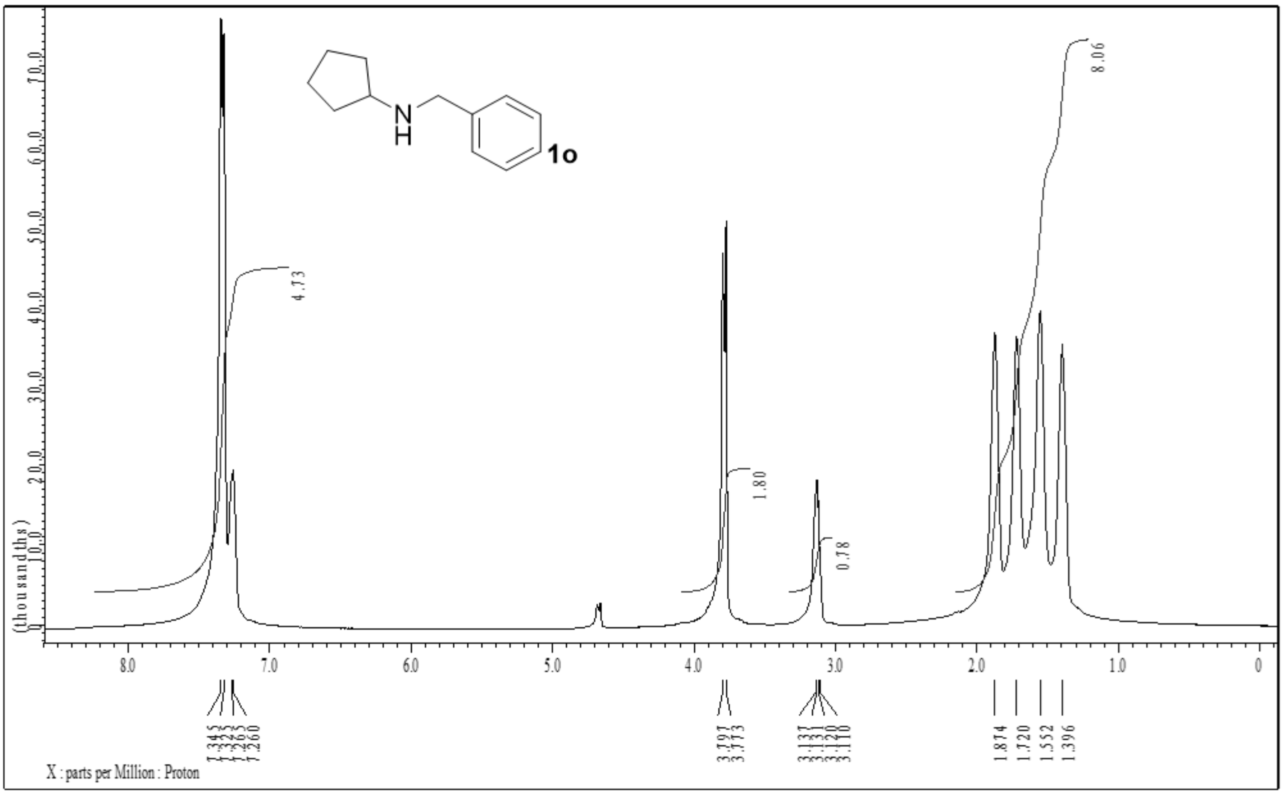

^{1}H NMR (600 MHz, $CDCl_3$) of *N*-benzylcyclopentanamine (**1o**)

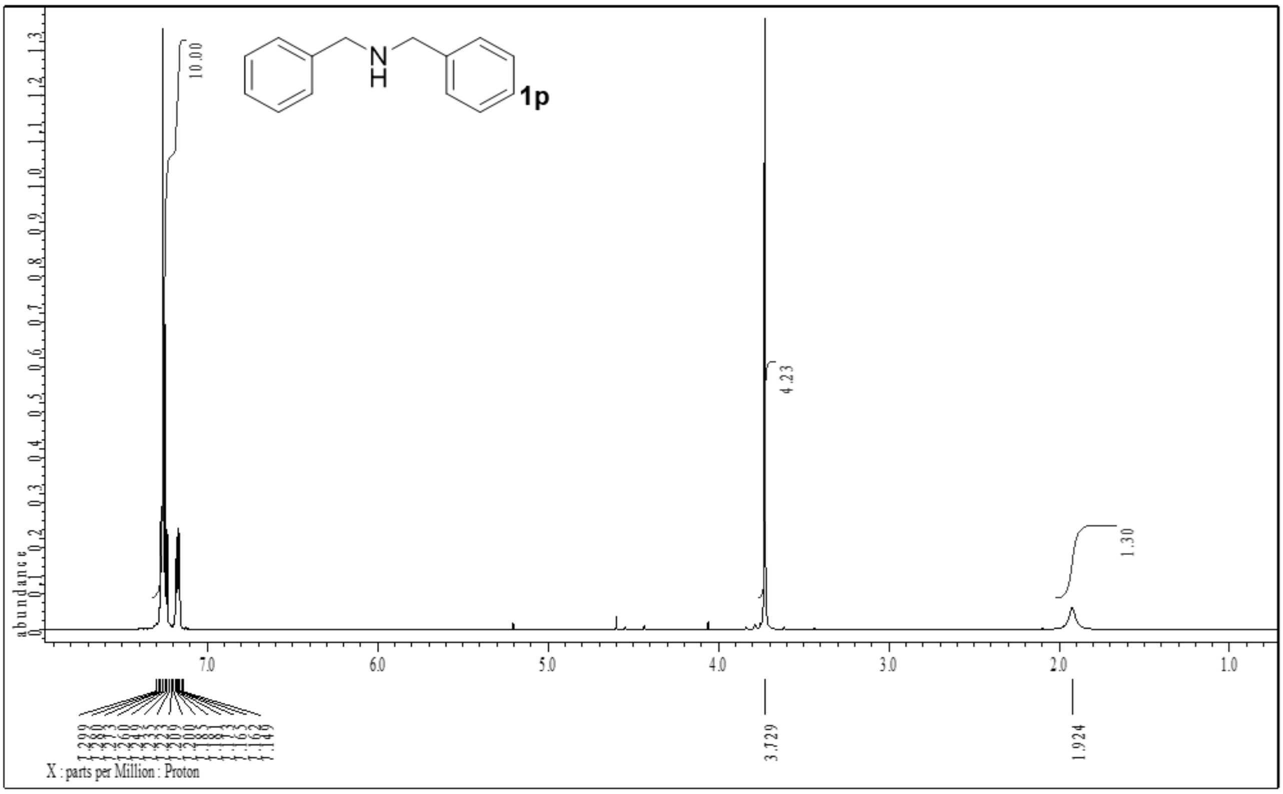

^{1}H NMR (600 MHz, $CDCl_3$) of dibenzylamine (**1p**)

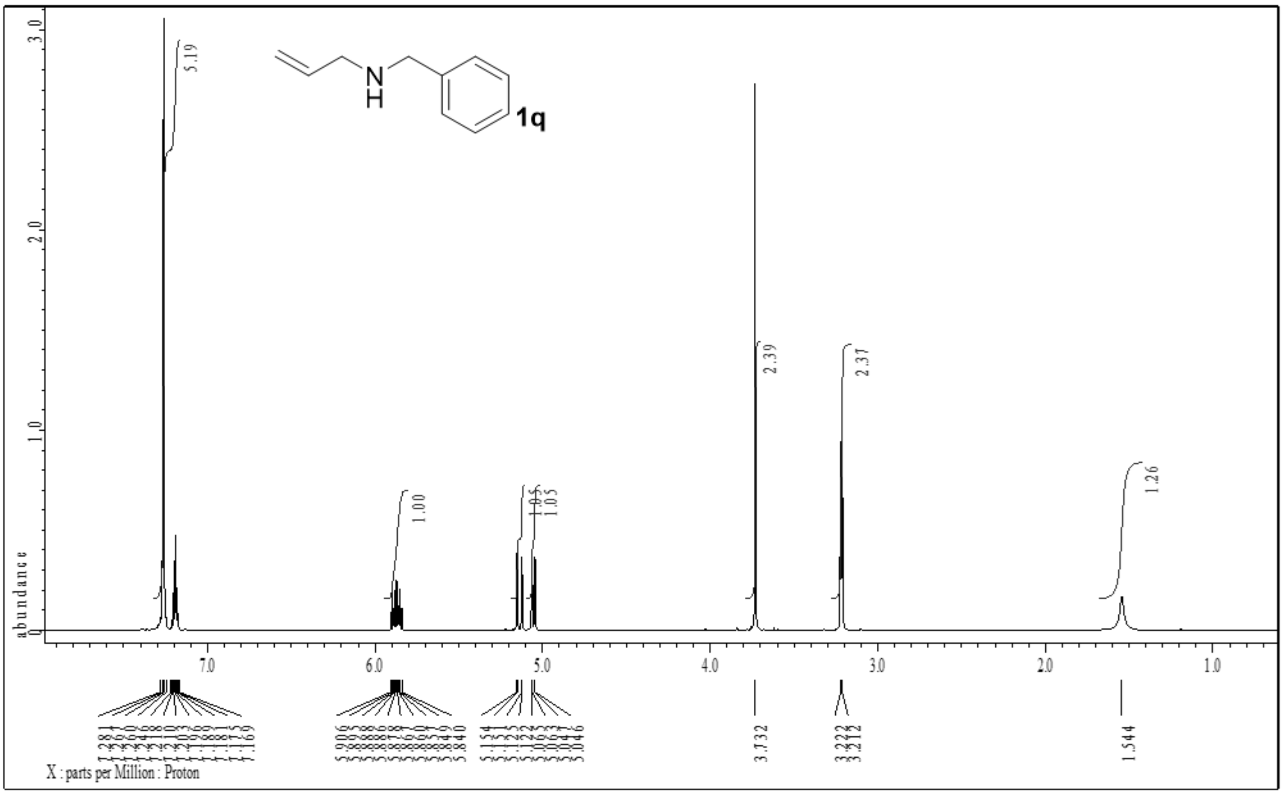

^{1}H NMR (600 MHz, $CDCl_3$) of allylbenzylamine (**1q**)

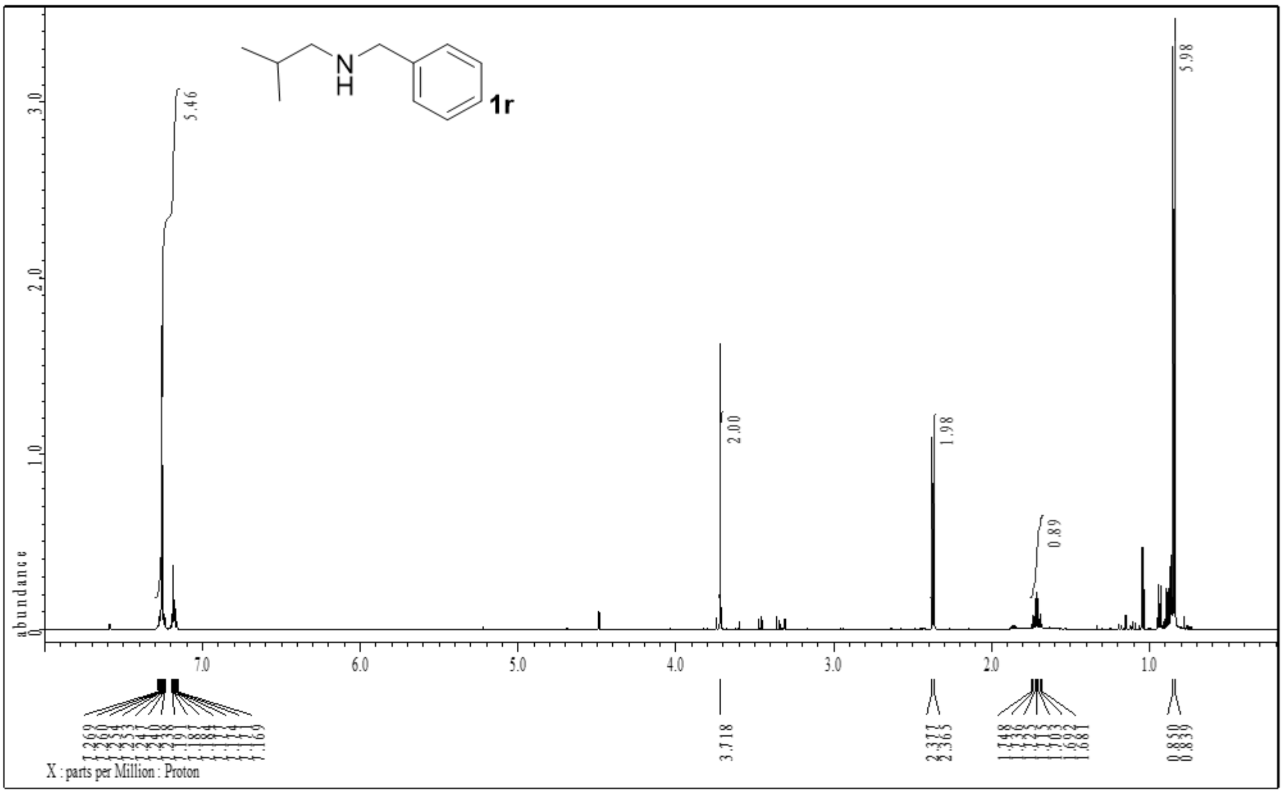

^{1}H NMR (600 MHz, $CDCl_3$) of benzyl(2-methylpropyl)amine (**1r**)

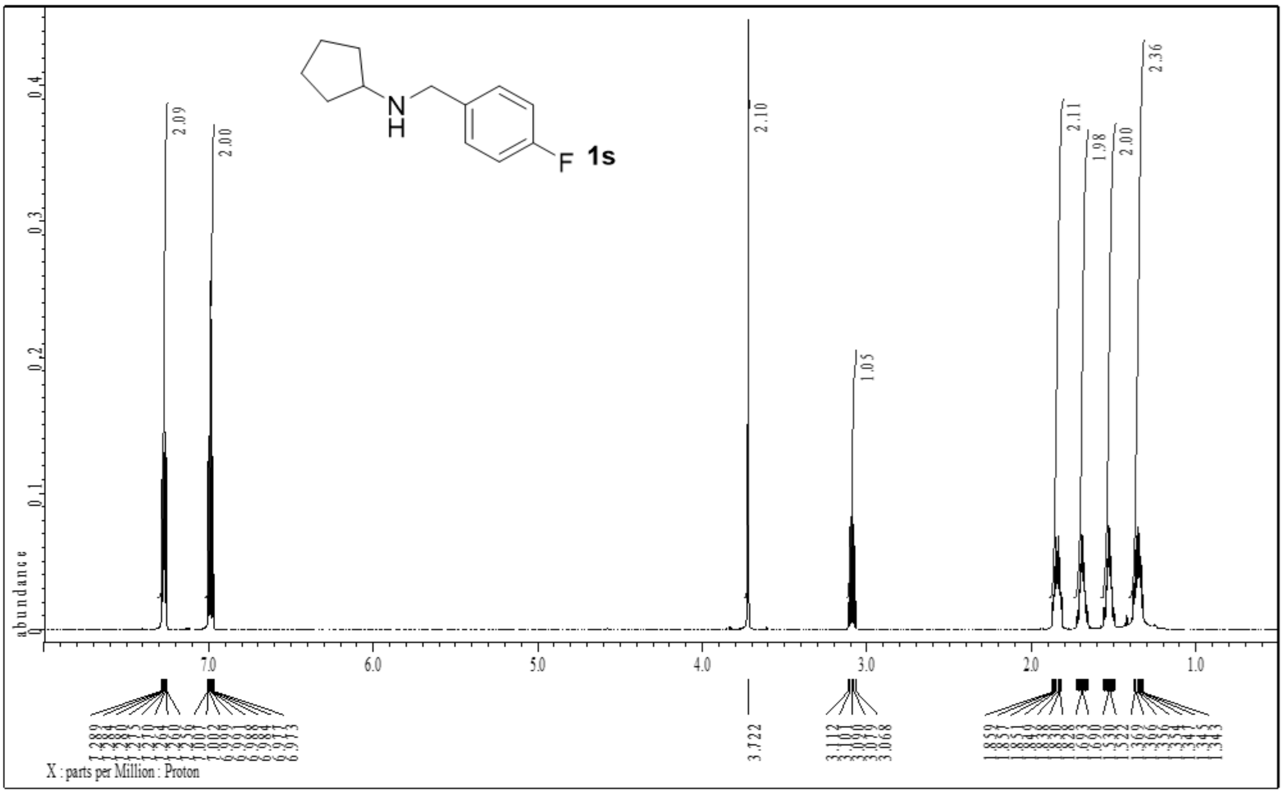

^{1}H NMR (600 MHz, $CDCl_3$) of *N*-(4-fluorobenzyl)cyclopentanamine (**1s**)

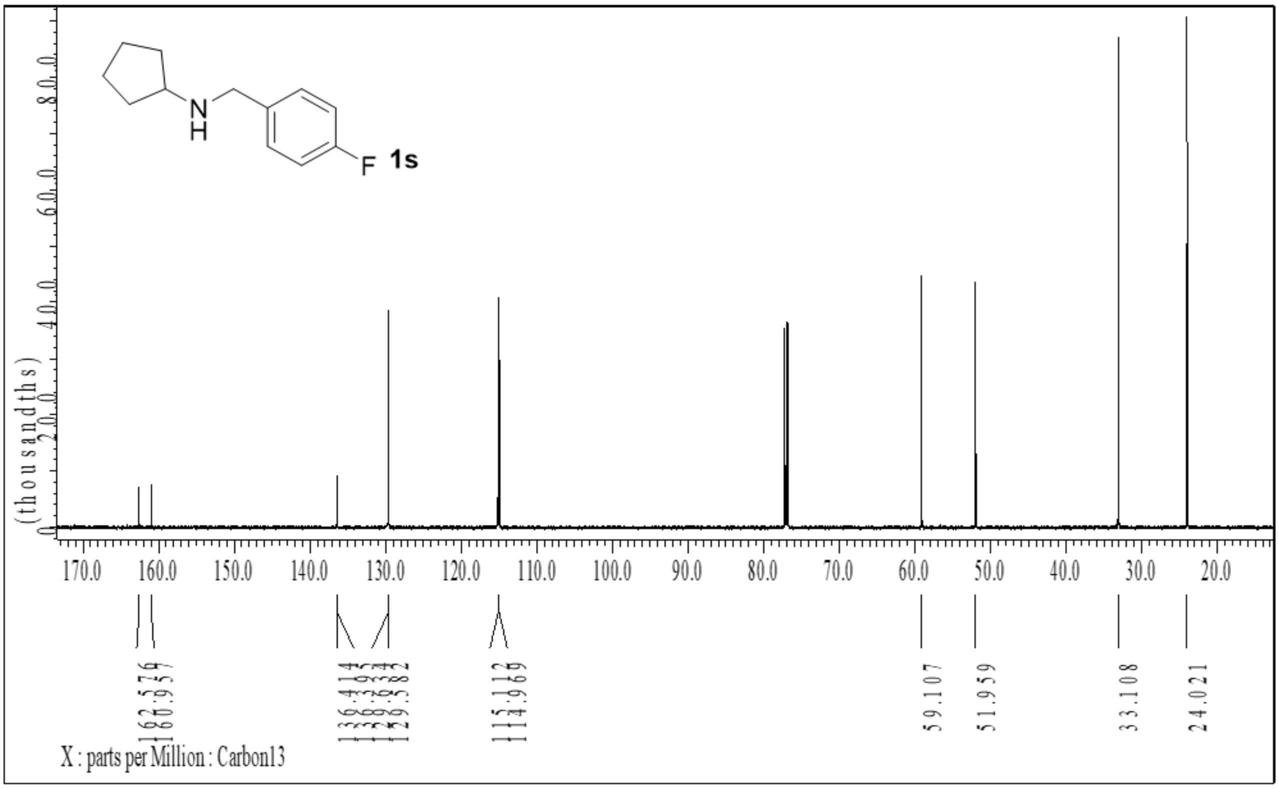

^{13}C NMR (151 MHz, $CDCl_3$) of *N*-(4-fluorobenzyl)cyclopentanamine (**1s**)

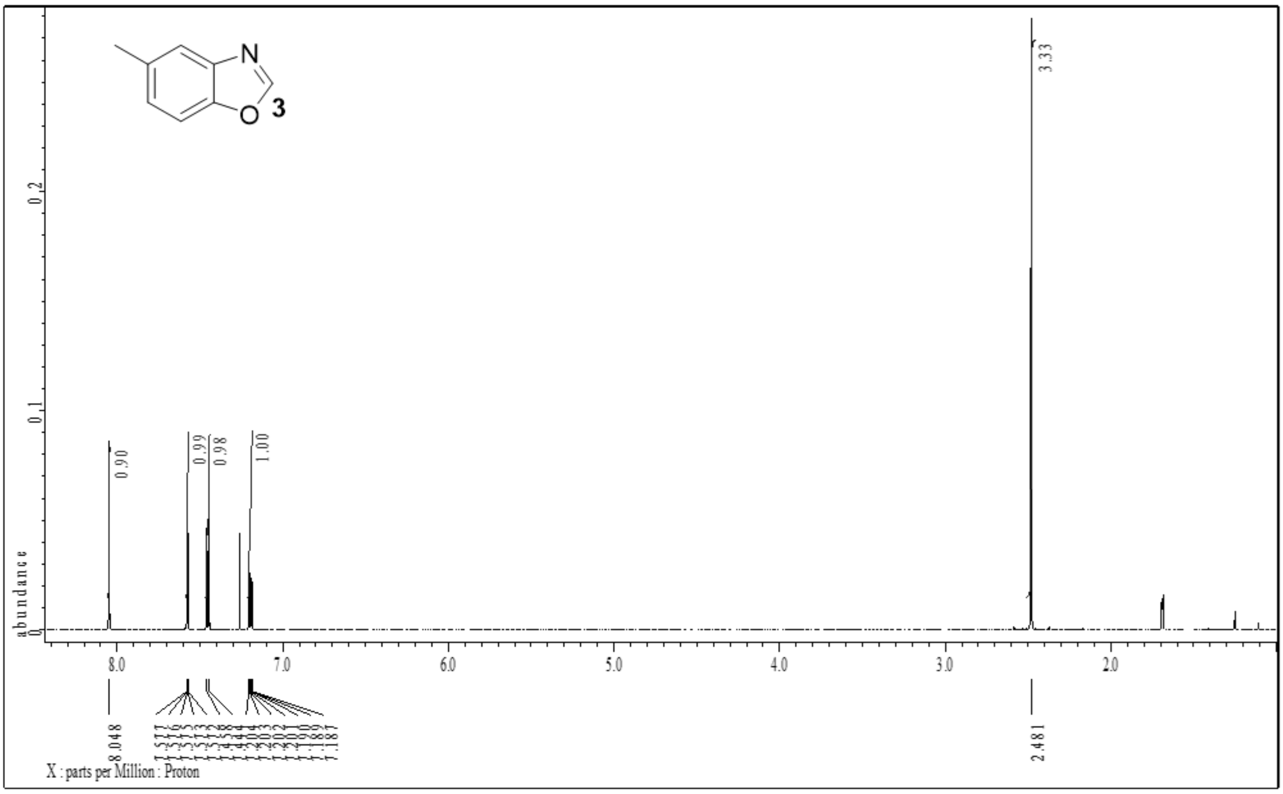

^{1}H NMR (600 MHz, $CDCl_3$) of 5-methylbenzoxazole (**3**)

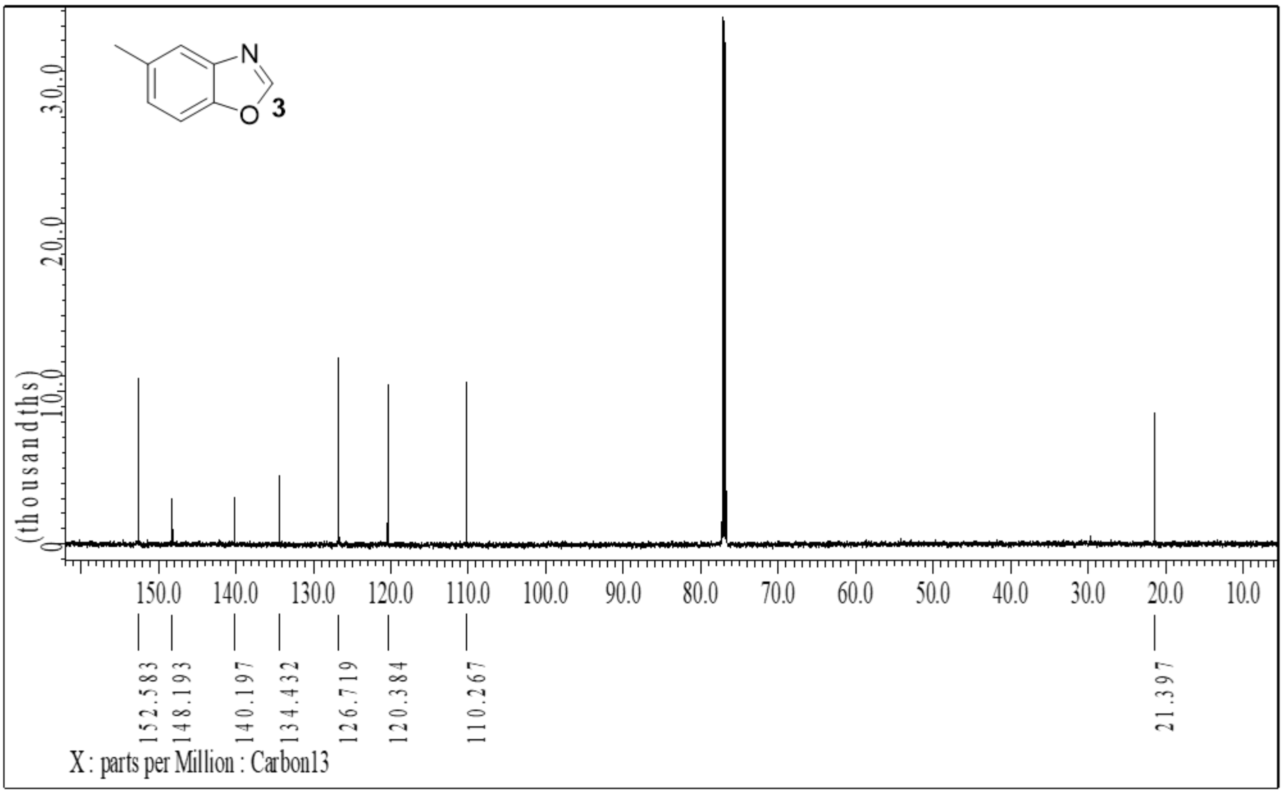

^{13}C NMR (151 MHz, $CDCl_3$) of 5-methylbenzoxazole (**3**)

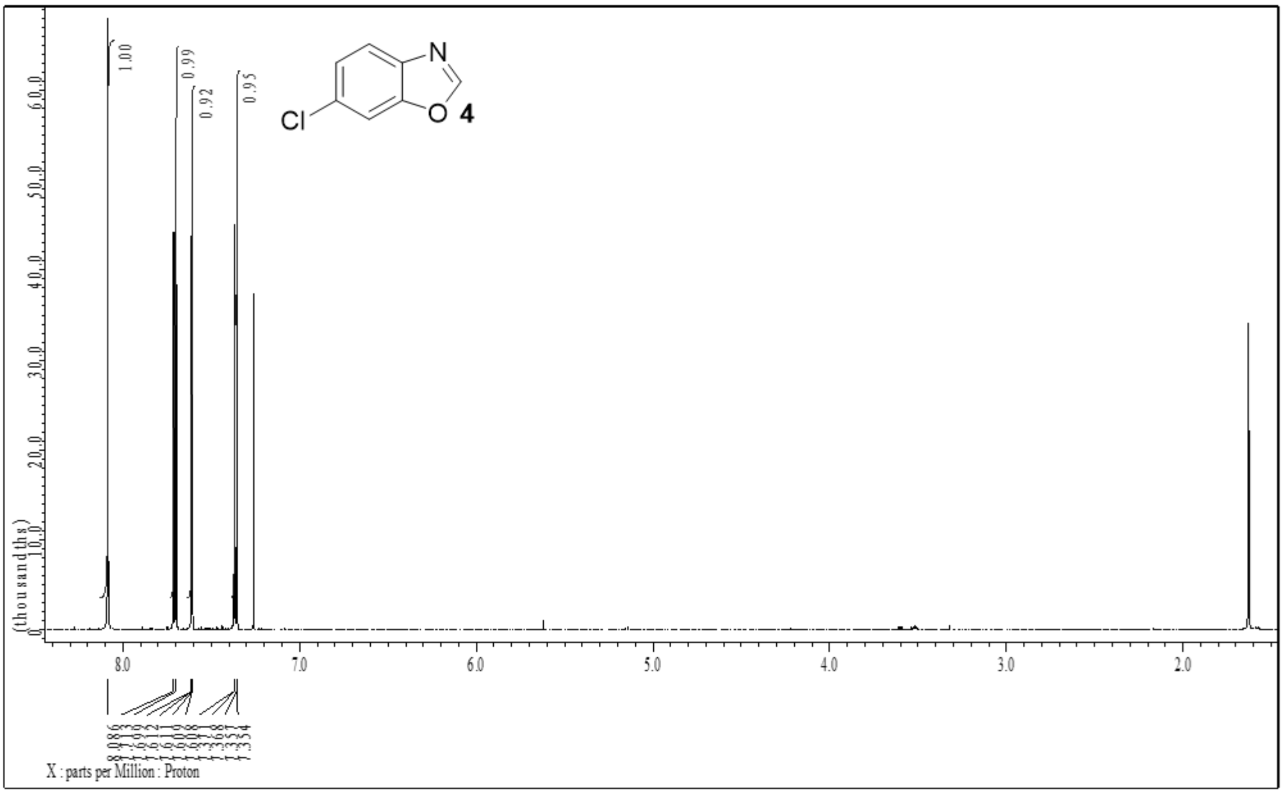

^{1}H NMR (600 MHz, $CDCl_3$) of 6-chlorobenzoxazole (**4**)

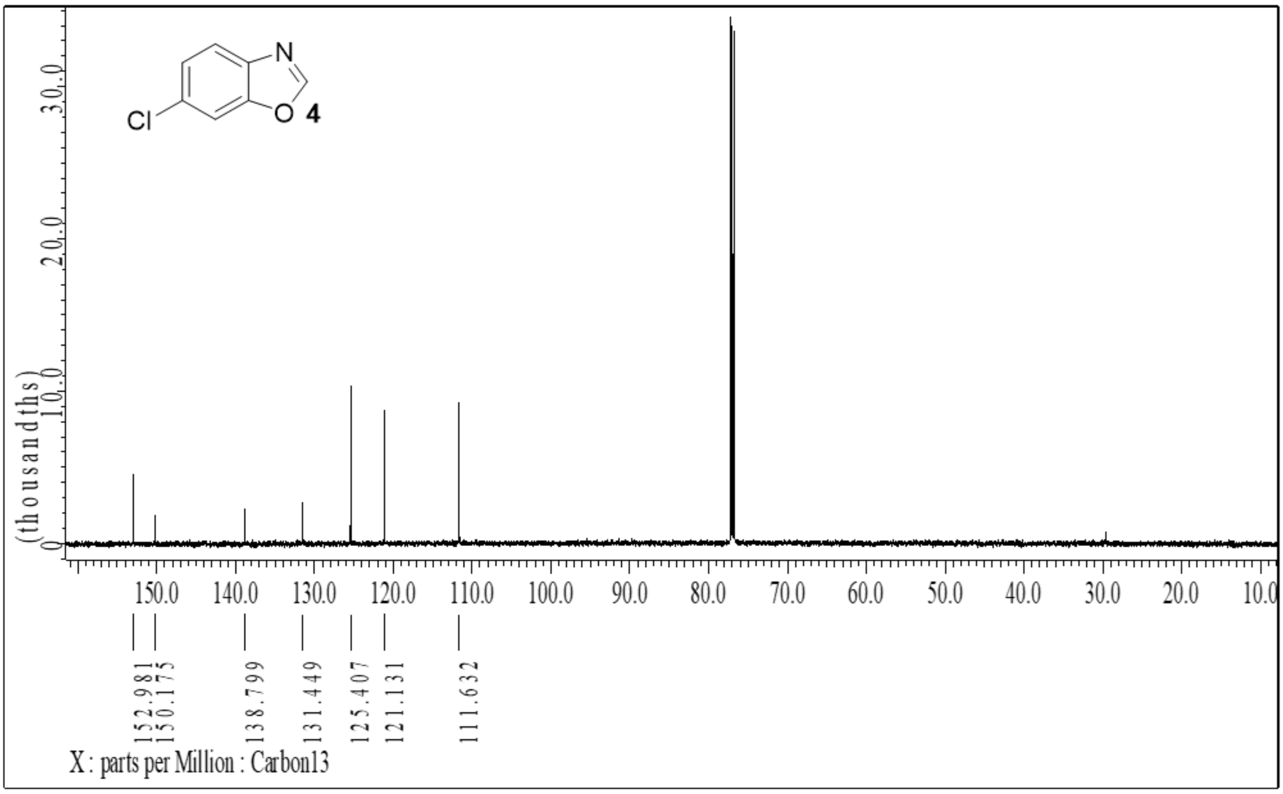

^{13}C NMR (151 MHz, $CDCl_3$) of 6-chlorobenzoxazole (**4**)

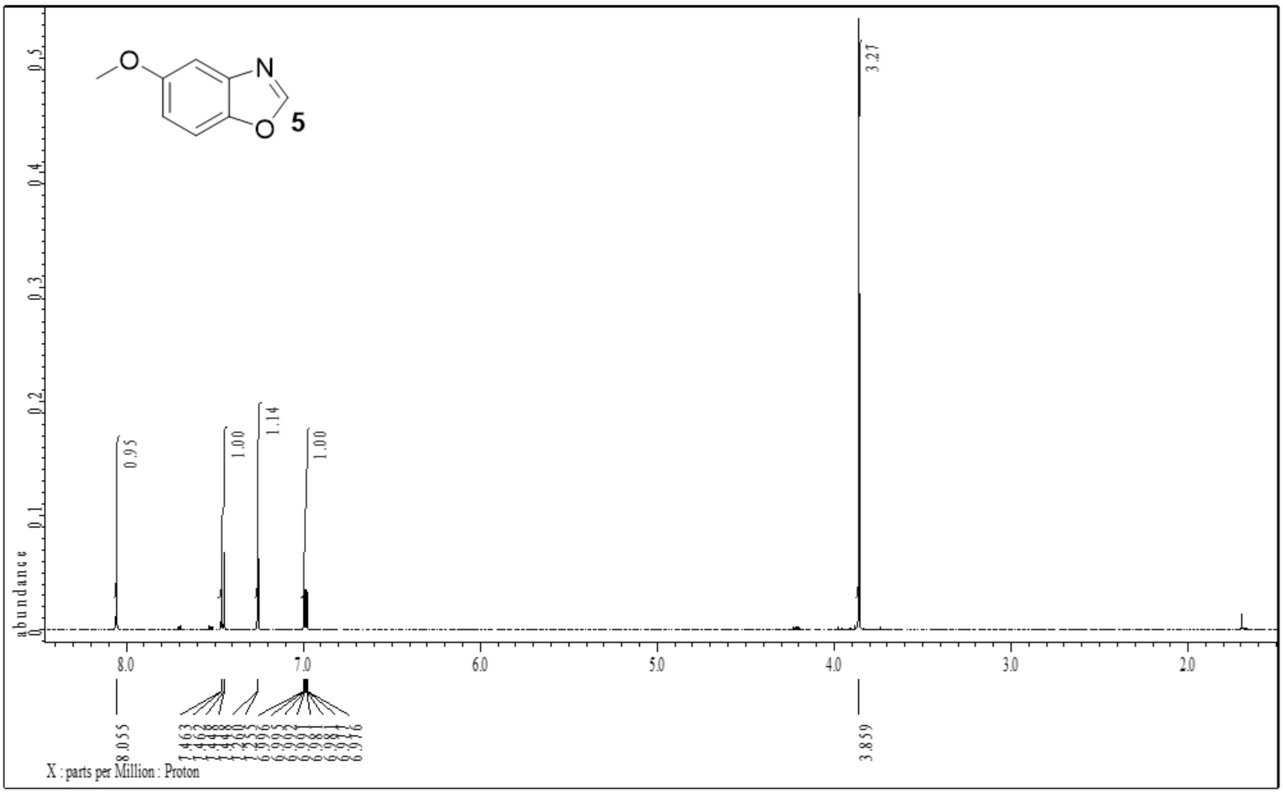

^{1}H NMR (600 MHz, $CDCl_3$) of 5-methoxybenzoxazole (**5**)

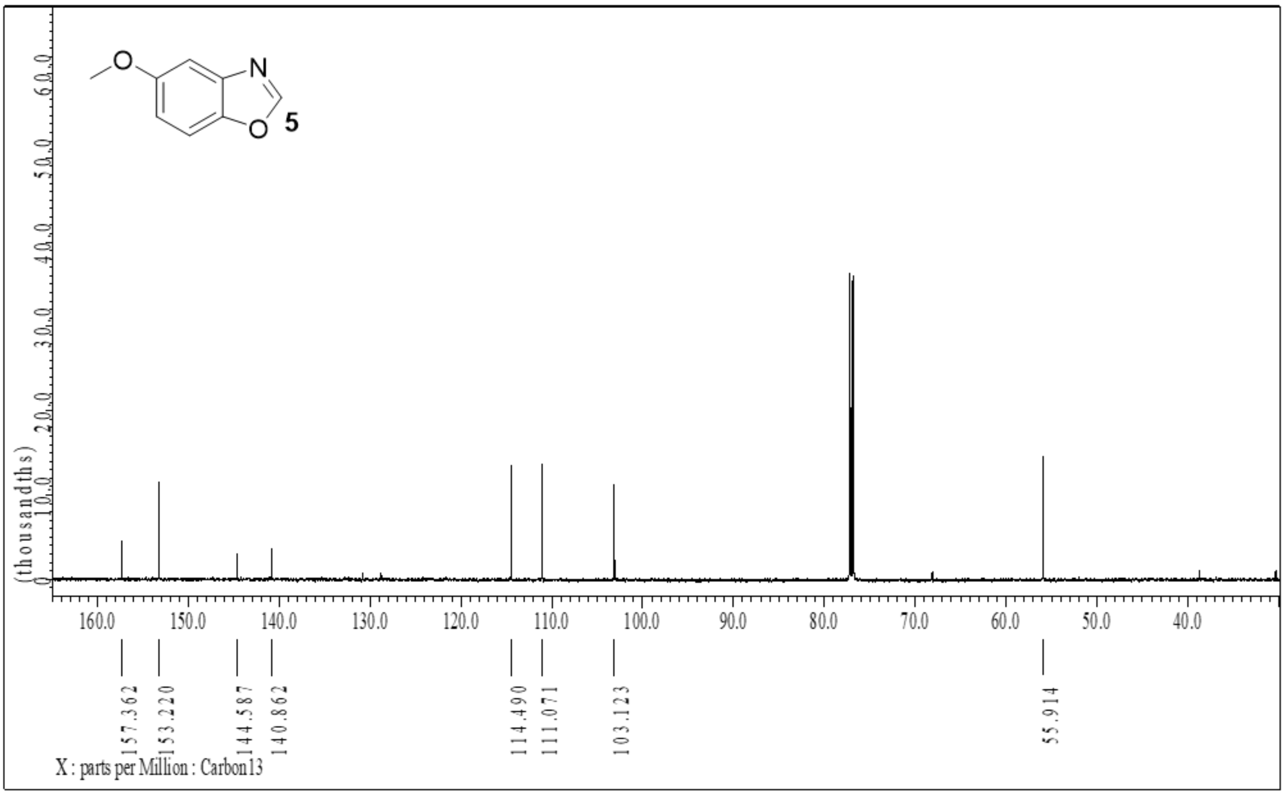

^{13}C NMR (151 MHz, $CDCl_3$) of 5-methoxybenzoxazole (**5**)

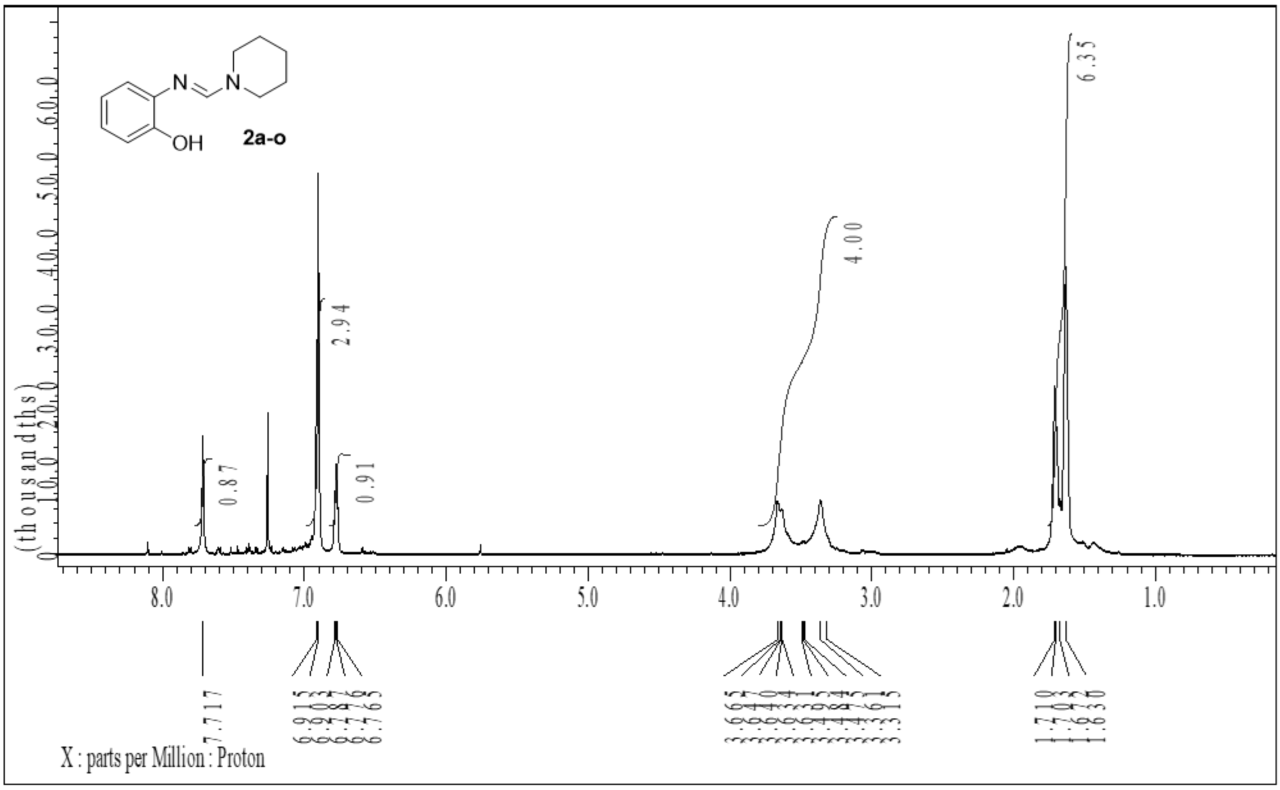

^{1}H NMR (600 MHz, $CDCl_3$) of 2-(piperidin-1-ylmethylideneamino)phenol (**2a**-o)

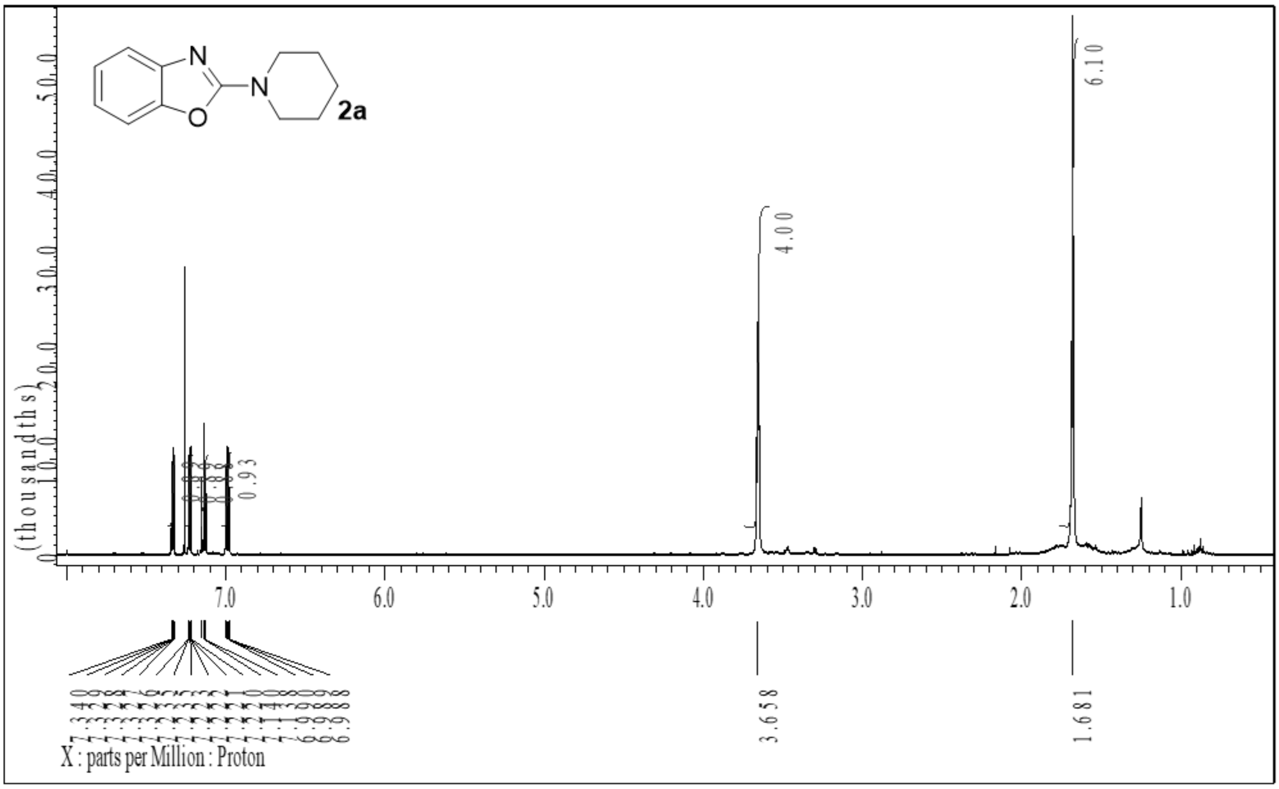

^{1}H NMR (600 MHz, CDCl3) of 2-(piperidin-1-yl)benzoxazole (**2a**)

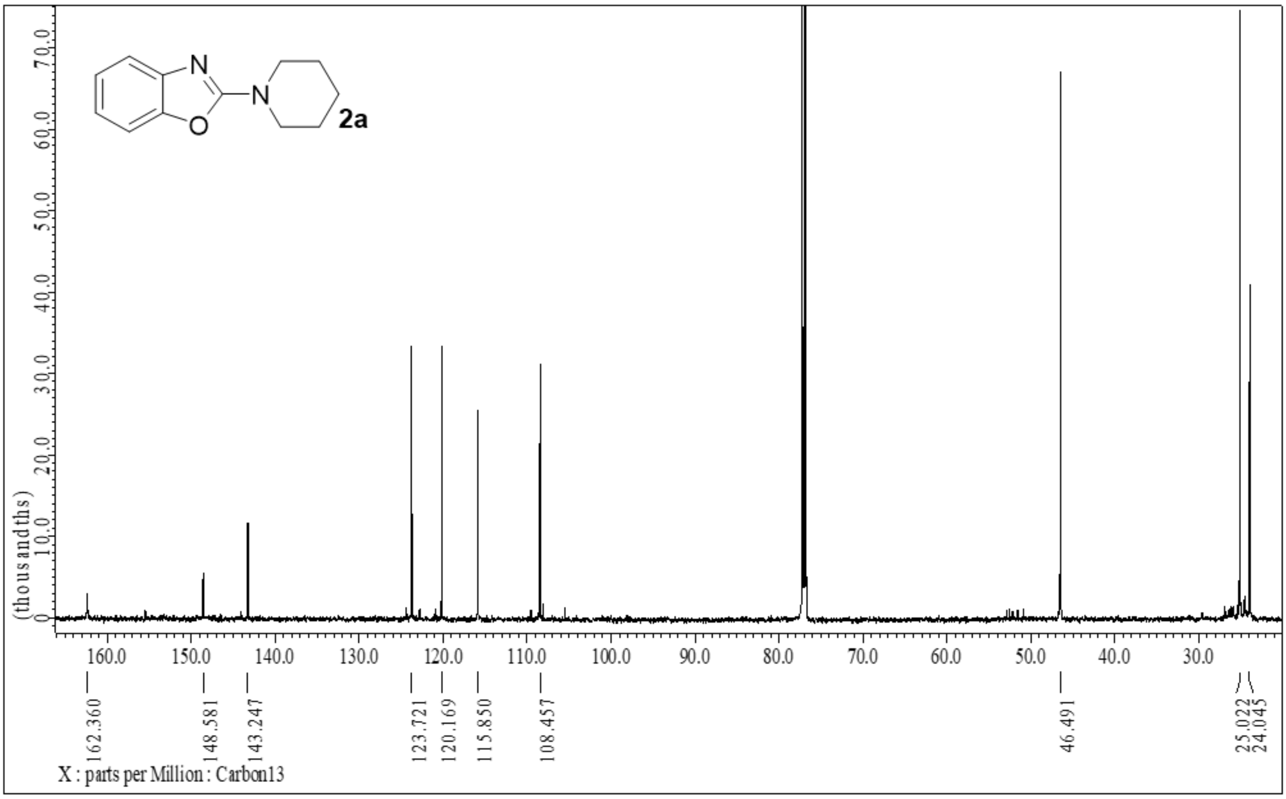

^{13}C NMR (151 MHz, $CDCl_3$) of 2-(piperidin-1-yl)benzoxazole (**2a**)

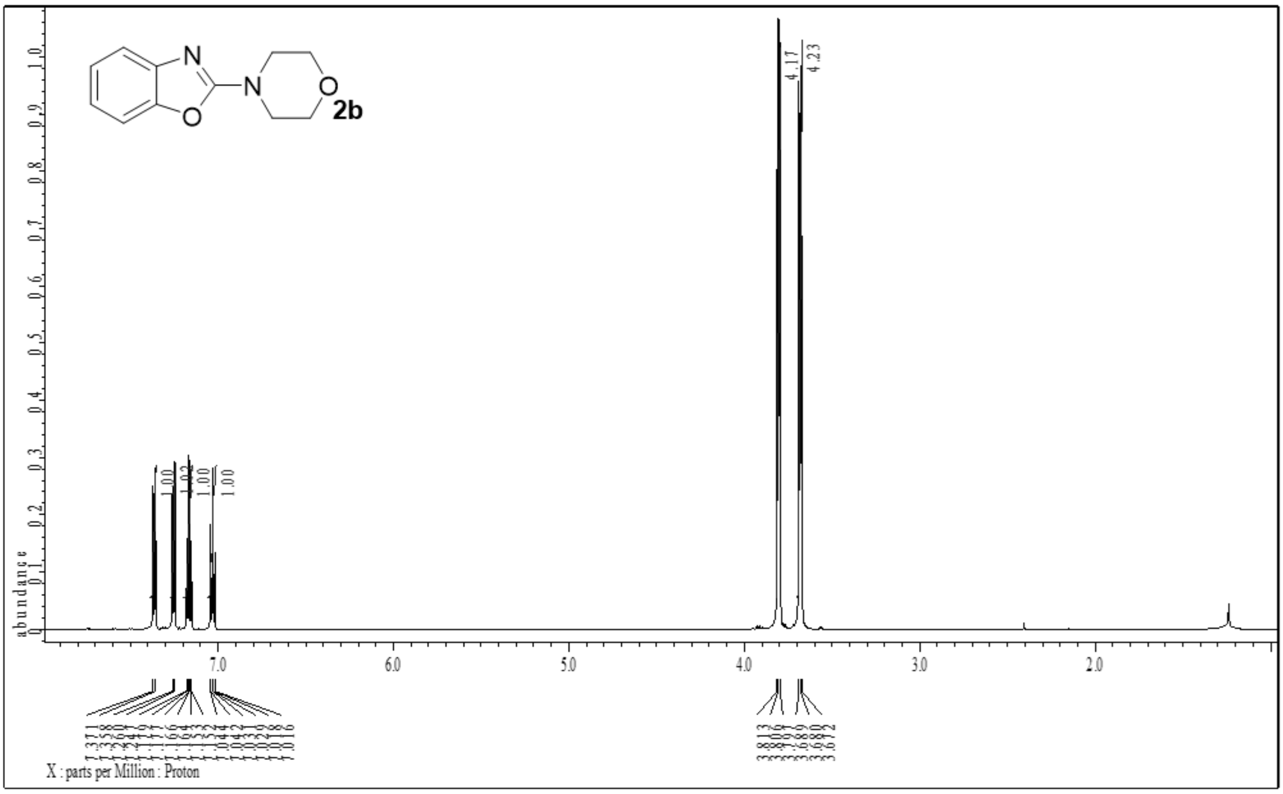

^{1}H NMR (600 MHz, $CDCl_3$) of 2-(4-morpholinyl)benzoxazole (**2b**)

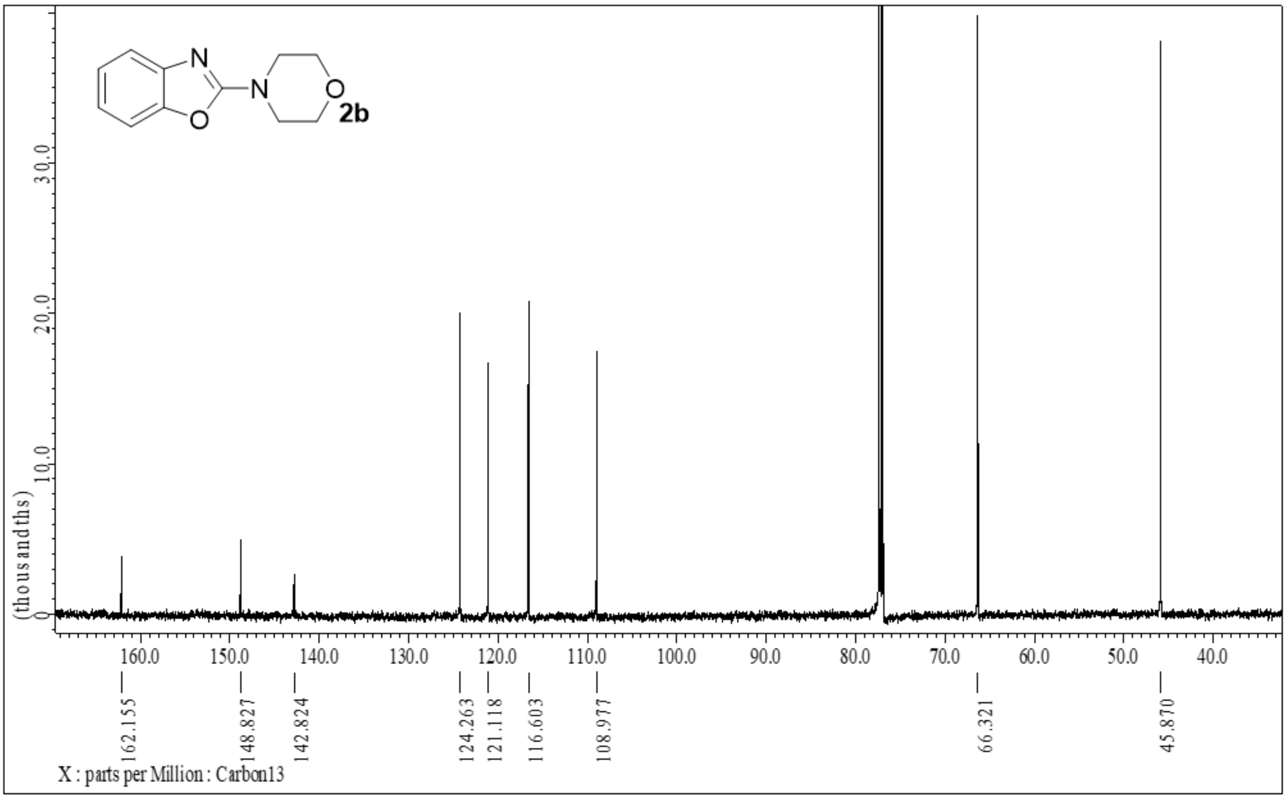

^{13}C NMR (151 MHz, $CDCl_3$) of 2-(4-morpholinyl)benzoxazole (**2b**)

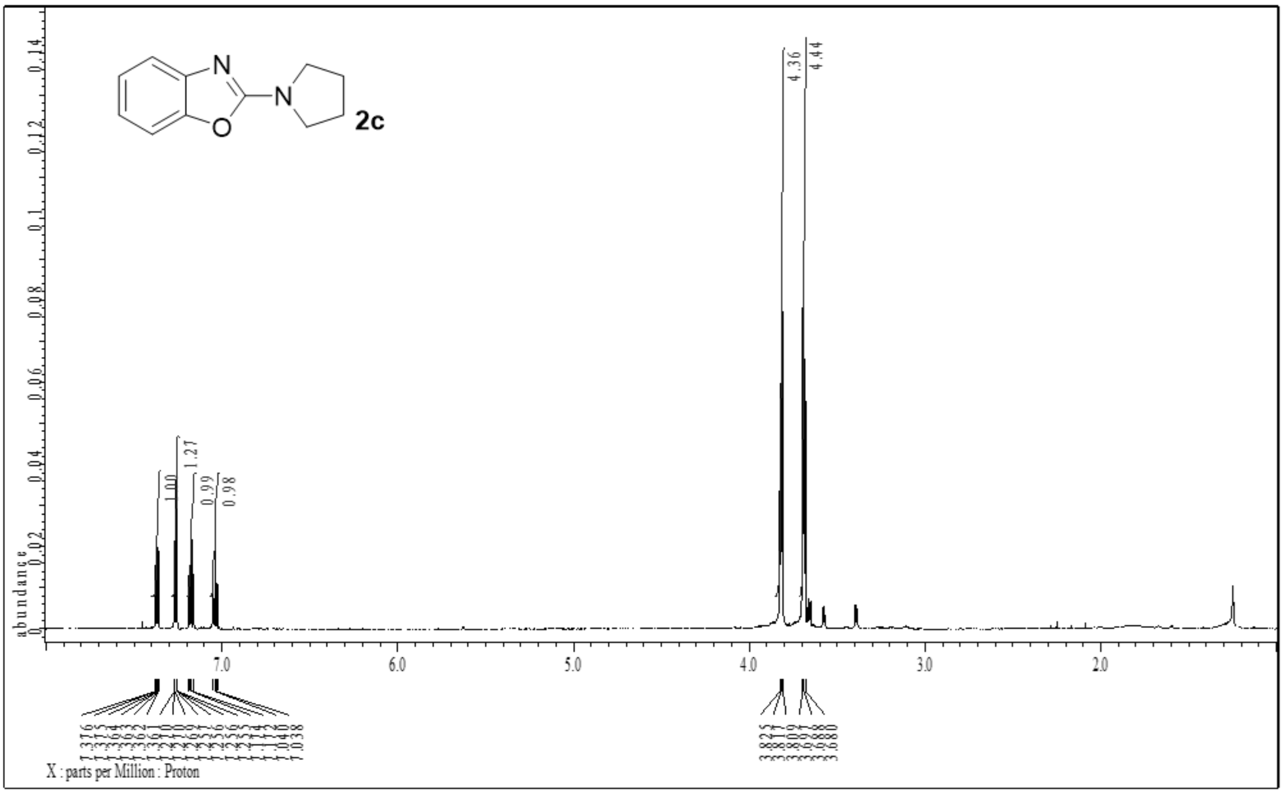

^{1}H NMR (600 MHz, $CDCl_3$) of 2-(pyrrolidin-1-yl)benzoxazole (**2c**)

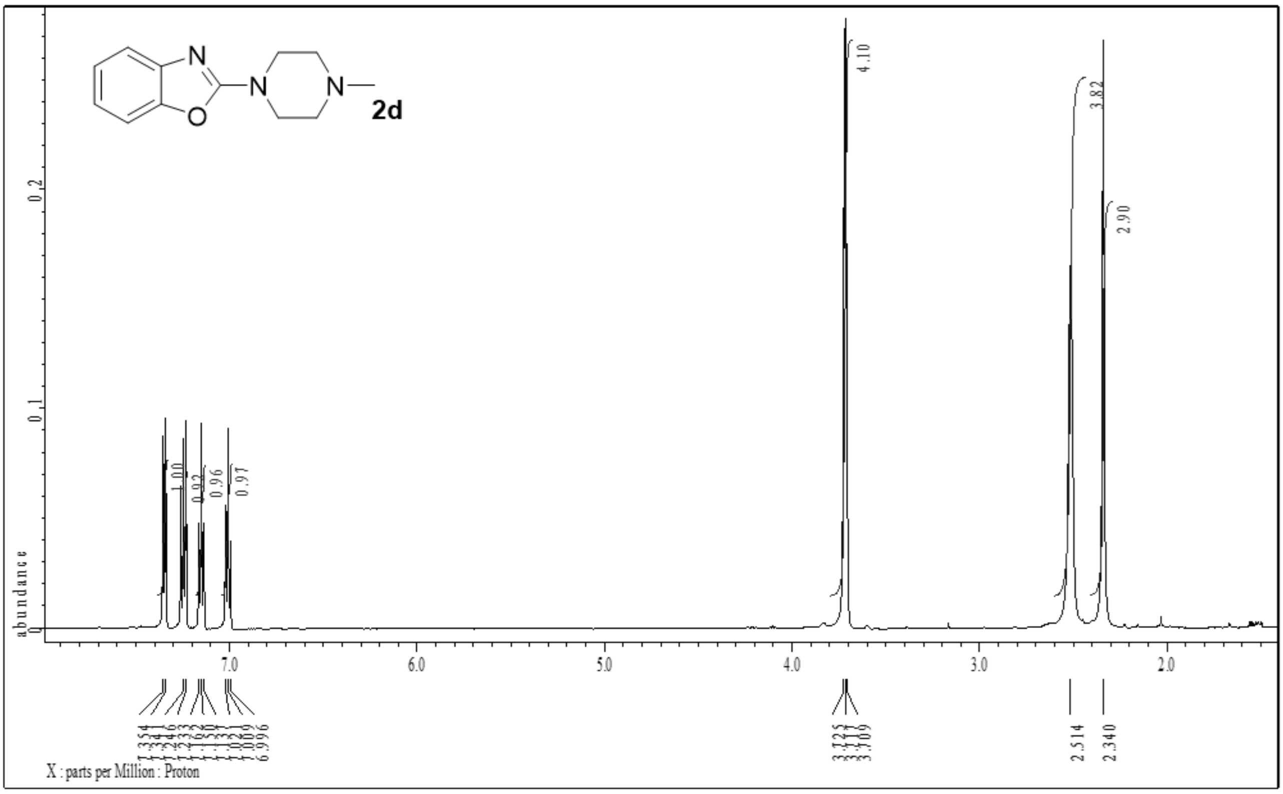

^{1}H NMR (600 MHz, $CDCl_3$) of 2-(4-methyl-1-piperazinyl)benzoxazole (**2d**)

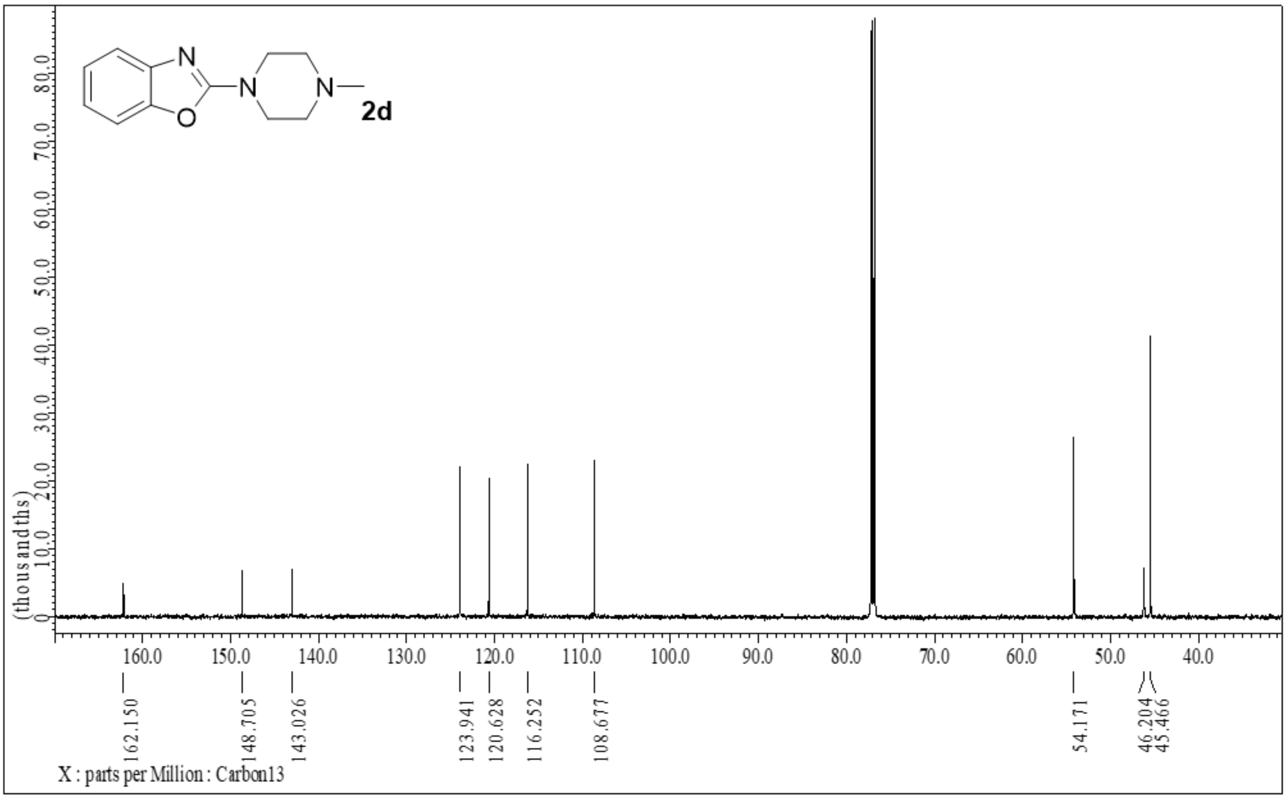

^{13}C NMR (151 MHz, $CDCl_3$) of 2-(4-methyl-1-piperazinyl)benzoxazole (**2d**)

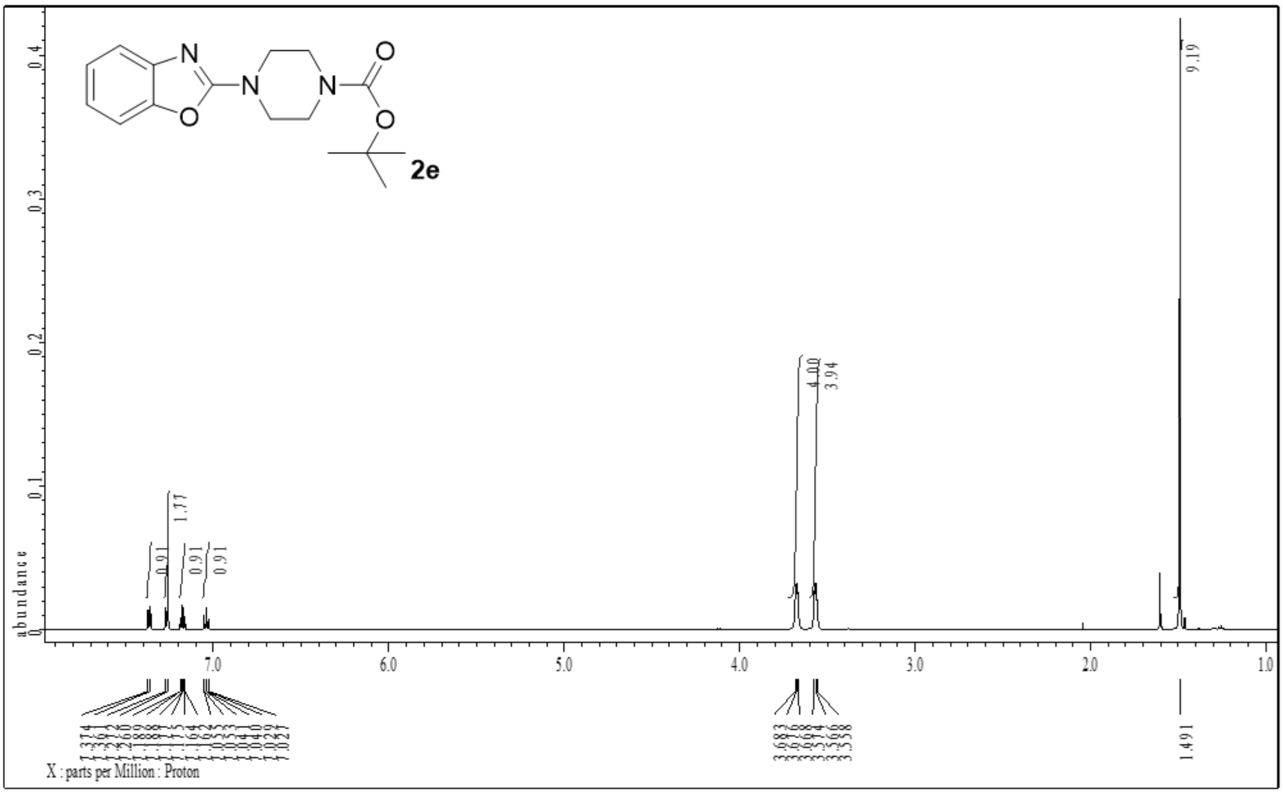

^{1}H NMR (600 MHz, $CDCl_3$) of *tert*-butyl 4-(benzoxazol-2-yl)piperazine-1-carboxylate (**2e**)

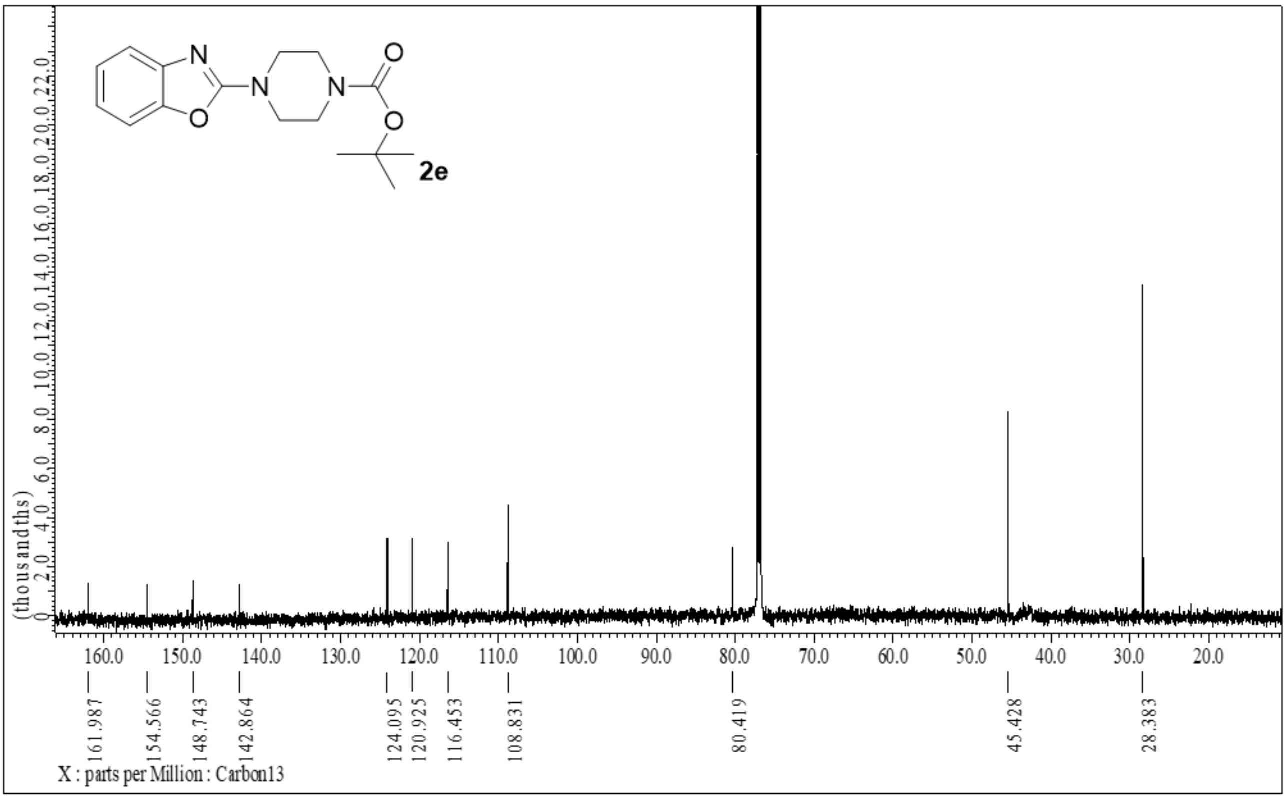

^{13}C NMR (151 MHz, $CDCl_3$) of *tert*-butyl 4-(benzoxazol-2-yl)piperazine-1-carboxylate (**2e**)

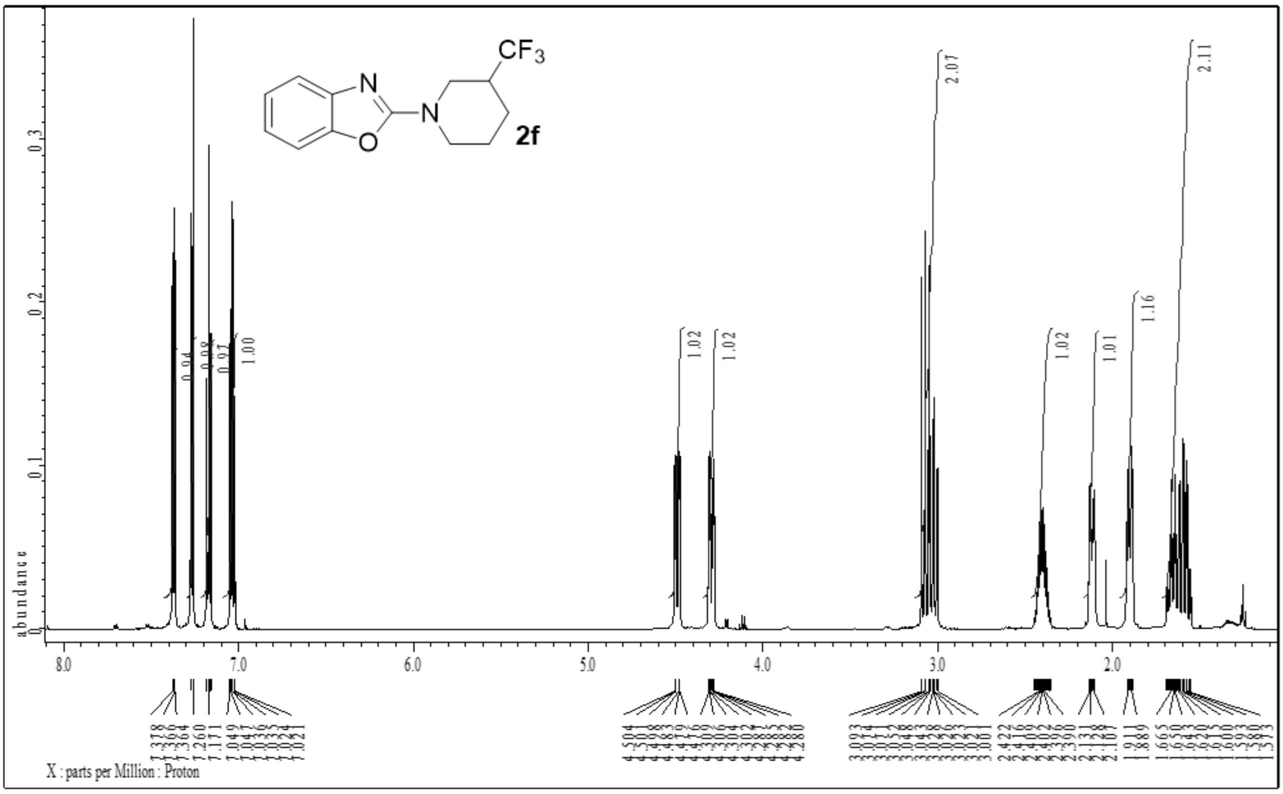

^{1}H NMR (600 MHz, $CDCl_3$) of 2-(3-(trifluoromethyl)piperidin-1-yl)benzoxazole (**2f**)

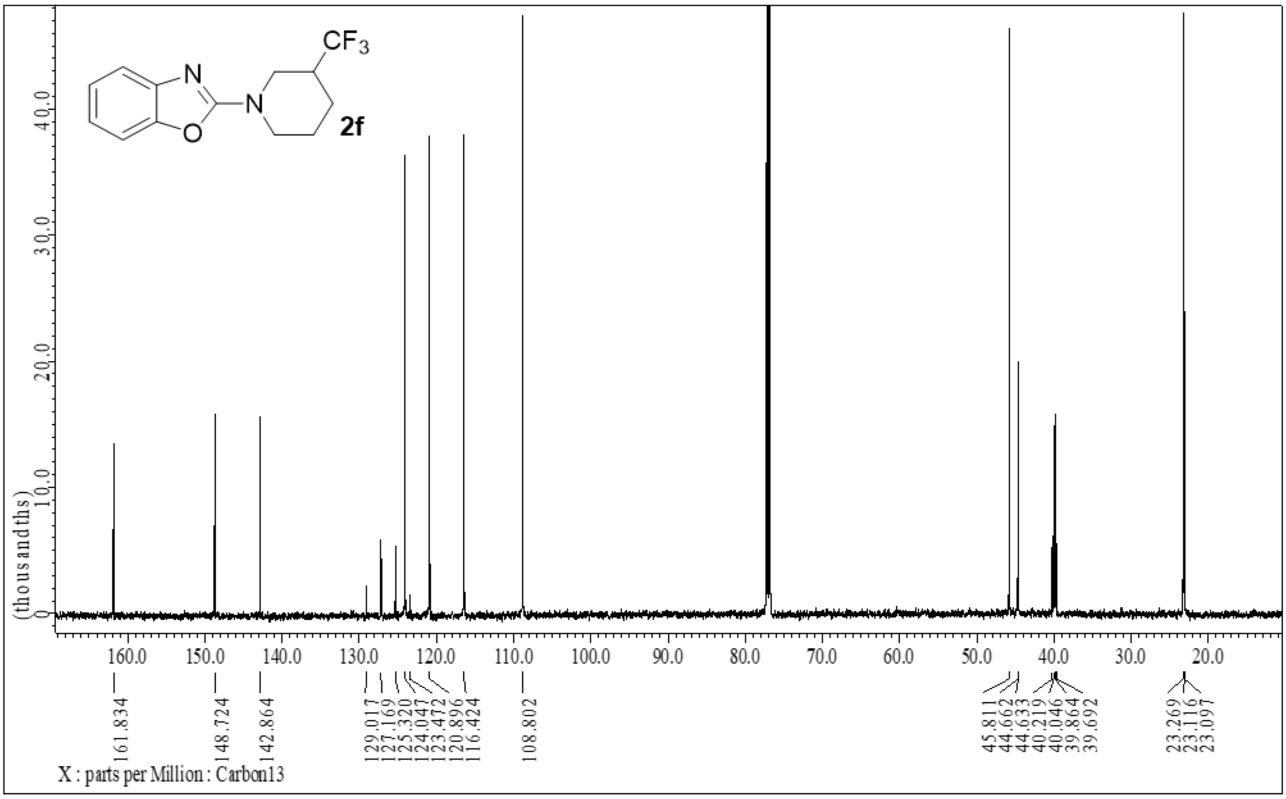

^{13}C NMR (151 MHz, $CDCl_3$) of 2-(3-(trifluoromethyl)piperidin-1-yl)benzoxazole (**2f**)

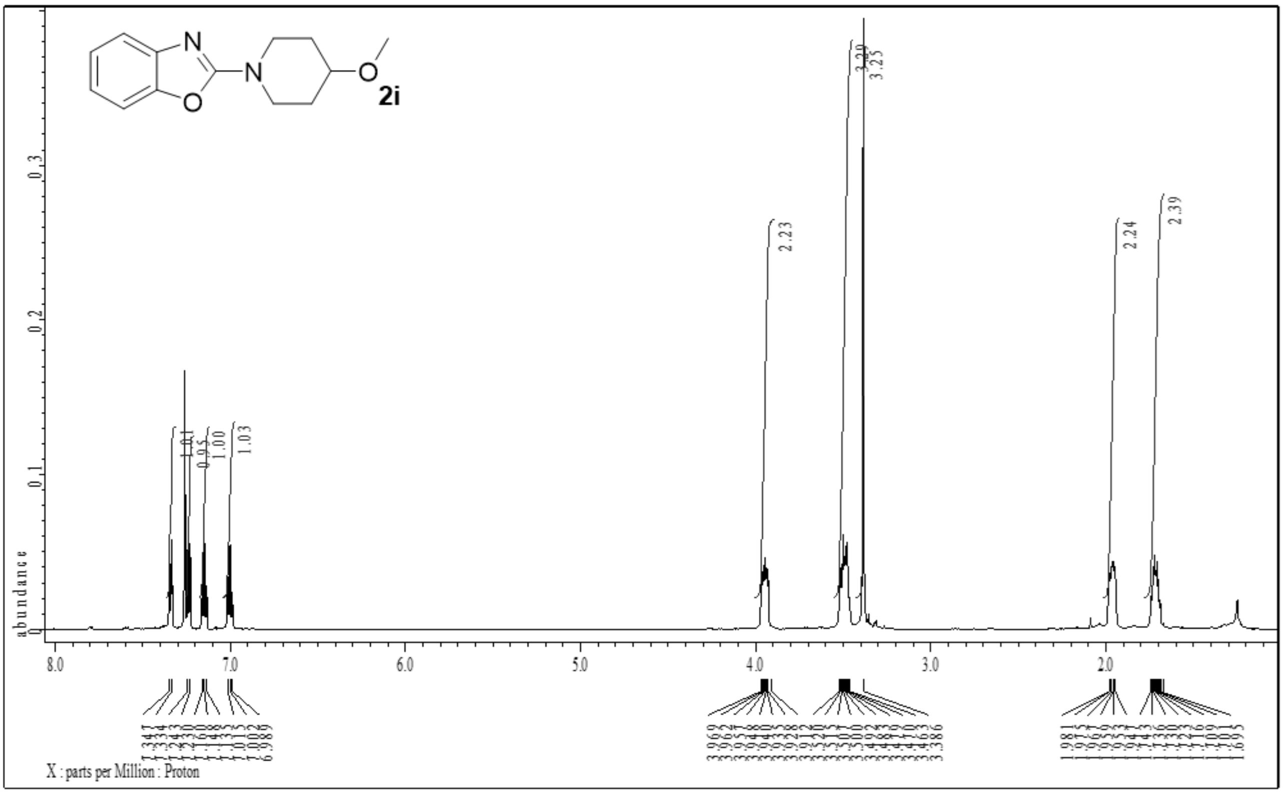

^{1}H NMR (600 MHz, $CDCl_3$) of 2-(4-methoxypiperidin-1-yl)benzoxazole (**2i**)

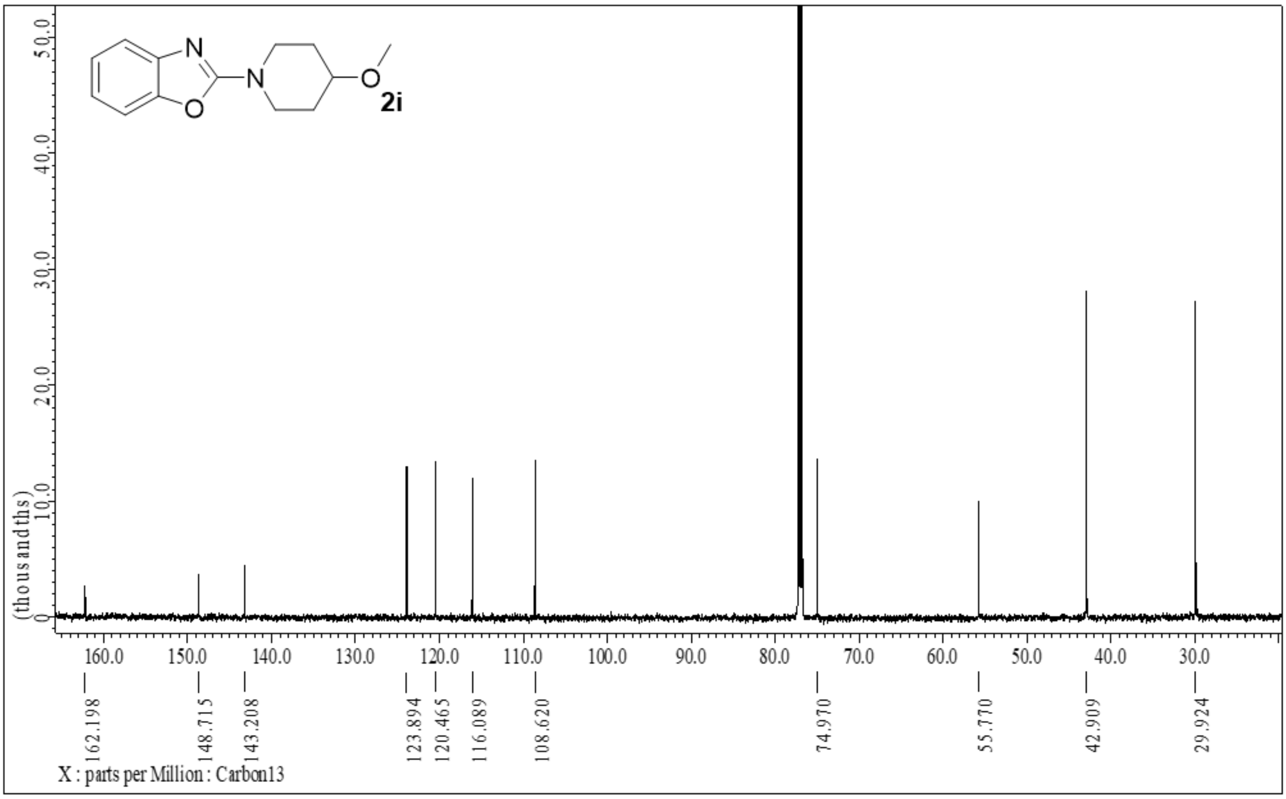

^{13}C NMR (151 MHz, $CDCl_3$) of 2-(4-methoxypiperidin-1-yl)benzoxazole (**2i**)

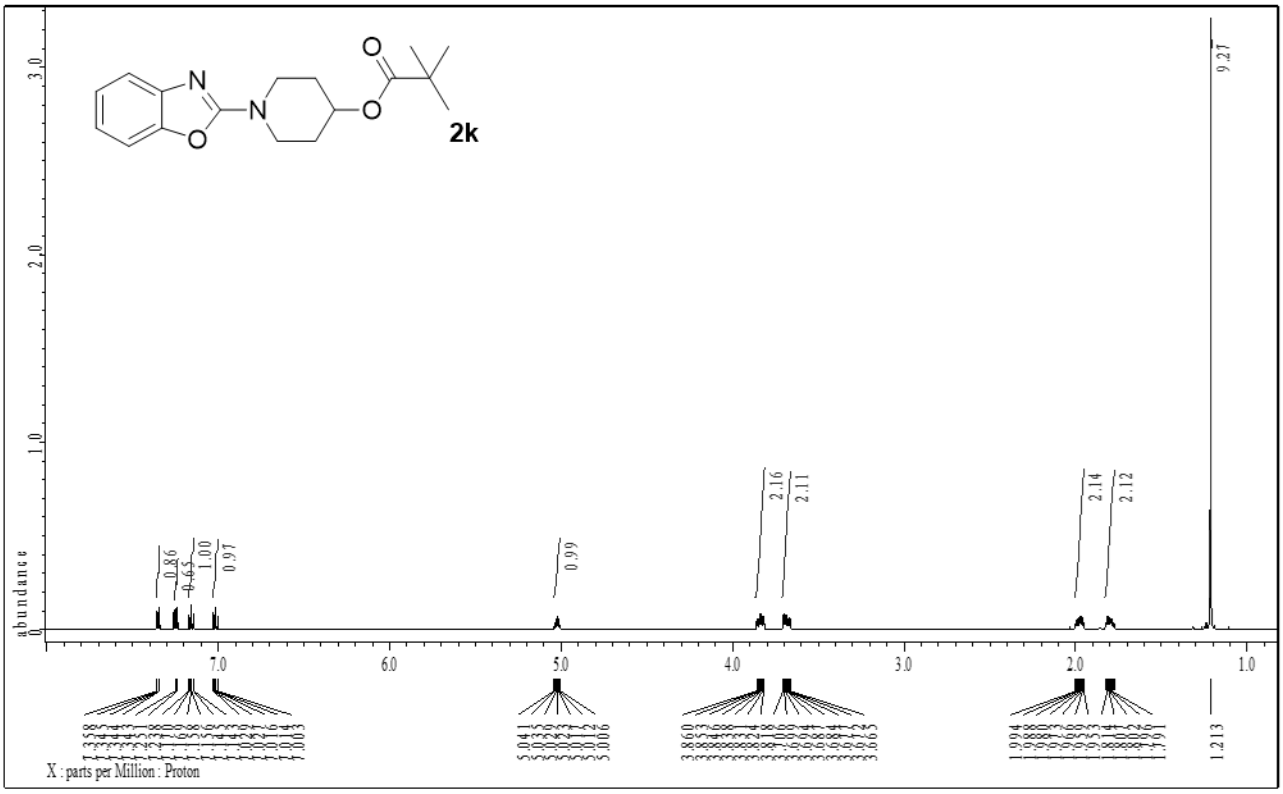

^{1}H NMR (600 MHz, $CDCl_3$) of 1-(benzoxazol-2-yl)2,2-dimethyl-propionic acid piperidin-4-yl ester (**2k**)

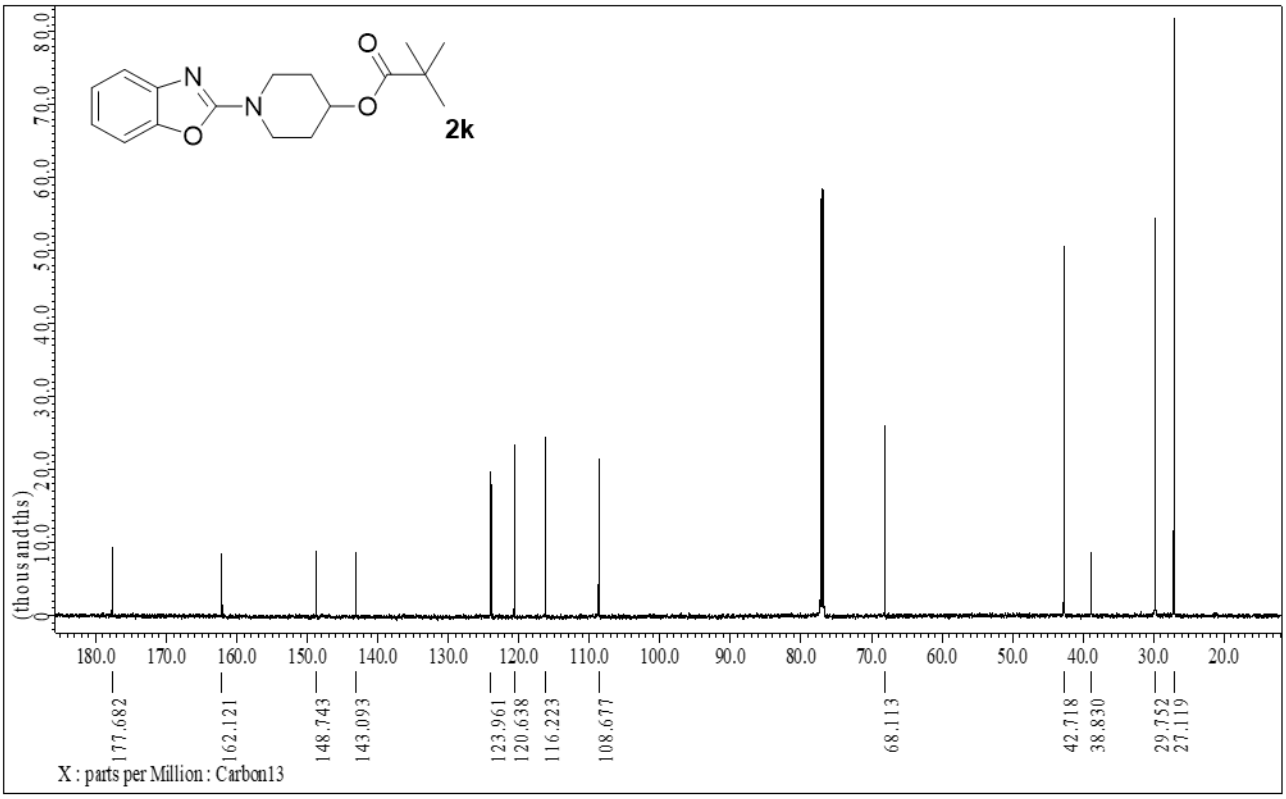

^{13}C NMR (151 MHz, $CDCl_3$) of 1-(benzoxazol-2-yl)-2,2-dimethylpropionic acid piperidin-4-yl ester (**2k**)

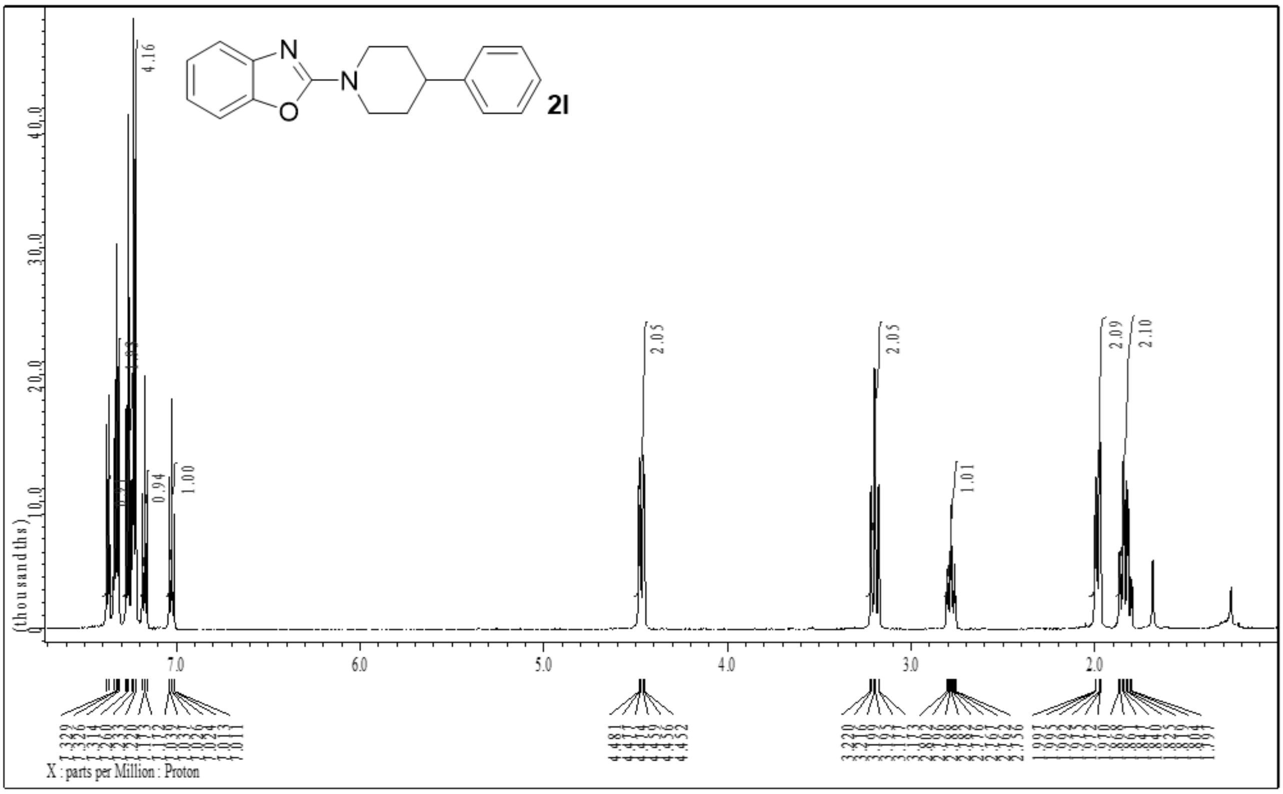

^{1}H NMR (600 MHz, $CDCl_3$) of 2-(4-phenylpiperidin-1-yl)benzoxazole (**2l**)

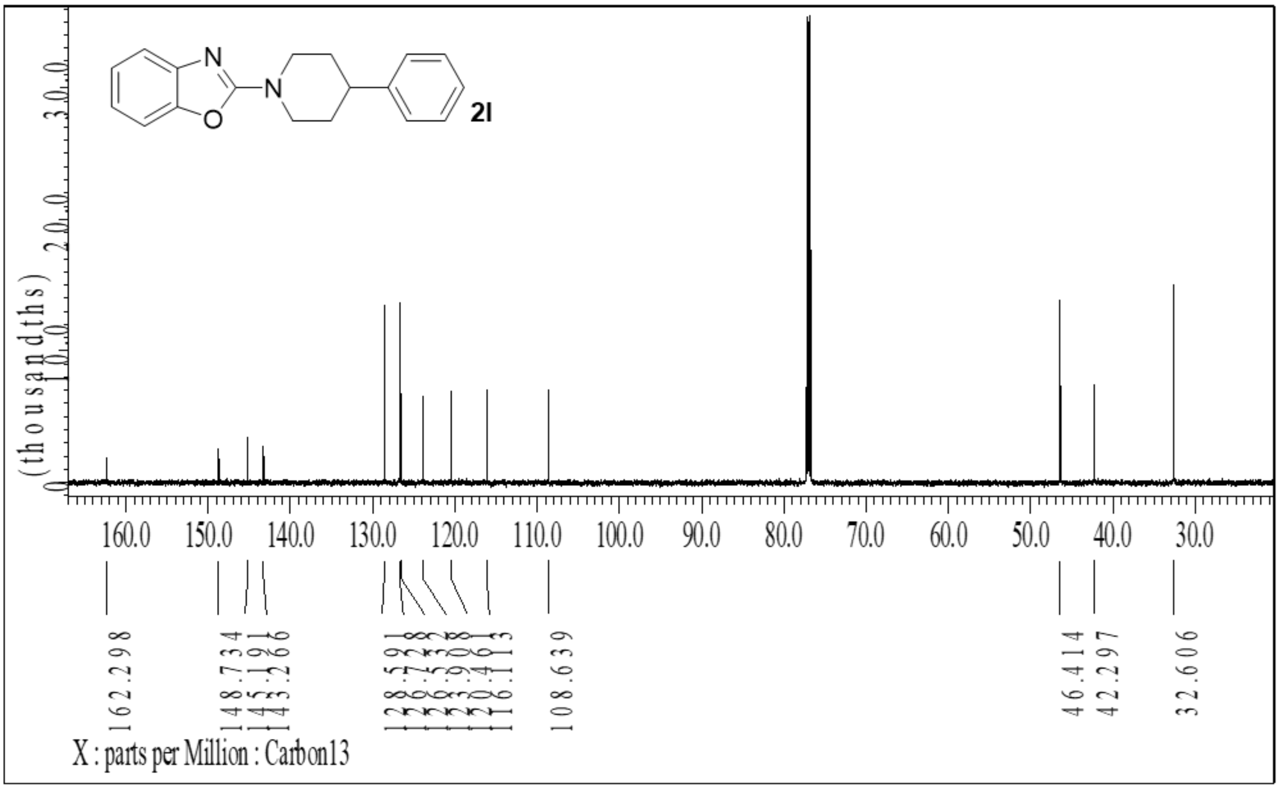

^{13}C NMR (151 MHz, $CDCl_3$) of 2-(4-phenylpiperidin-1-yl)benzoxazole (**2l**)

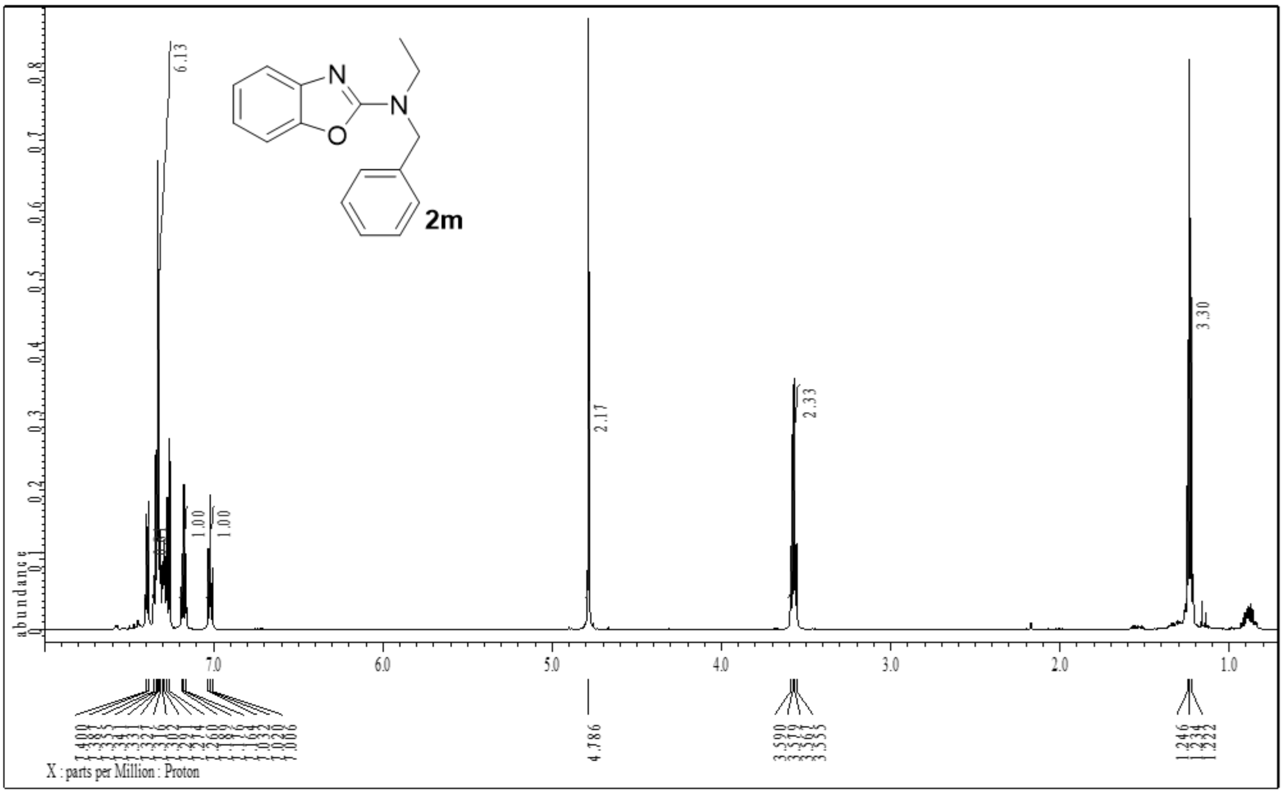

^{1}H NMR (600 MHz, $CDCl_3$) of *N*-benzyl-*N*-ethylbenzoxazol-2-amine (**2m**)

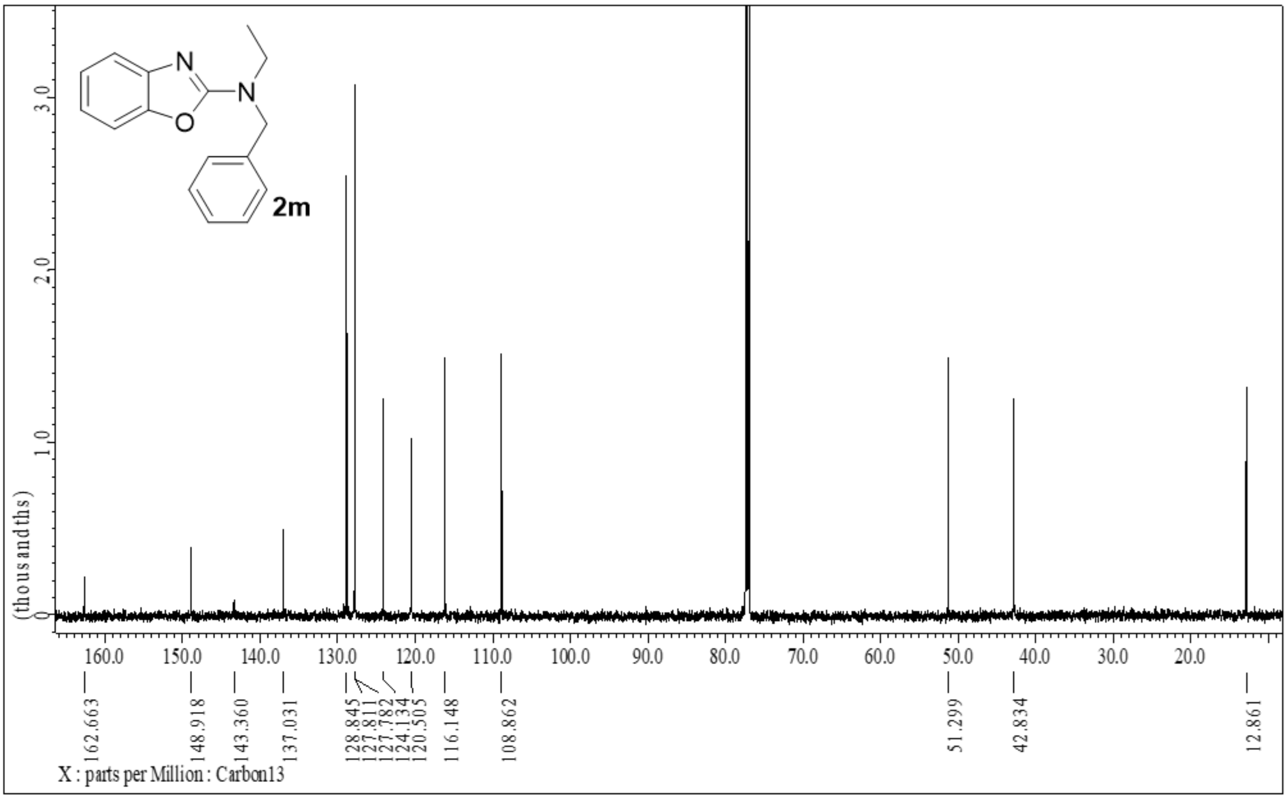

^{13}C NMR (151 MHz, $CDCl_3$) of *N*-benzyl-*N*-ethylbenzoxazol-2-amine (**2m**)

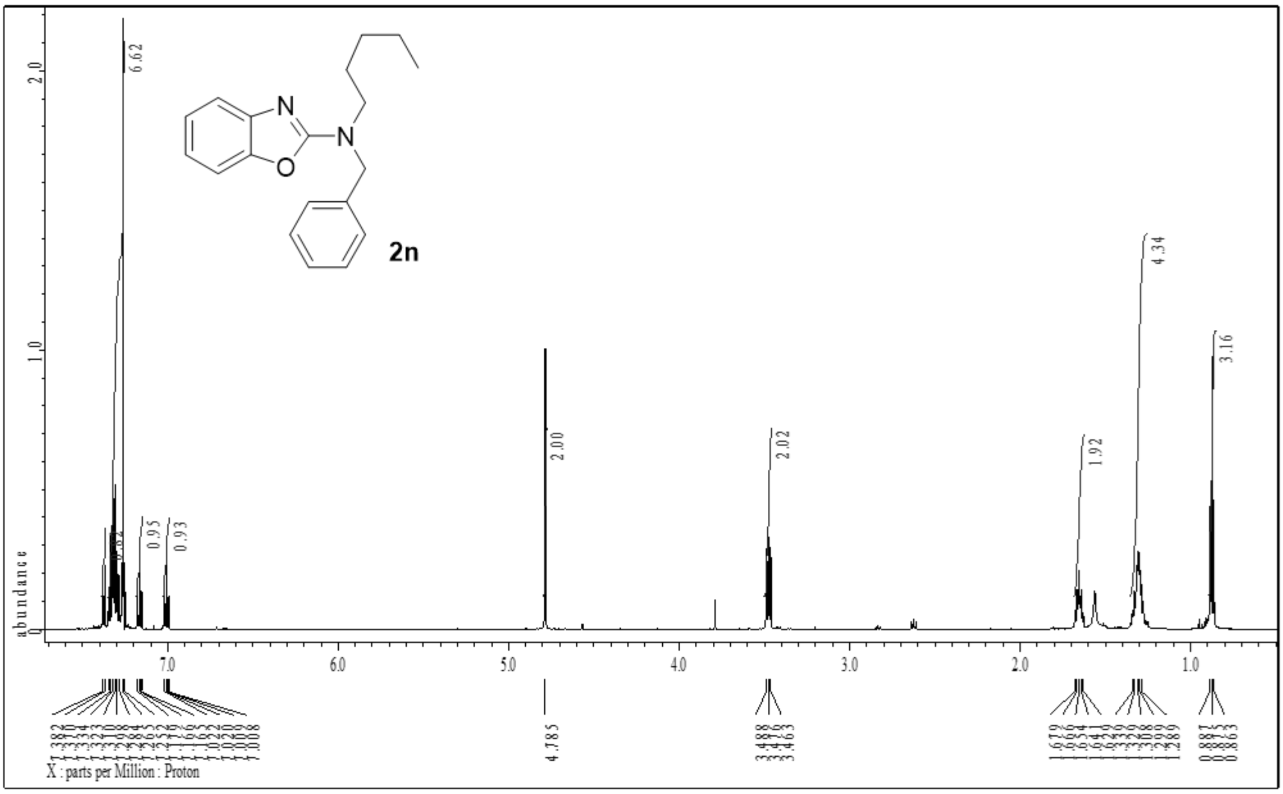

^{1}H NMR (600 MHz, $CDCl_3$) of *N*-benzyl-*N*-pentylbenzoxazol-2-amine (**2n**)

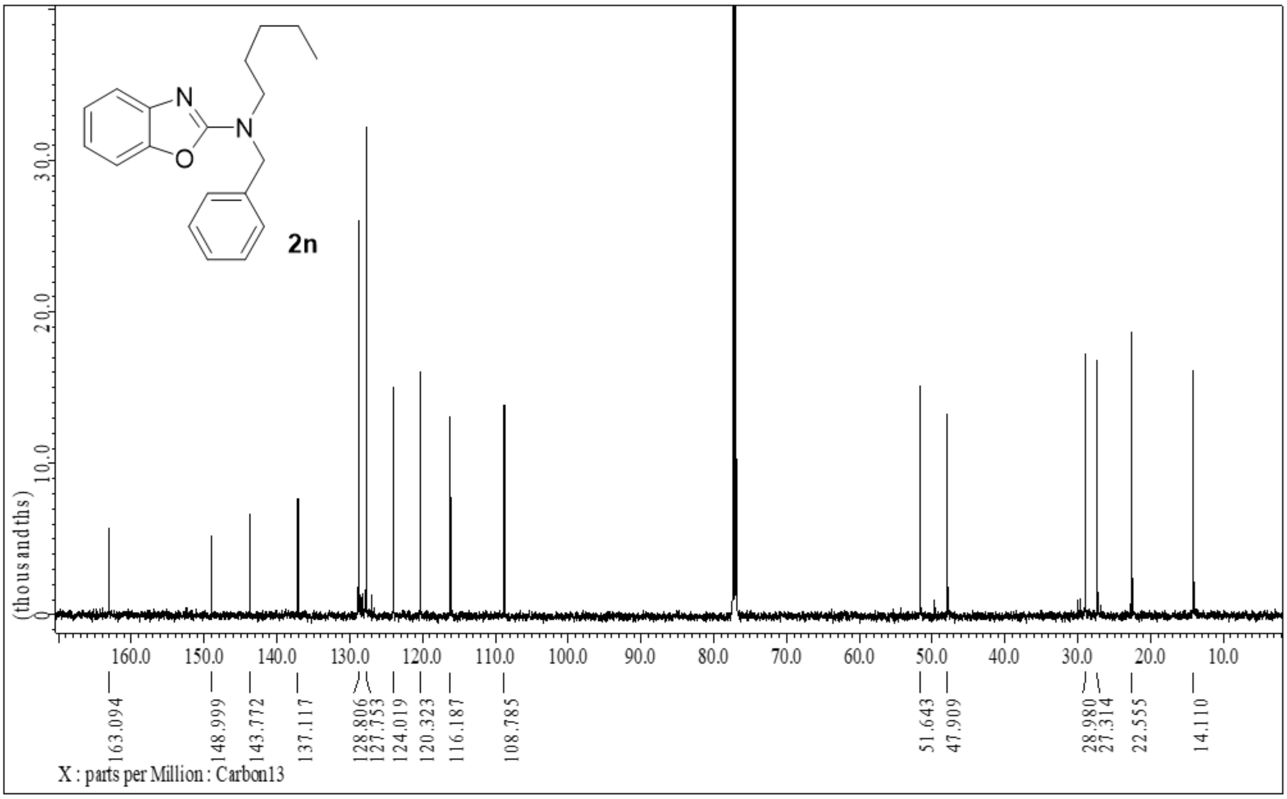

^{13}C NMR (151 MHz, $CDCl_3$) of *N*-benzyl-*N*-pentylbenzoxazol-2-amine (**2n**)

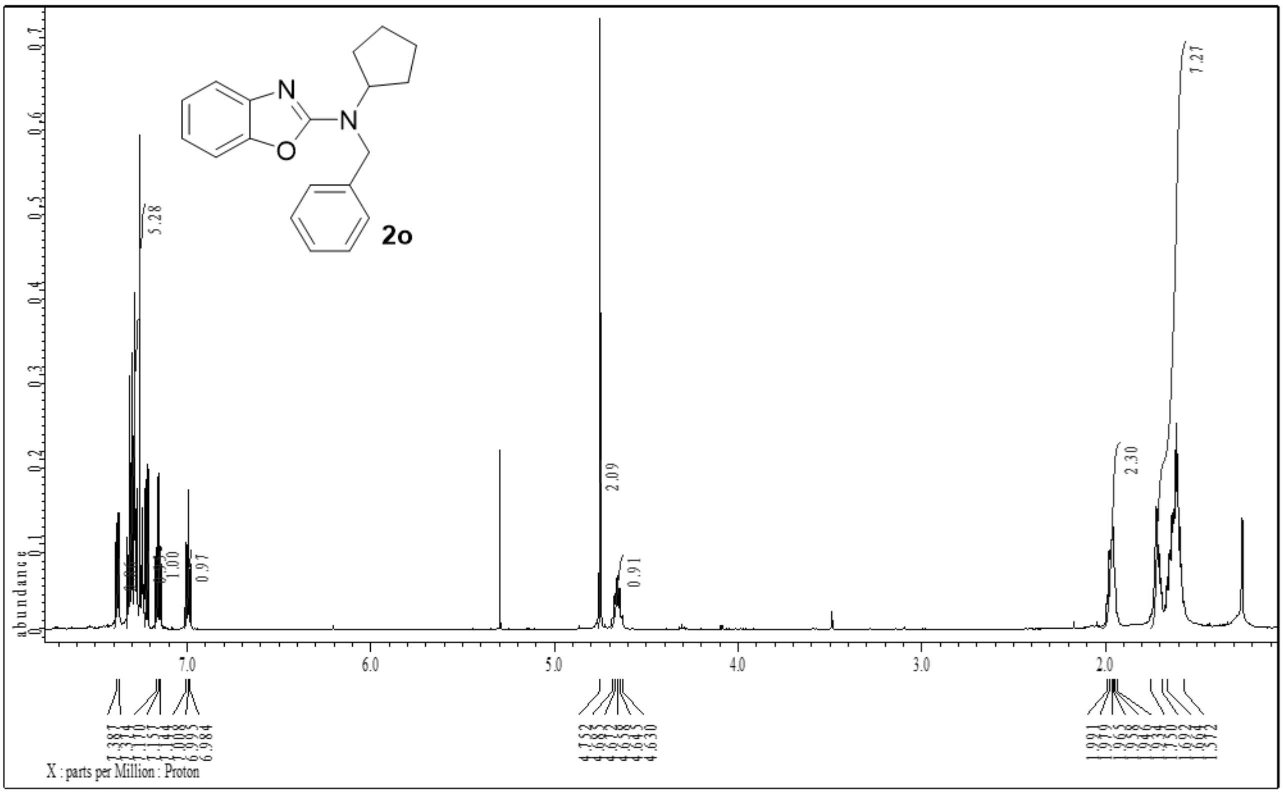

^{1}H NMR (600 MHz, $CDCl_3$) of *N*-benzyl-*N*-cyclopentylbenzoxazol-2-amine (**2o**)

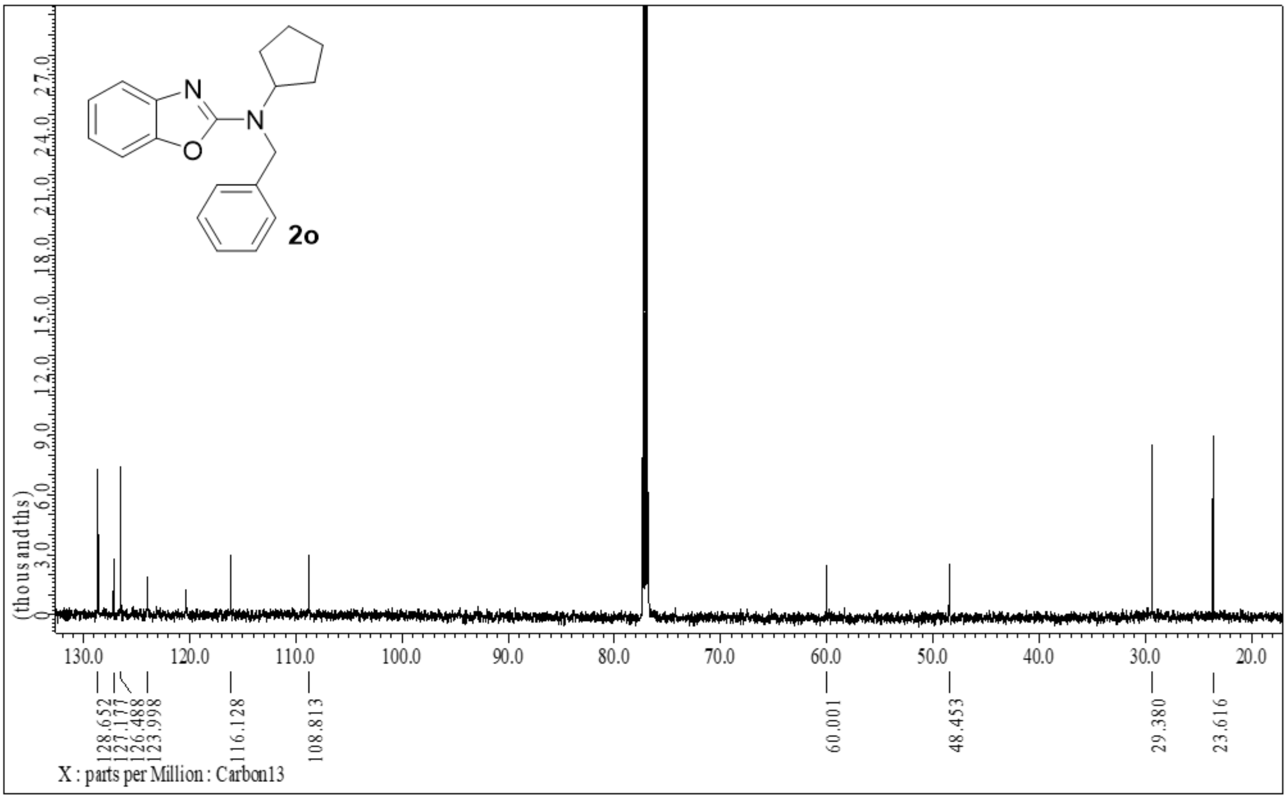

^{13}C NMR (151 MHz, $CDCl_3$) of *N*-benzyl-*N*-cyclopentylbenzoxazol-2-amine (**2o**)

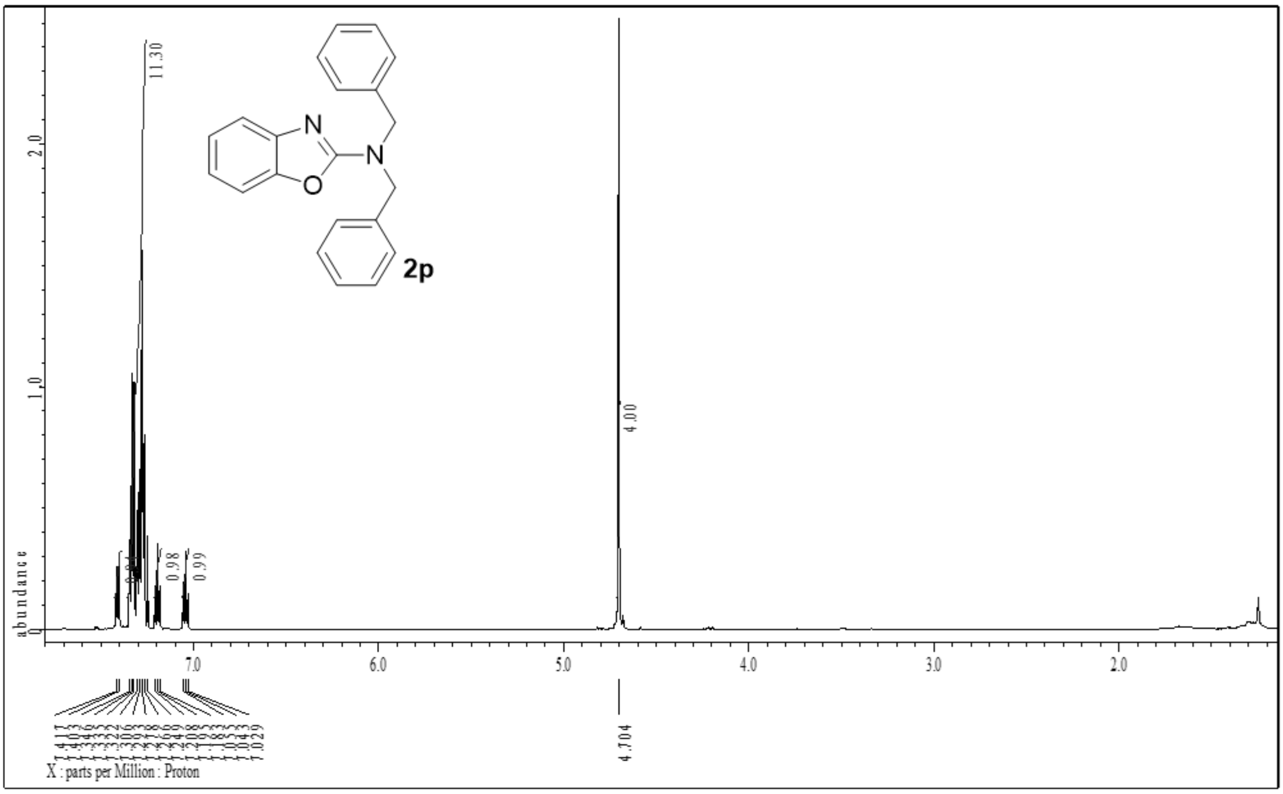

^{1}H NMR (600 MHz, $CDCl_3$) of *N*,*N*-dibenzylbenzoxazol-2-amine (**2p**)

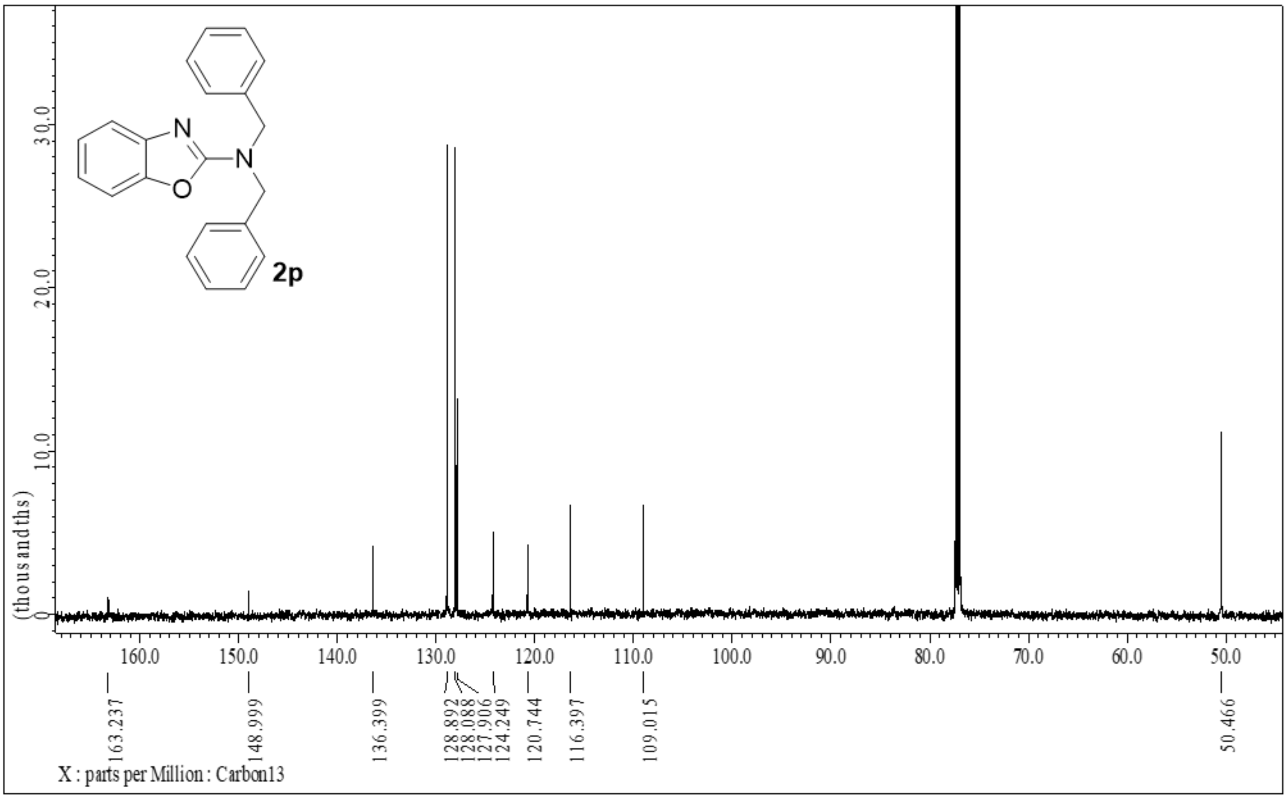

^{13}C NMR (151 MHz, $CDCl_3$) of *N*,*N*-dibenzylbenzoxazol-2-amine (**2p**)

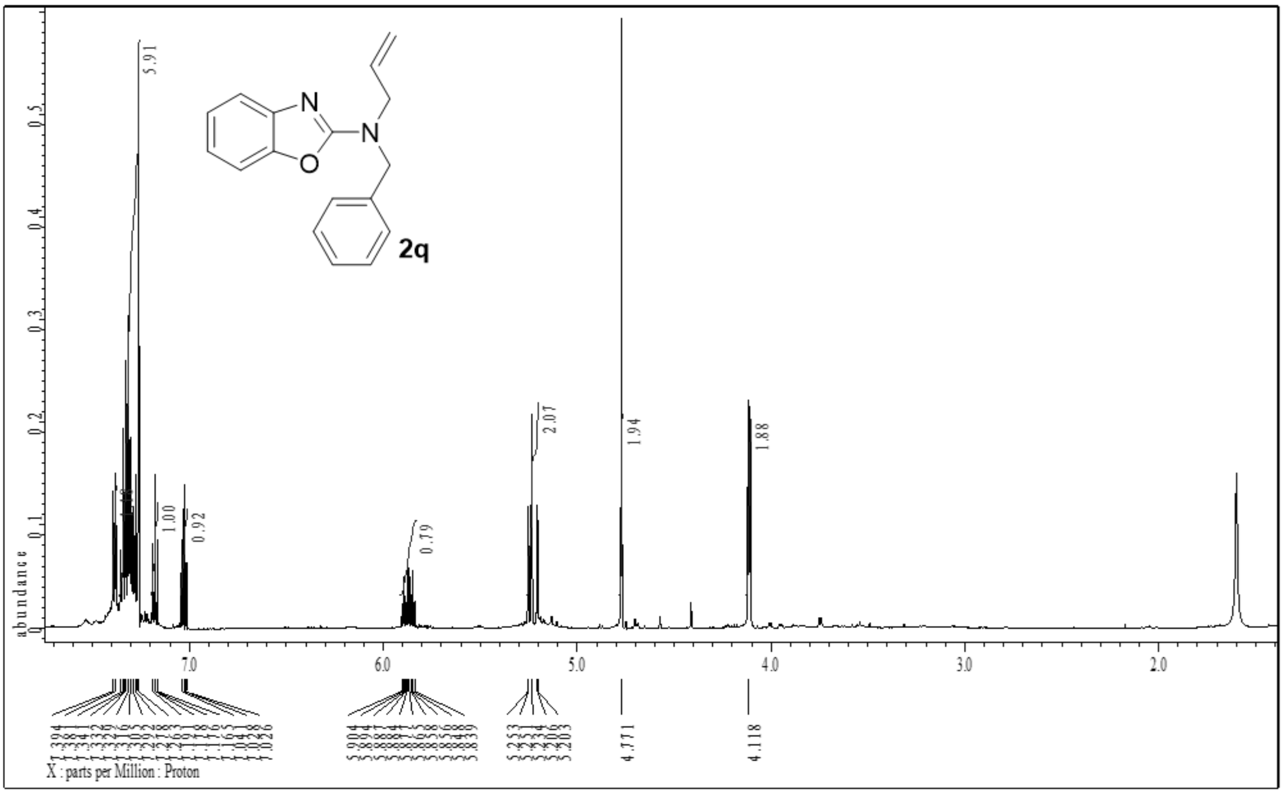

^{1}H NMR (600 MHz, $CDCl_3$) of *N*-allyl-*N*-benzylbenzoxazol-2-amine (**2q**)

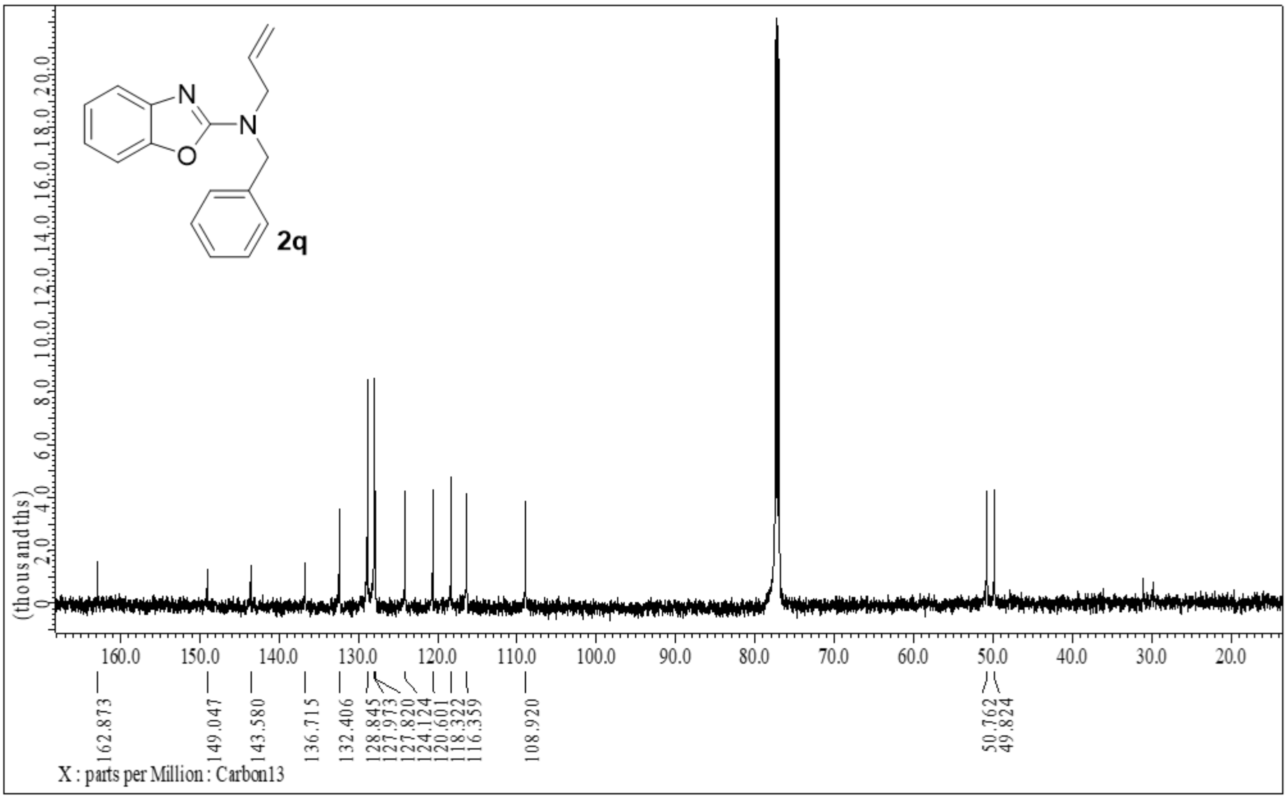

^{13}C NMR (151 MHz, $CDCl_3$) of *N*-allyl-*N*-benzylbenzoxazol-2-amine (**2q**)

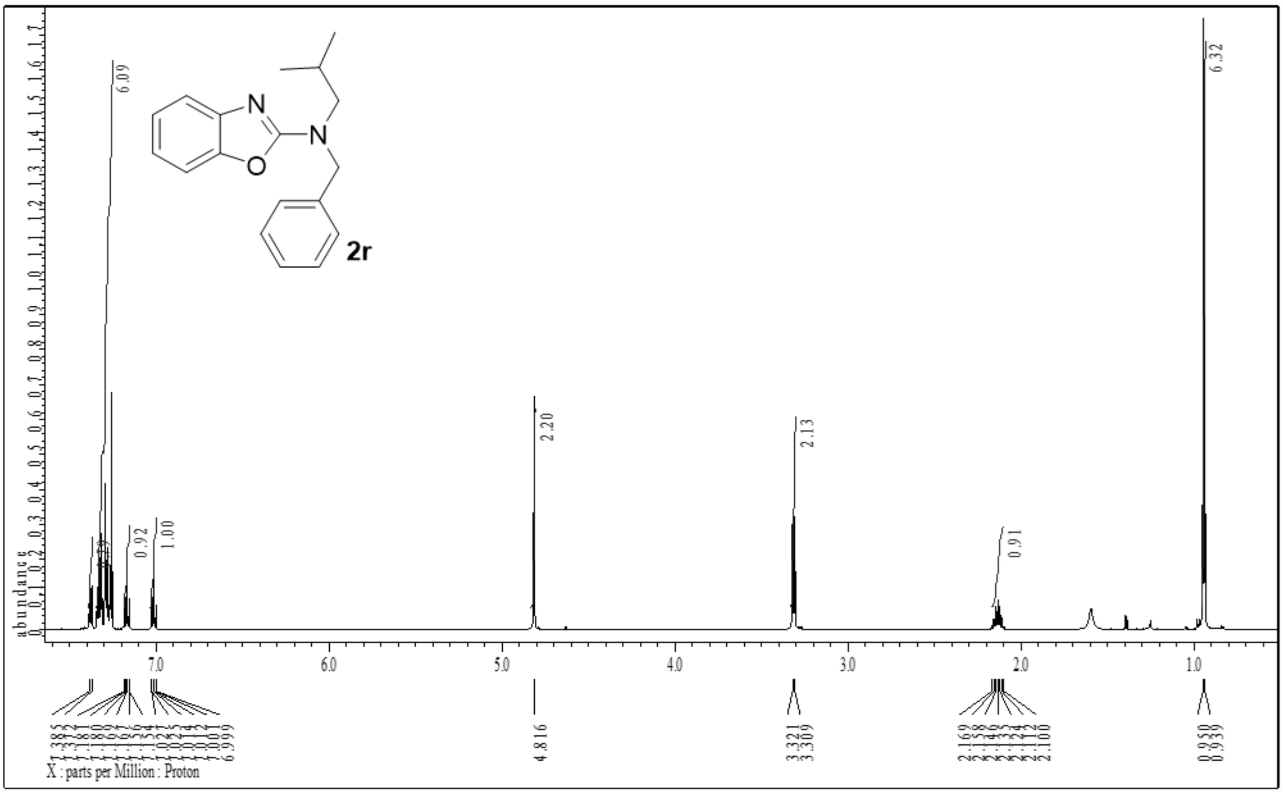

^{1}H NMR (600 MHz, $CDCl_3$) of *N*-benzyl-*N*-isobutylbenzoxazol-2-amine (**2r**)

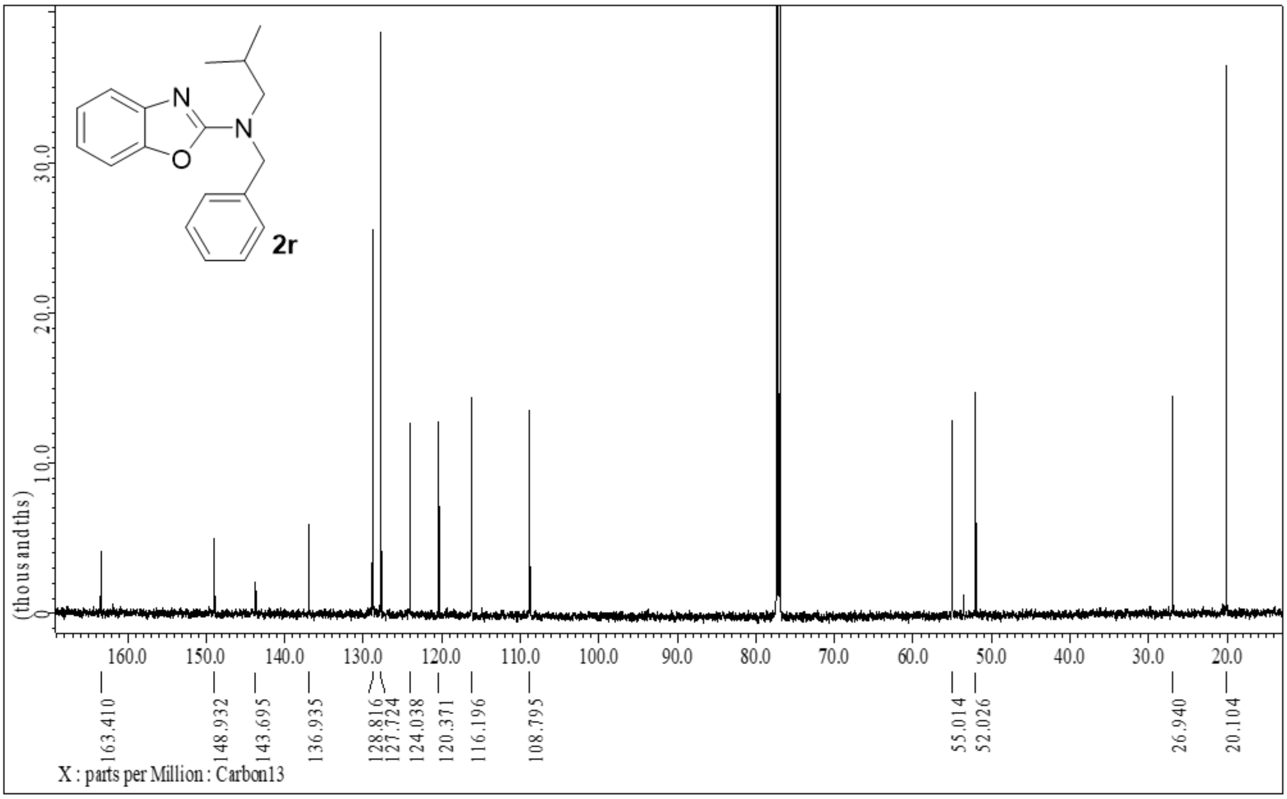

^{13}C NMR (151 MHz, $CDCl_3$) of *N*-allyl-*N*-benzylbenzoxazol-2-amine (**2r**)

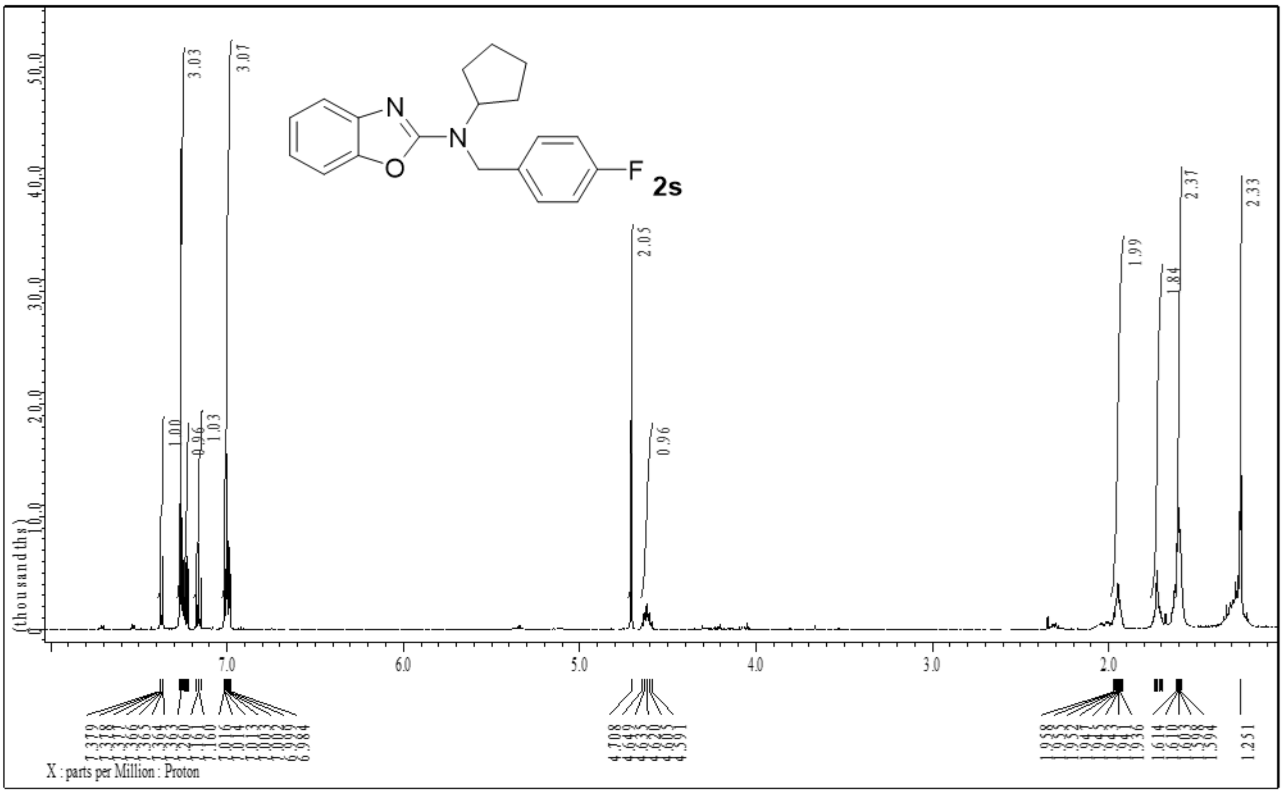

^{1}H NMR (600 MHz, $CDCl_3$) of *N*-cyclopentyl-*N*-(4-fluorobenzyl)benzoxazol-2-amine (**2s**)

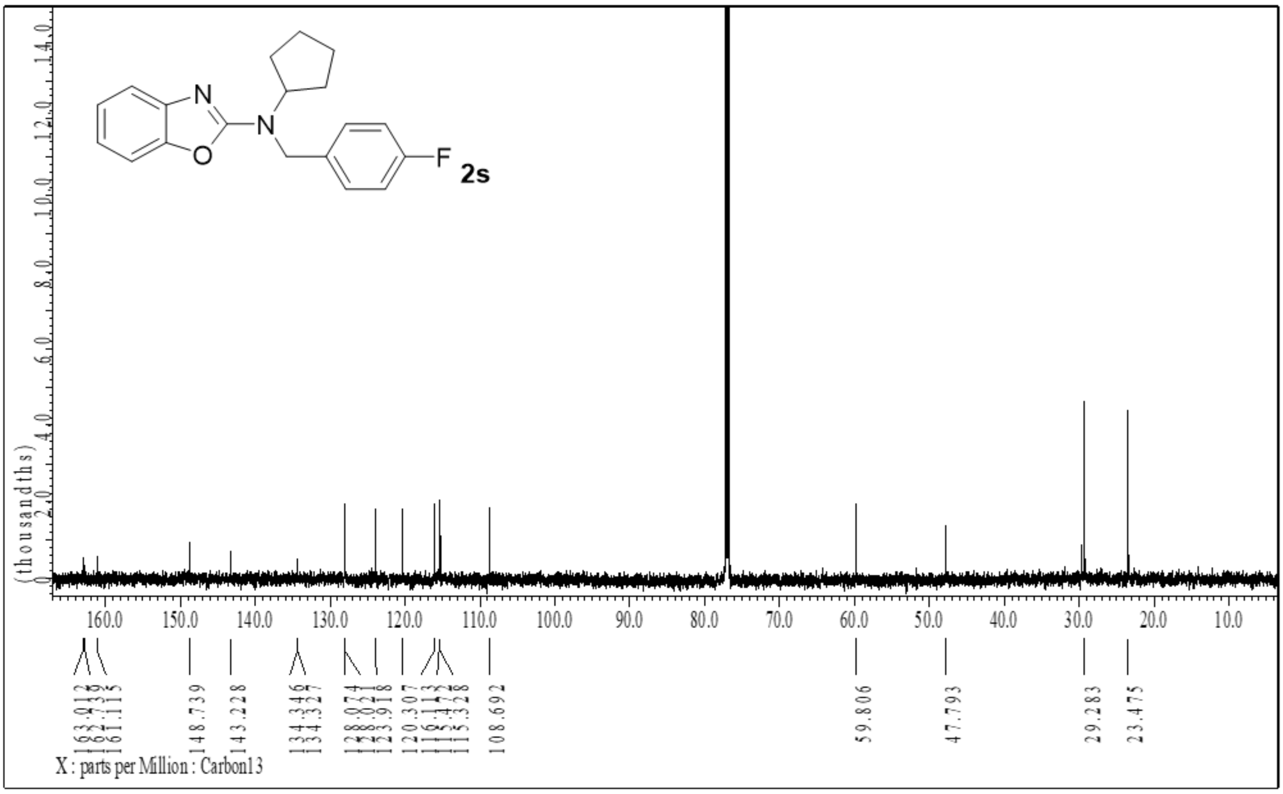

^{13}C NMR (151 MHz, $CDCl_3$) of *N*-cyclopentyl-*N*-(4-fluorobenzyl)benzoxazol-2-amine (**2s**)

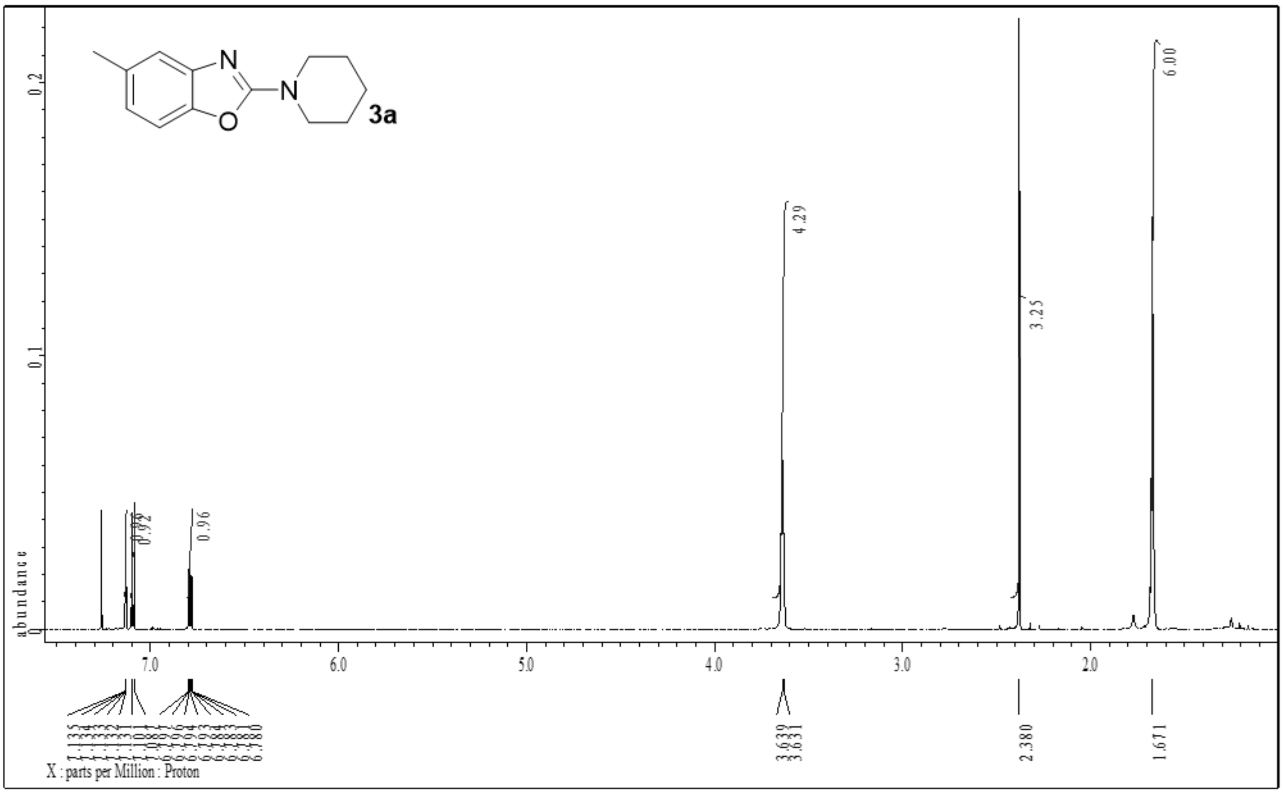

^{1}H NMR (600 MHz, $CDCl_3$) of 5-methyl-2-(piperidin-1-yl)benzoxazole (**3a**)

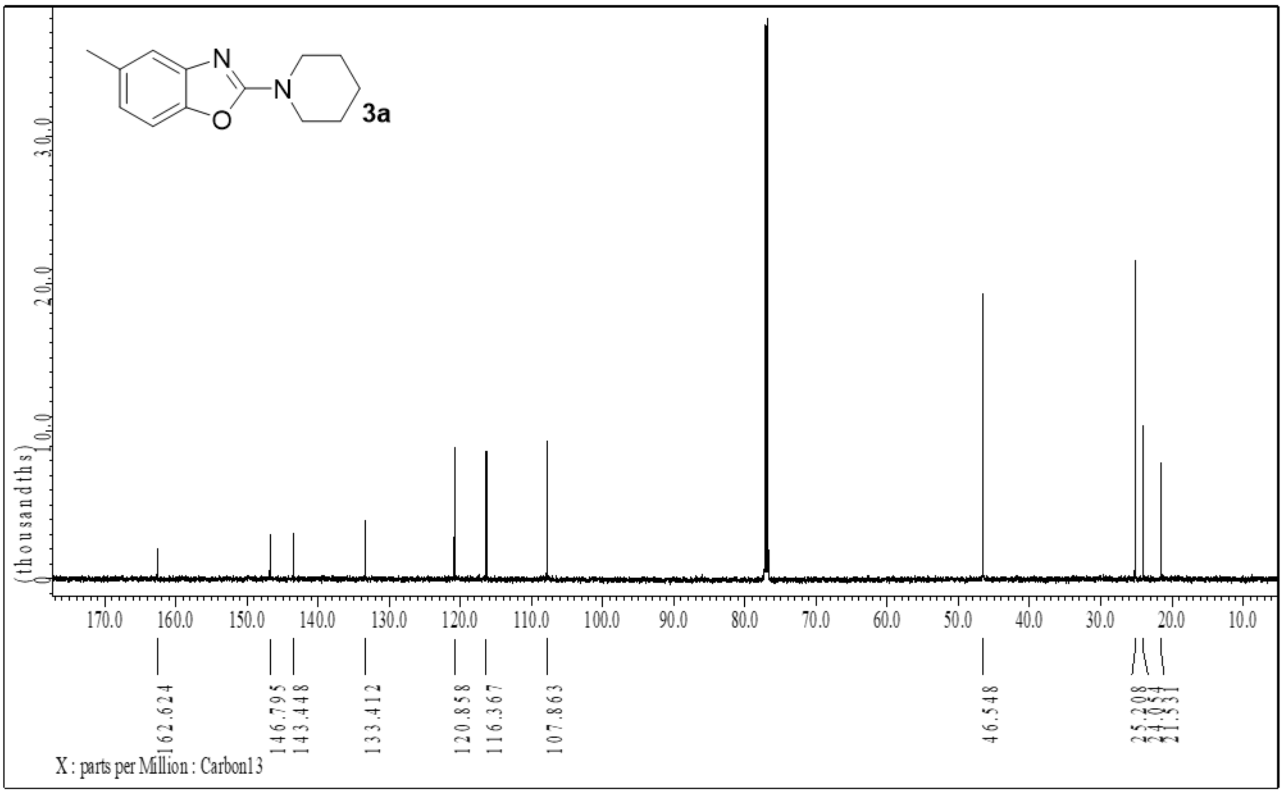

^{13}C NMR (151 MHz, $CDCl_3$) of 5-methyl-2-(piperidin-1-yl)benzoxazole (**3a**)

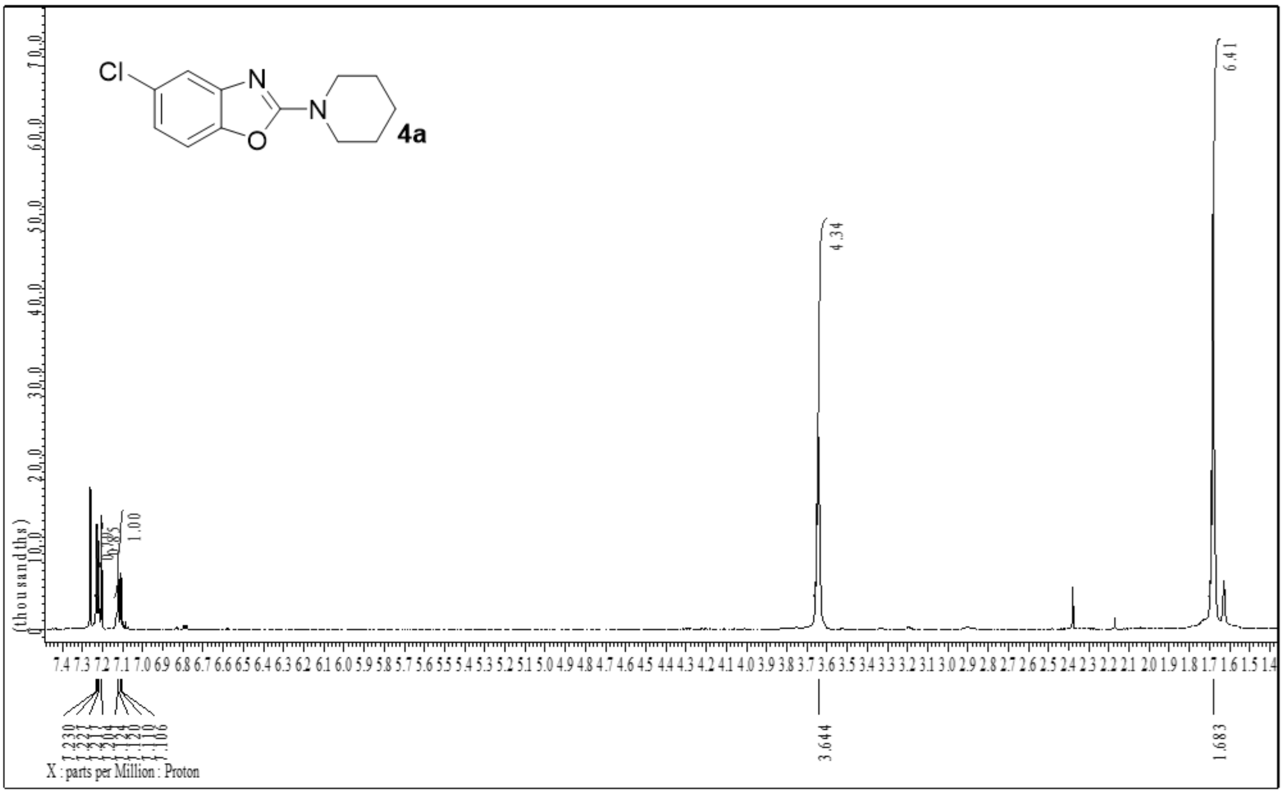

^{1}H NMR (600 MHz, $CDCl_3$) of 6-chloro-2-(piperidin-1-yl)benzoxazole (**4a**)

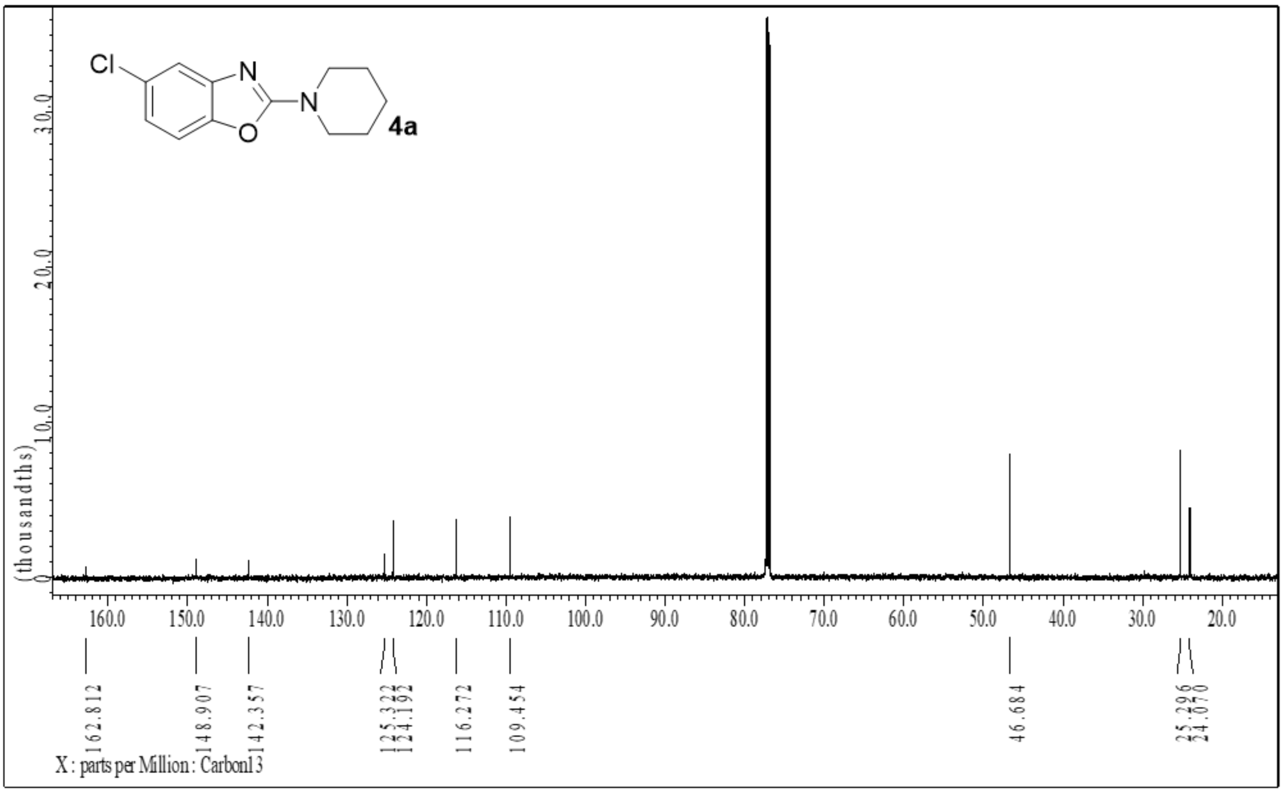

^{13}C NMR (151 MHz, $CDCl_3$) of 6-chloro-2-(piperidin-1-yl)benzoxazole (**4a**)

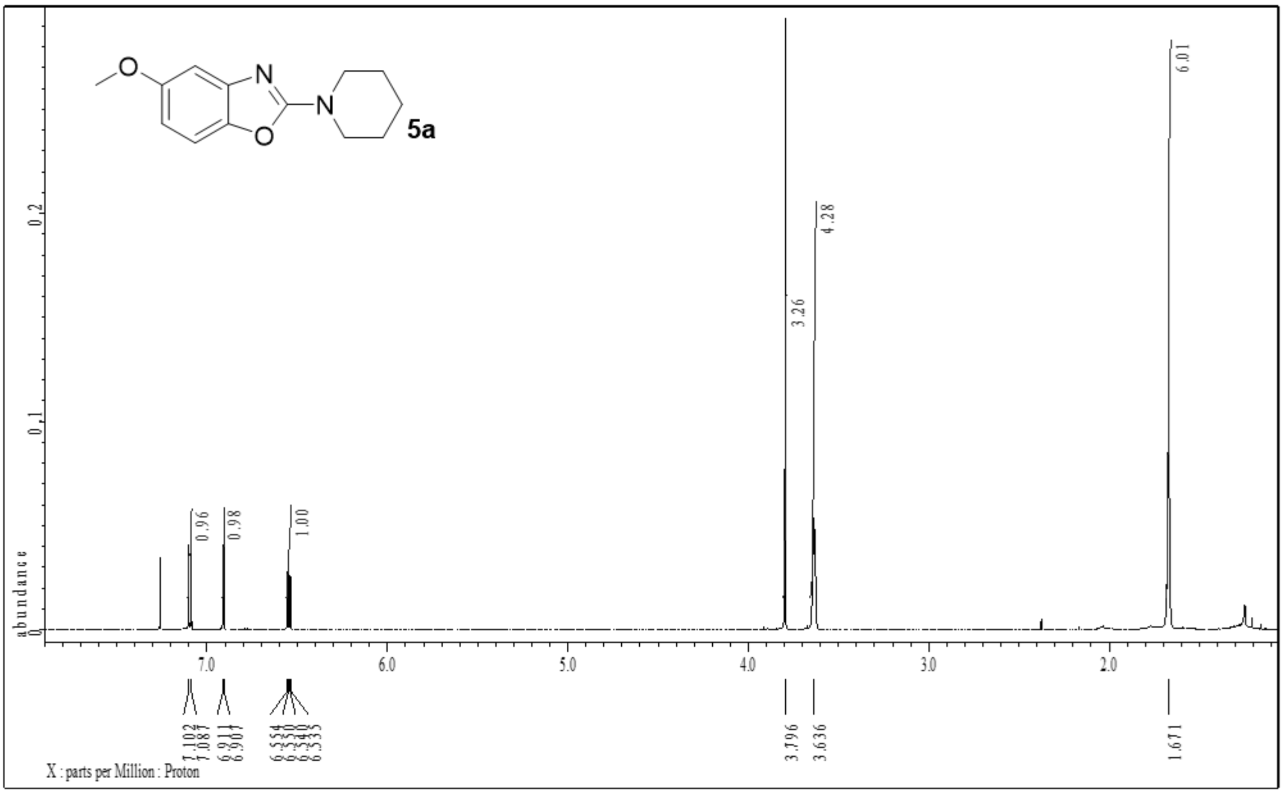

^{1}H NMR (600 MHz, $CDCl_3$) of 5-methoxy-2-(piperidin-1-yl)benzoxazole (**5a**)

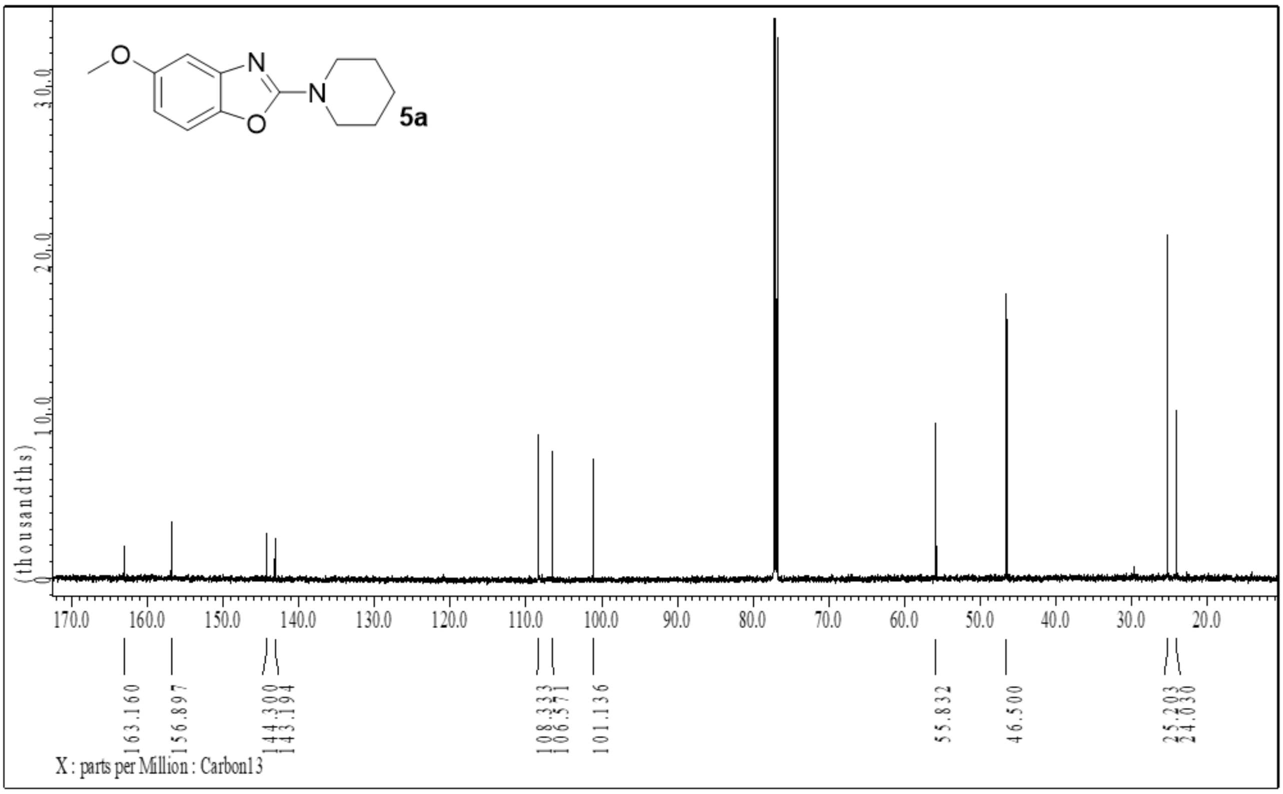

^{13}C NMR (151 MHz, $CDCl_3$) of 5-methoxy-2-(piperidin-1-yl)benzoxazole (**5a**)

MS spectra

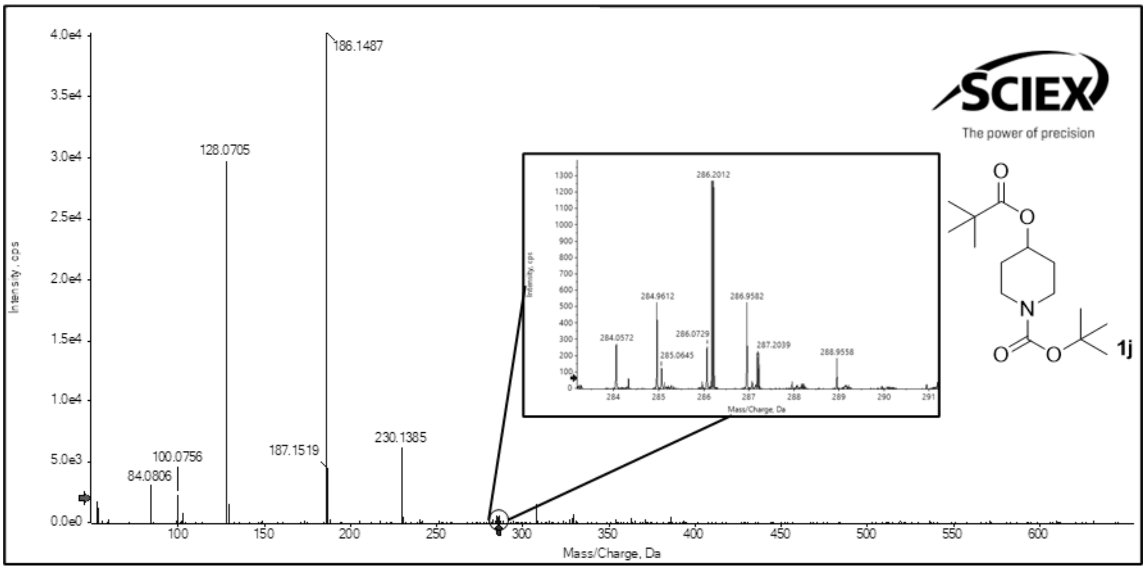

HR-ESI-MS of *tert*-butyl 4-(2,2-dimethylpropanoyloxy)piperidine-1-carboxylate (**1j**)

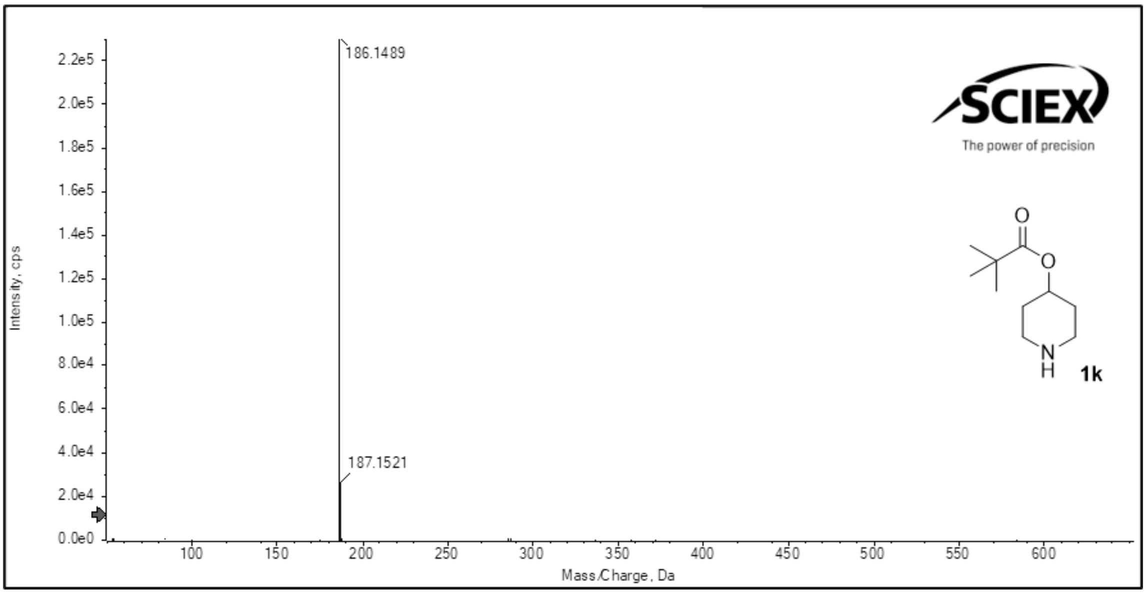

HR-ESI-MS of 2,2-dimethylpropionic acid piperidin-4-yl ester (**1k**)

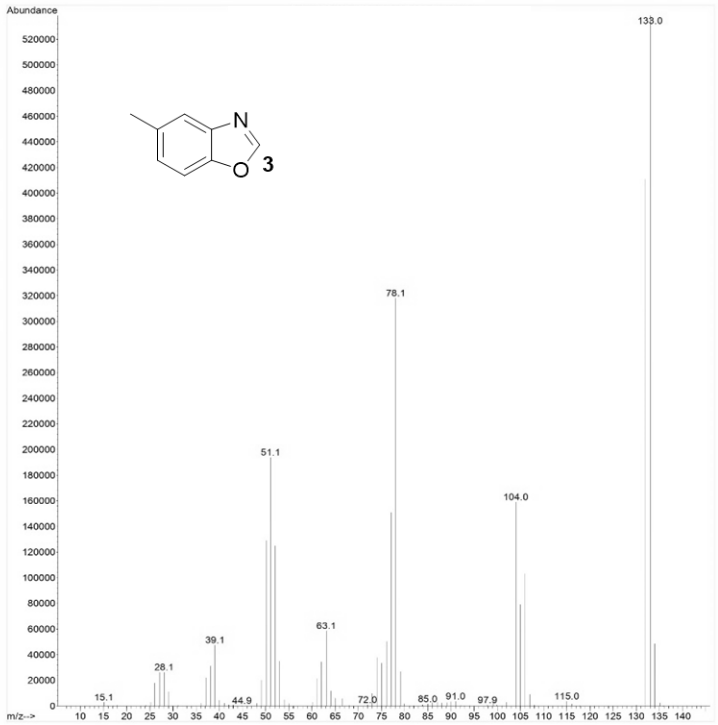

EI-MS of 5-methylbenzoxazole (**3**)

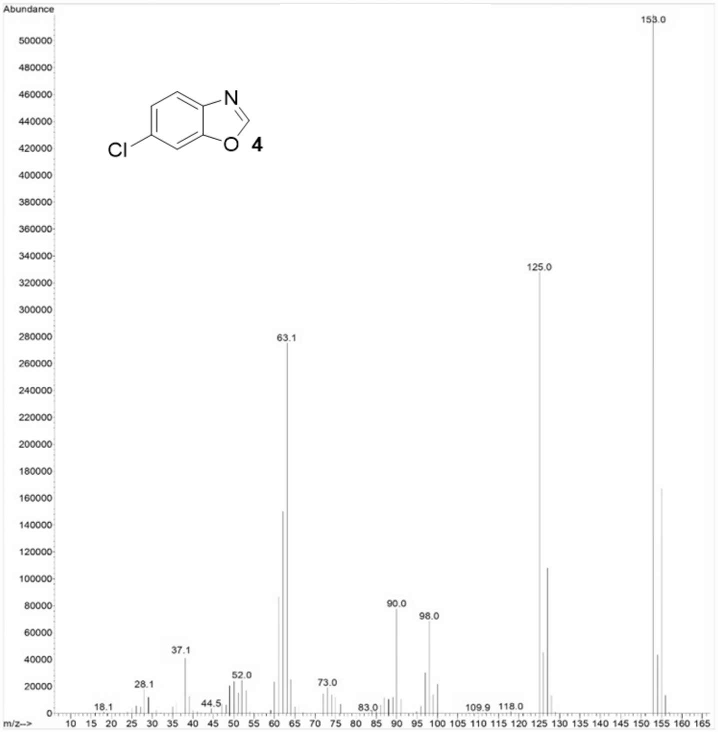

EI-MS of 6-chlorobenzoxazole (**4**)

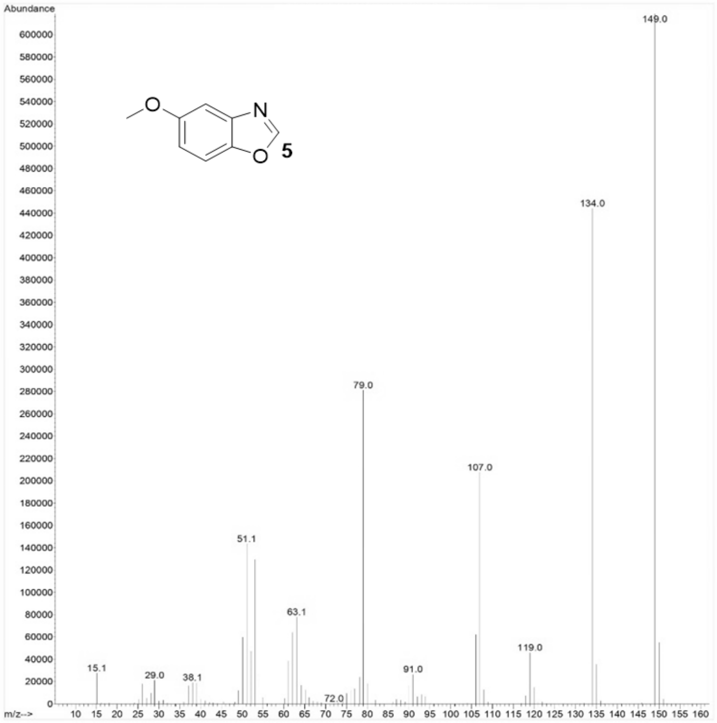

EI-MS of 5-methoxybenzoxazole (**5**)

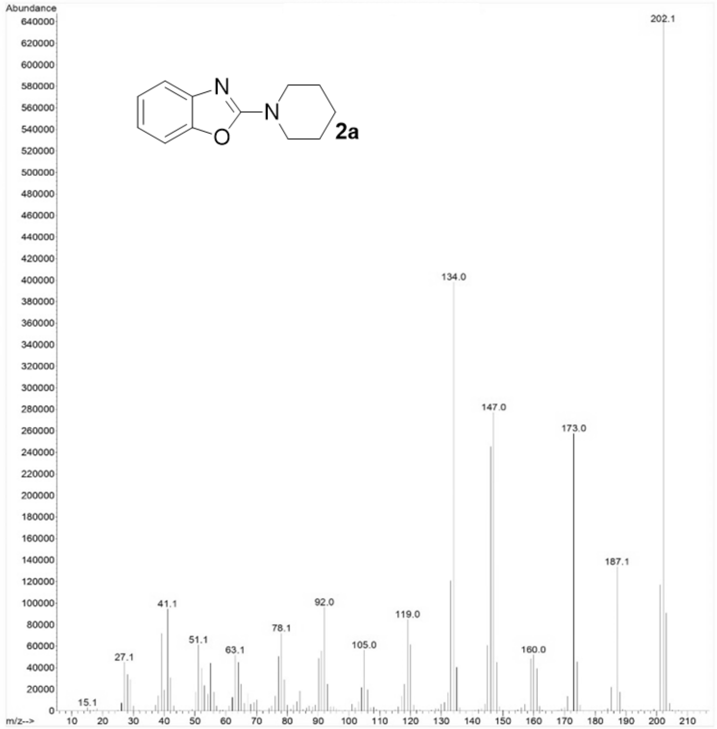

EI-MS of 2-(piperidin-1-yl)benzoxazole (**2a**)

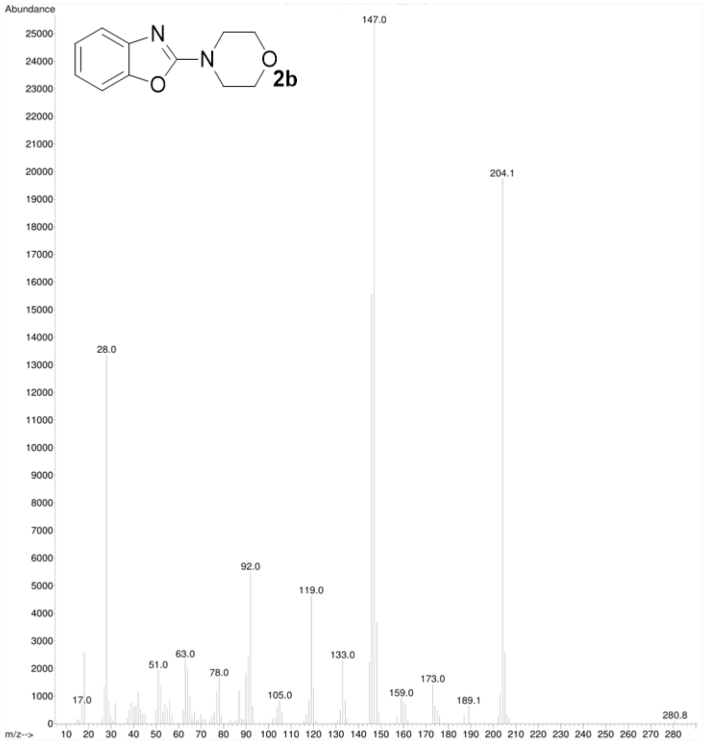

EI-MS of 2-(4-morpholinyl)benzoxazole (**2b**)

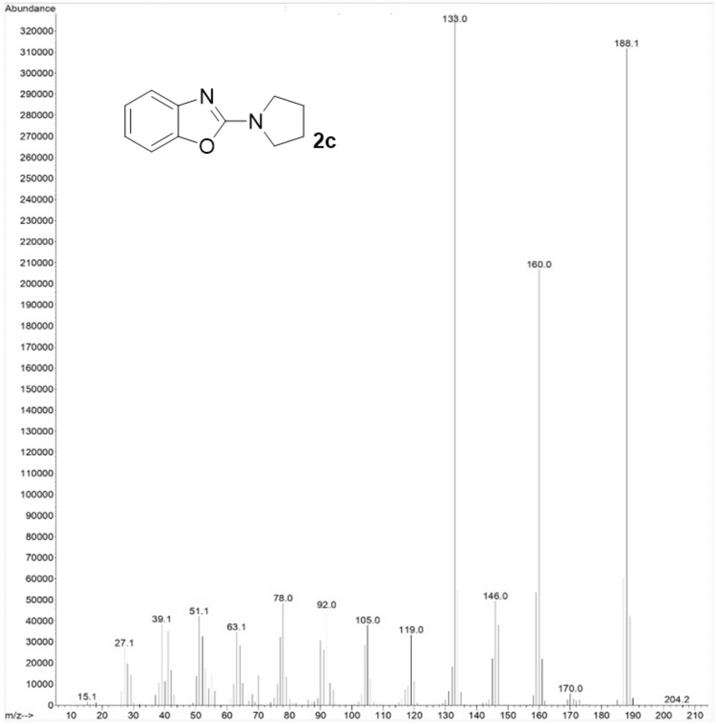

EI-MS of 2-(pyrrolidin-1-yl)benzoxazole (**2c**)

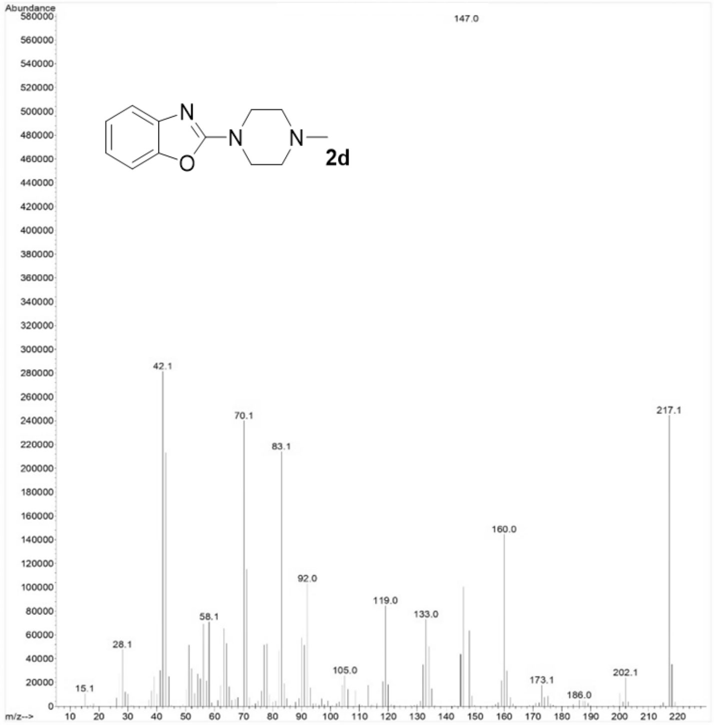

EI-MS of 2-(4-methyl-1-piperazinyl)benzoxazole (**2d**)

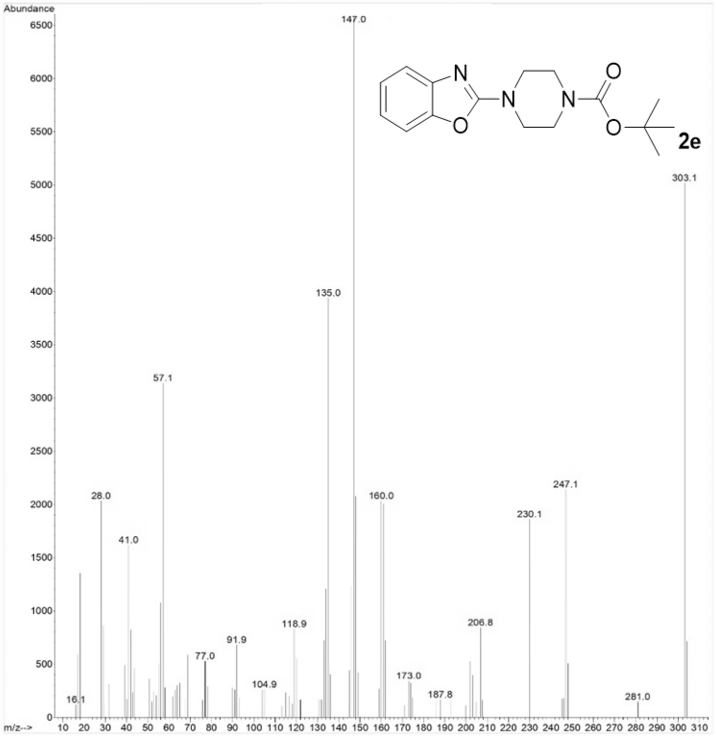

EI-MS of *tert*-butyl 4-(benzoxazol-2-yl)piperazine-1-carboxylate (**2e**)

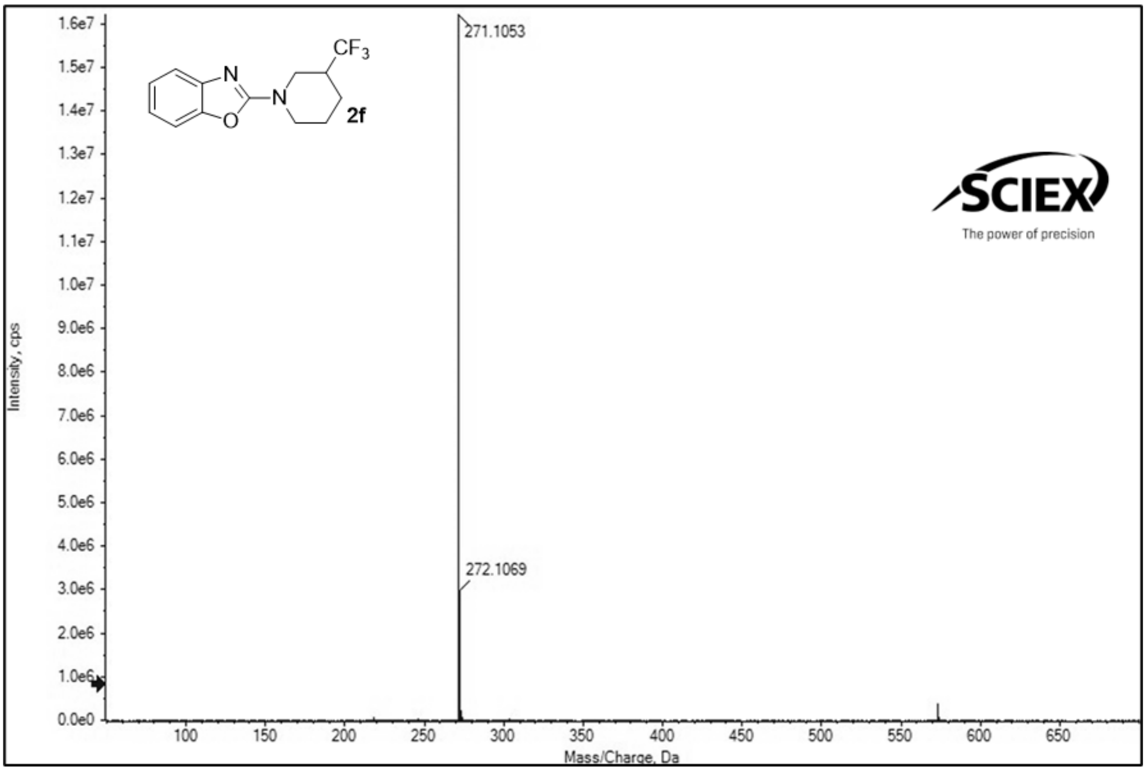

HR-ESI-MS of 2-(3-(trifluoromethyl)piperidin-1-yl)benzoxazole (**2f**)

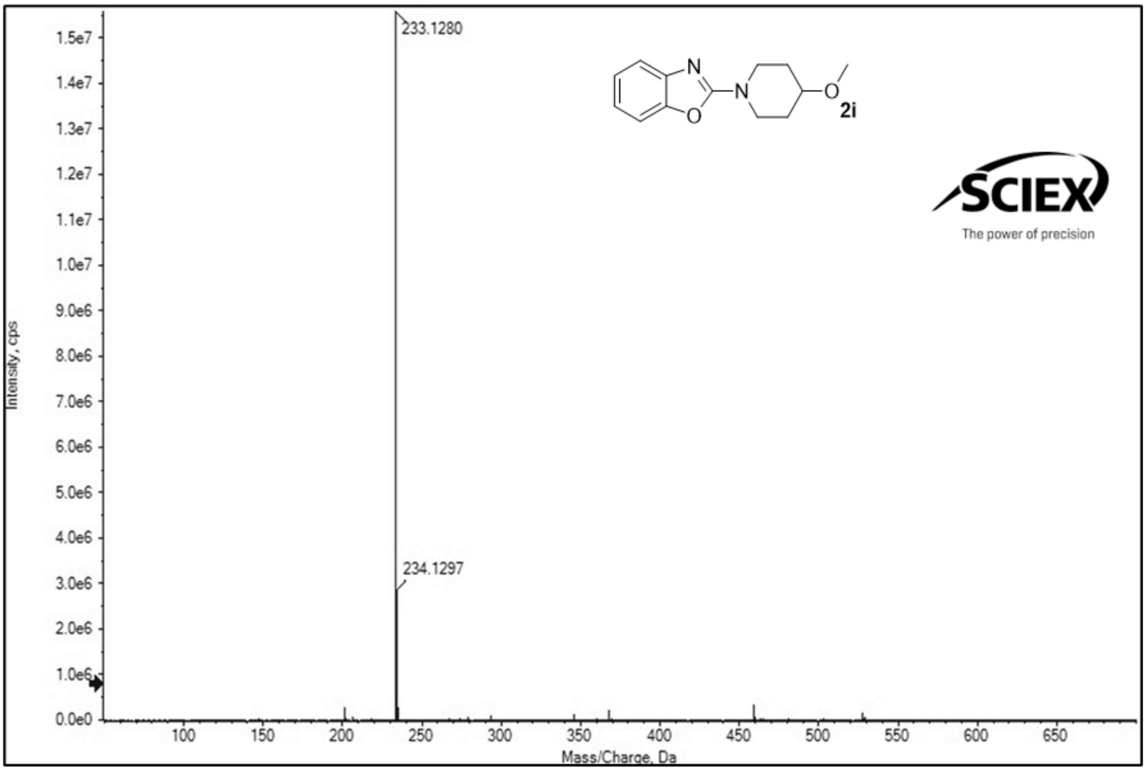

HR-ESI-MS of 2-(4-methoxypiperidin-1-yl)benzoxazole (**2i**)

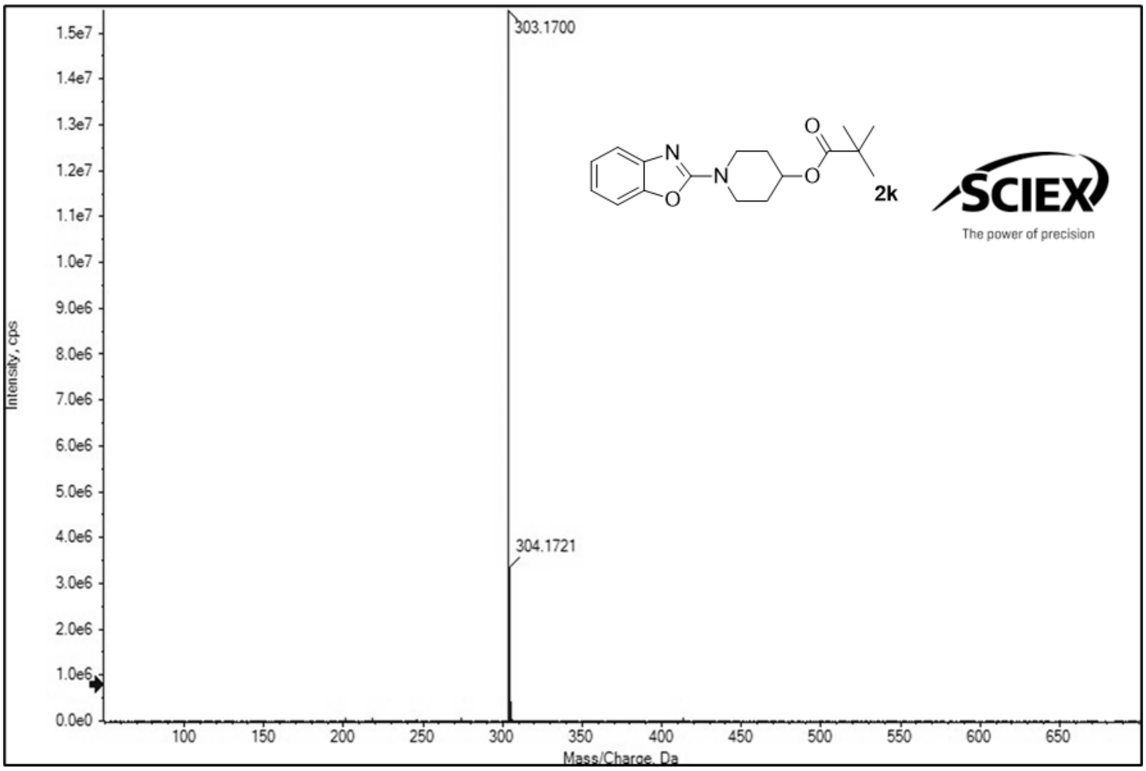

HR-ESI-MS of 1-(benzoxazol-2-yl)-2,2-dimethylpropionic acid piperidin-4-yl ester (**2k**)

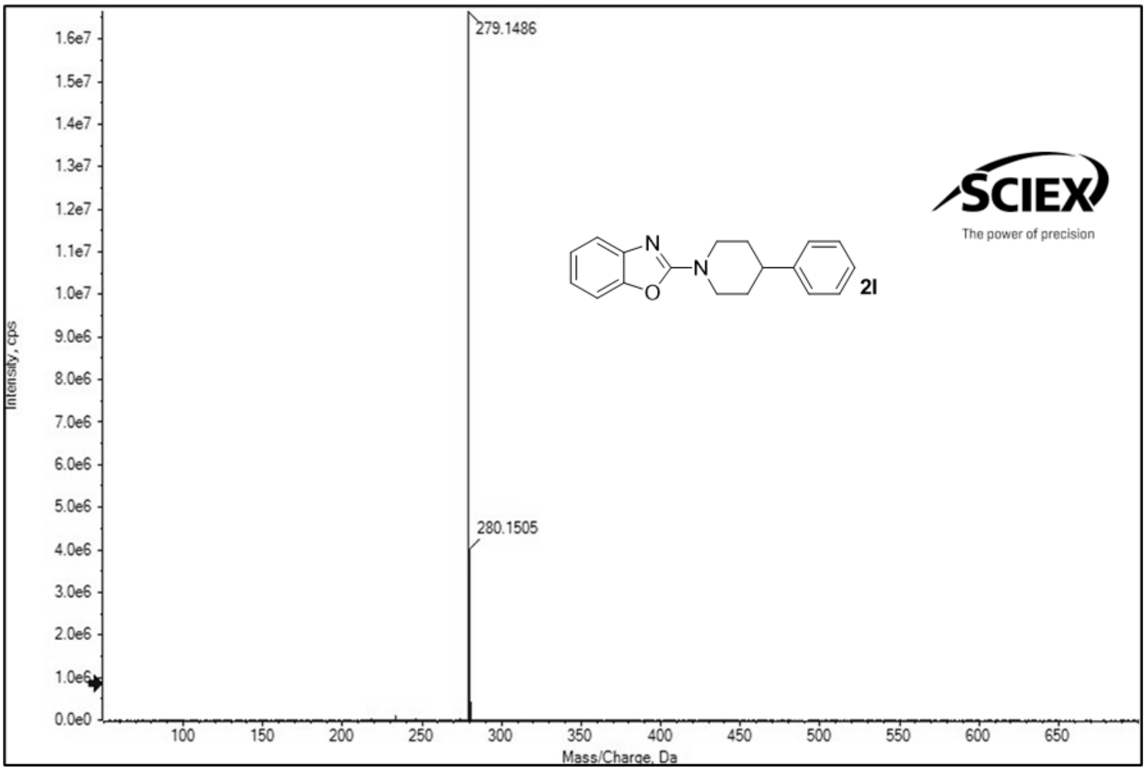

HR-ESI-MS of 2-(4-phenylpiperidin-1-yl)benzoxazole (**2l**)

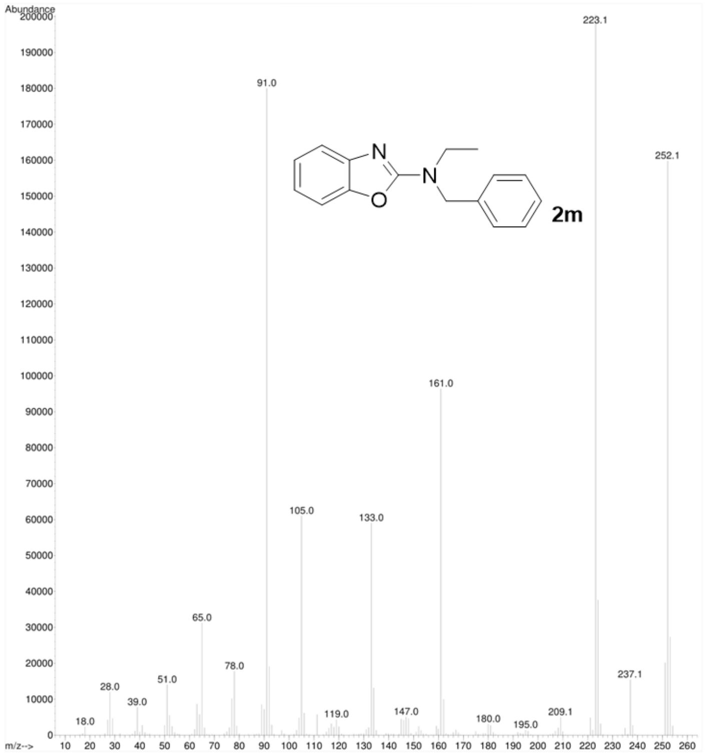

EI-MS of *N*-benzyl-*N*-ethylbenzoxazol-2-amine (**2m**)

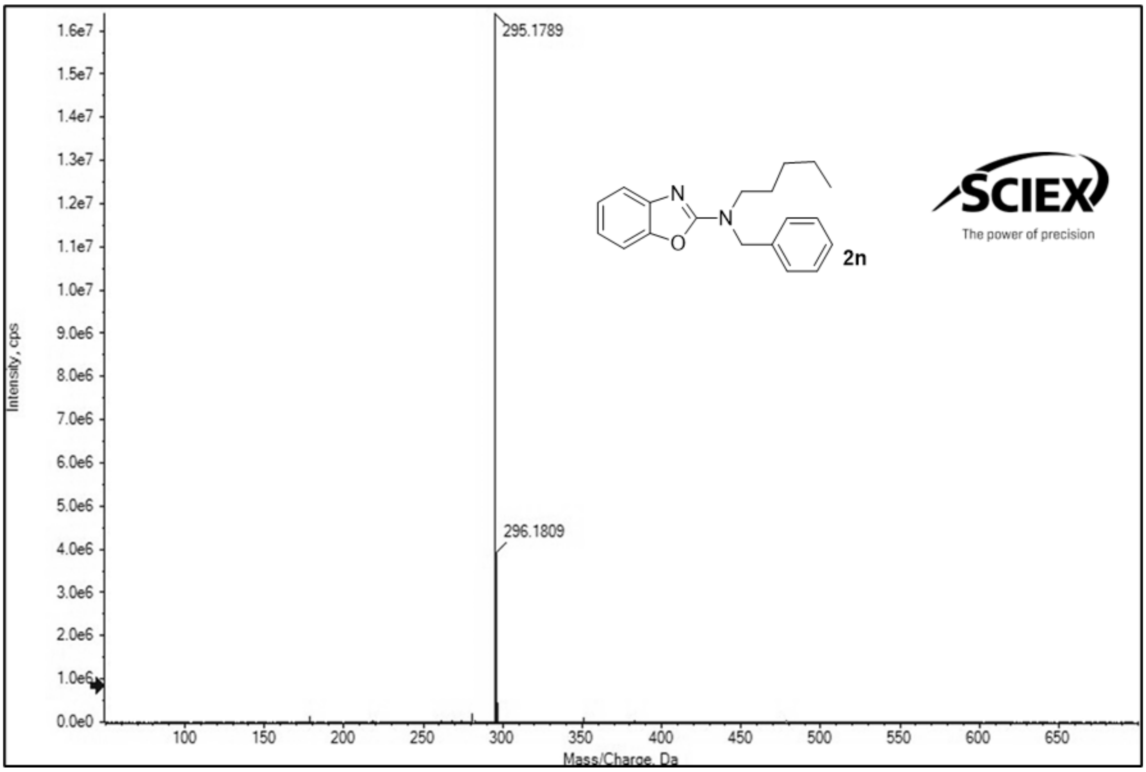

HR-ESI-MS of *N*-benzyl-*N*-pentylbenzoxazol-2-amine (**2n**)

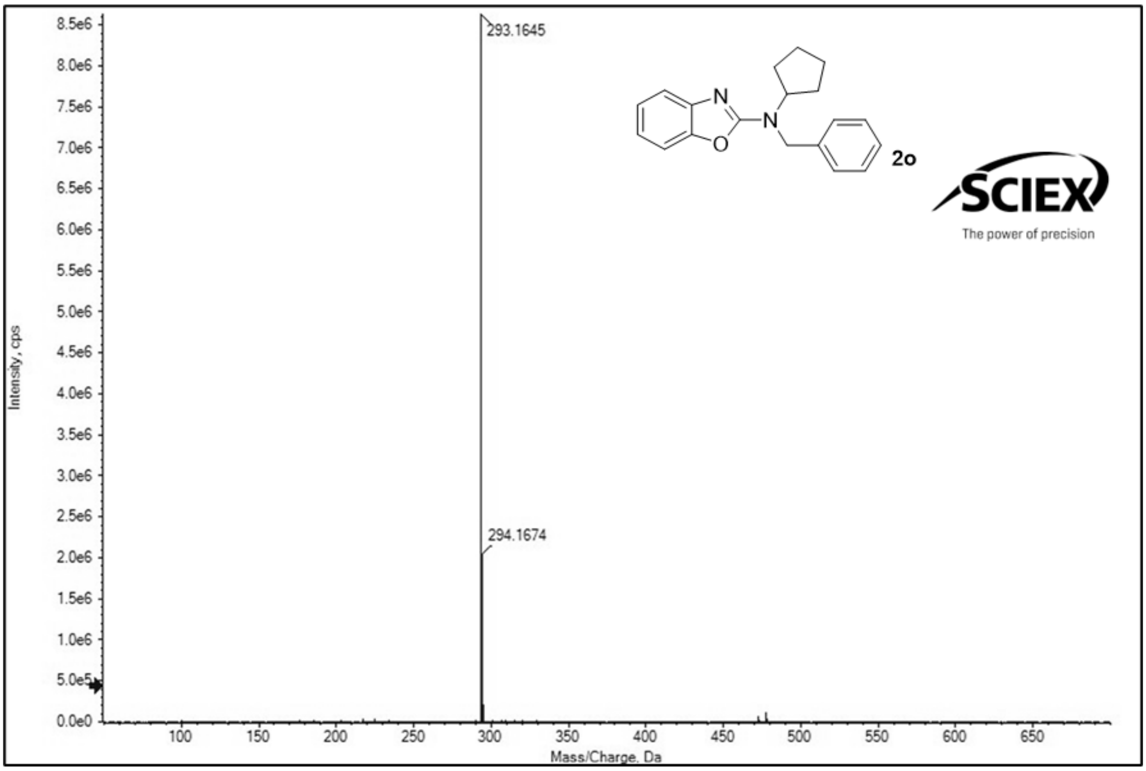

HR-ESI-MS of *N*-benzyl-*N*-cyclopentylbenzoxazol-2-amine (**2o**)

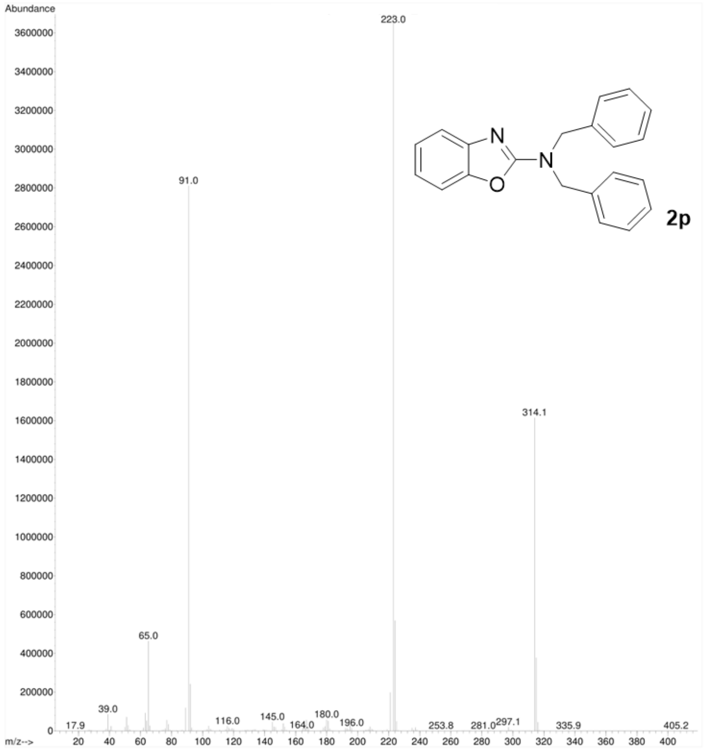

EI-MS of *N*,*N*-dibenzylbenzoxazol-2-amine (**2p**)

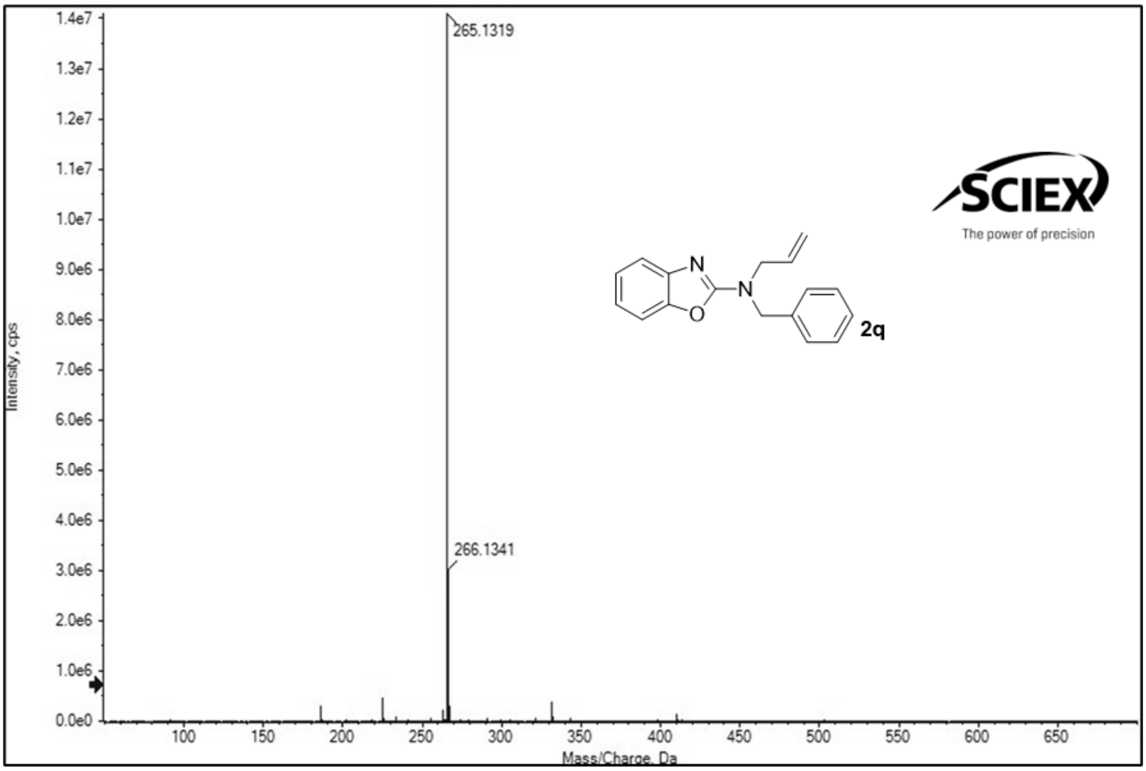

HR-ESI-MS of *N*-allyl-*N*-benzylbenzoxazol-2-amine (**2q**)

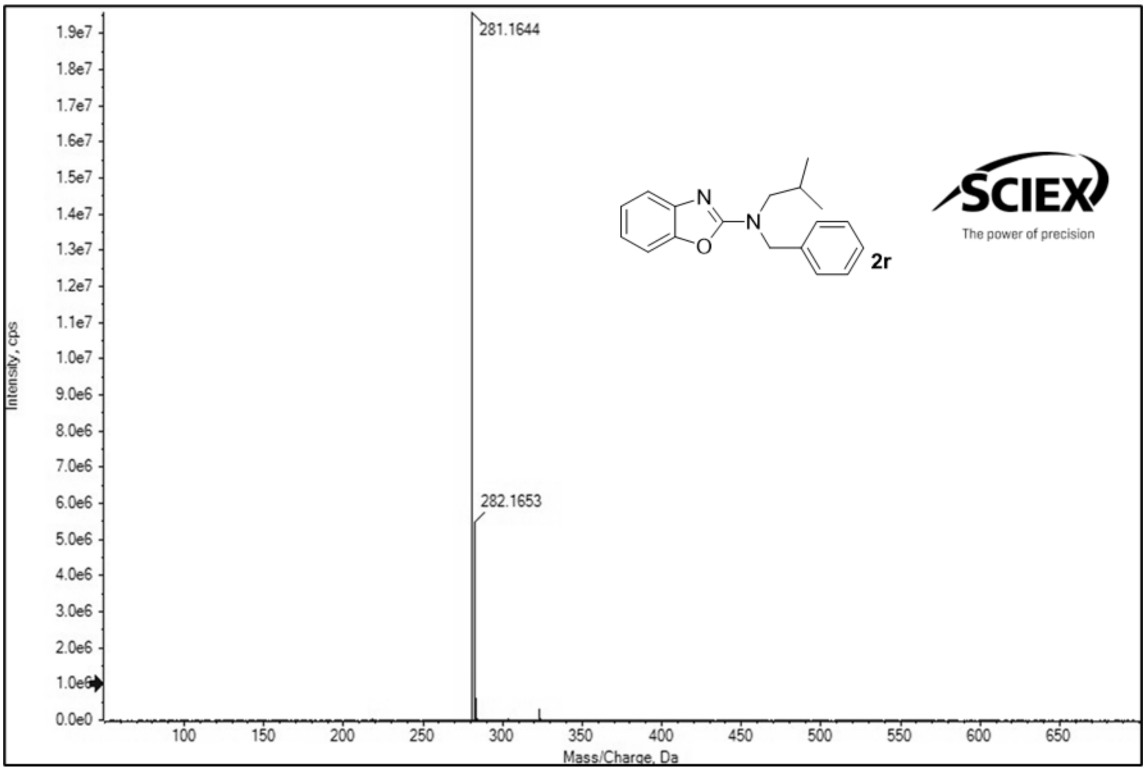

HR-ESI-MS of *N*-allyl-*N*-benzylbenzoxazol-2-amine (**2r**)

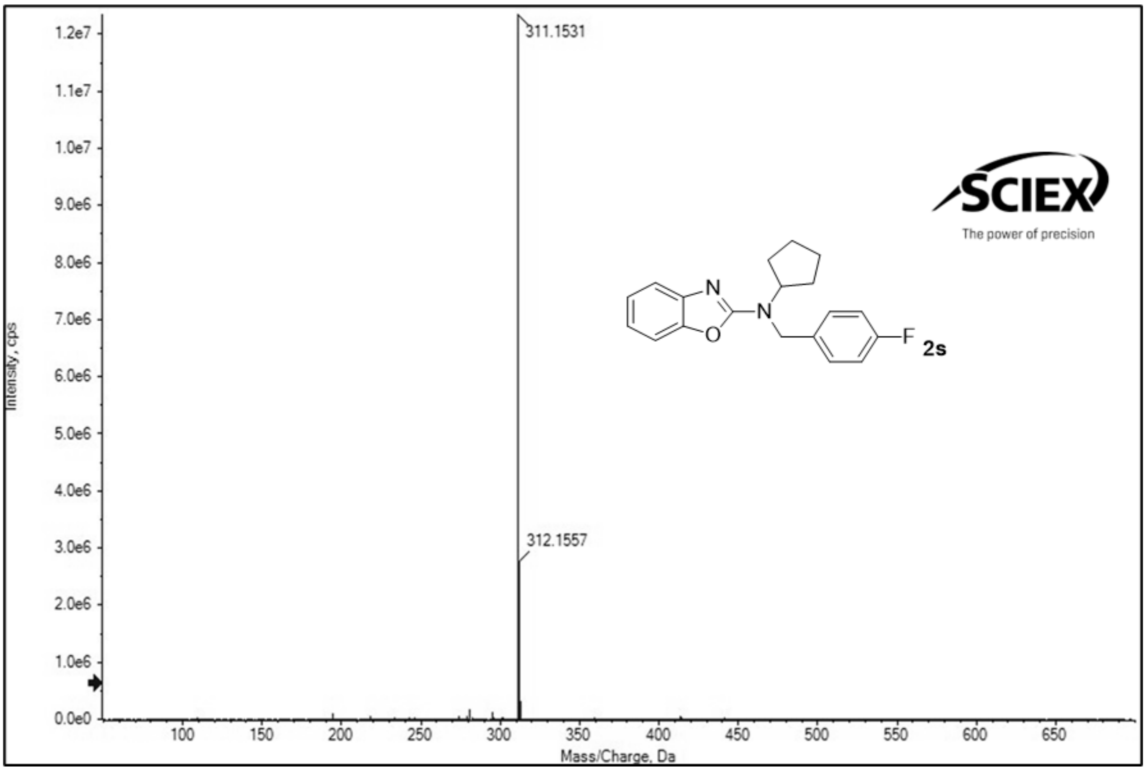

HR-ESI-MS of *N*-cyclopentyl-*N*-(4-fluorobenzyl)benzoxazol-2-amine (**2s**)

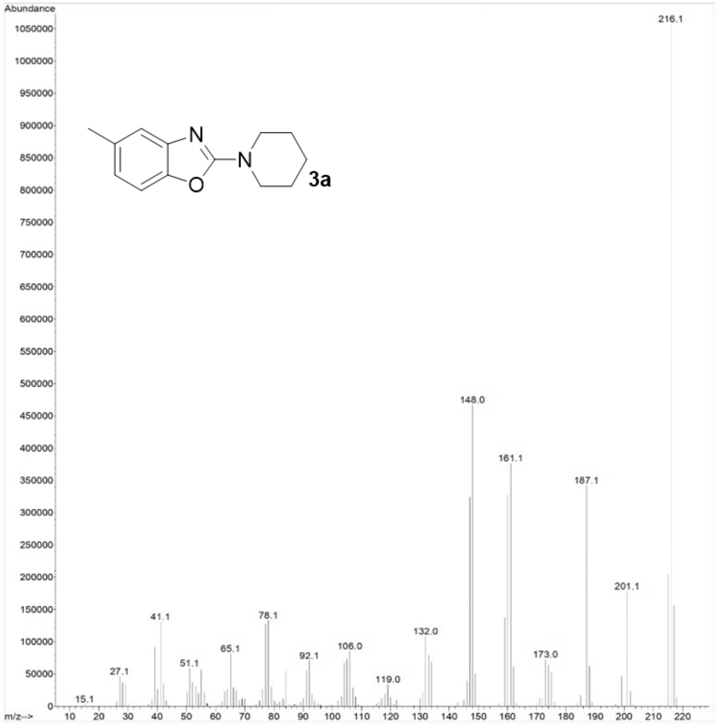

EI-MS of 5-methyl-2-(piperidin-1-yl)benzoxazole (**3a**)

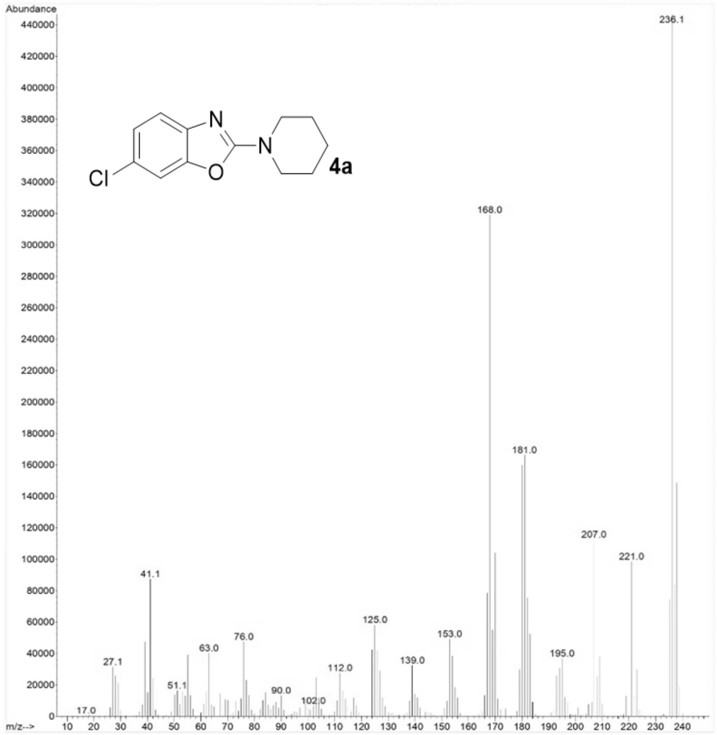

EI-MS of 6-chloro-2-(piperidin-1-yl)benzoxazole (**4a**)

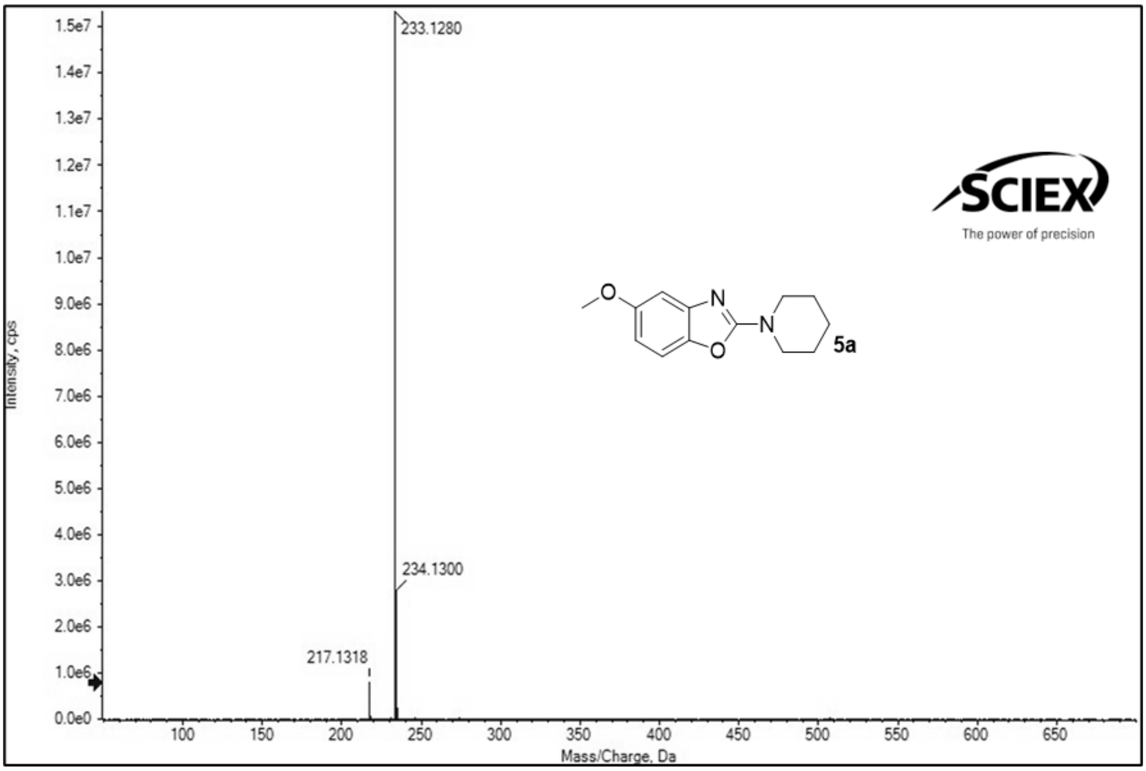

HR-ESI-MS of 5-methoxy-2-(piperidin-1-yl)benzoxazole (**5a**)

References

1. Martina, K.; Calsolaro, F.; Zuliani, A.; Berlier, G.; Chávez-Rivas, F.; Moran, M. J.; Luque, R.; Cravotto, G. *Molecules* **2019**, *24* (13), 2490.
2. Sung, S.; Braddock, D. C.; Armstrong, A.; Brennan, C.; Sale, D.; White, A. J. P.; Davies, R. P. *Chem. - Eur. J.* **2015**, *21* (19), 7179-7192.
3. Chang, D.; Feiten, H.-J.; Engesser, K.-H.; van Beilen, J. B.; Witholt, B.; Li, Z. *Org. Lett.* **2002**, *4* (11), 1859-1862.
4. Shao, Z.; Fu, S.; Wei, M.; Zhou, S.; Liu, Q. *Angew. Chem. Int. Ed.* **2016**, *55* (47), 14653-14657, https://doi.org/10.1002/anie.201608345.
5. Ramachandran, P. V.; Choudhary, S. *Chem. Comm.* **2022**, *58* (84), 11859-11862, 10.1039/D2CC04173A.
6. Wang, Z.; Chen, S.; Chen, C.; Yang, Y.; Wang, C. *Angew. Chem. Int. Ed.* **2023**, *62* (6), e202215963, https://doi.org/10.1002/anie.202215963.
7. Jing, J.; Huo, X.; Shen, J.; Fu, J.; Meng, Q.; Zhang, W. *Chem. Comm.* **2017**, *53* (37), 5151-5154, 10.1039/C7CC01069A.
8. Huang, P.-Q.; Lang, Q.-W.; Wang, Y.-R. *J. Org. Chem.* **2016**, *81* (10), 4235-4243.
9. Giroud, M.; Harder, M.; Kuhn, B.; Haap, W.; Trapp, N.; Schweizer, W. B.; Schirmeister, T.; Diederich, F. *ChemMedChem* **2016**, *11* (10), 1042-1047.
10. Gao, F.; Kim, B.-S.; Walsh, P. J. *Chem. Comm.* **2014**, *50* (73), 10661-10664, 10.1039/C4CC05307A.
11. Wertz, S.; Kodama, S.; Studer, A. *Angew. Chem. Int. Ed.* **2011**, *50* (48), 11511-11515.
12. Pattarawarapan, M.; Yamano, D.; Wiriya, N.; Phakhodee, W. *J. Org. Chem.* **2019**, *84* (10), 6516-6523.
13. Parmar, U.; Somvanshi, D.; Kori, S.; Desai, A. A.; Dandela, R.; Maity, D. K.; Kapdi, A. R. *J. Org. Chem.* **2021**, *86* (13), 8900-8925.
14. Guo, S.; Qian, B.; Xie, Y.; Xia, C.; Huang, H. *Org. Lett.* **2011**, *13* (3), 522-525.
15. Lamani, M.; Prabhu, K. R. *J. Org. Chem.* **2011**, *76* (19), 7938-7944.
